# Spatial Dynamics of Evolving Dosage Compensation in a Young Sex Chromosome System

**DOI:** 10.1093/gbe/evv013

**Published:** 2015-01-23

**Authors:** Roland Schultheiß, Heidi M. Viitaniemi, Erica H. Leder

**Affiliations:** Division of Genetics and Physiology, Department of Biology, University of Turku, Finland

**Keywords:** sex chromosome evolution, dosage compensation, Y chromosome degeneration, gene expression, RNA sequencing, threespine stickleback

## Abstract

The loss of Y-linked genes during sex chromosome evolution creates a potentially deleterious low gene dosage in males. Recent studies have reported different strategies of dosage compensation. Unfortunately, most of these studies investigated taxa with comparatively old sex chromosome systems, which may limit insights into the evolution of dosage compensation and thus into the causes of different compensation strategies. Using deep RNA sequencing, we investigate differential expression patterns along the young XY chromosomes of threespine sticklebacks. Our strata-specific analyses provide new insights into the spatial patterns during the early stages of the evolution of dosage compensation. In particular, our results indicate systematic upregulation of male gene expression in stratum II, which in turn causes female hypertranscription in the same stratum. These findings are consistent with theoretical predictions that selection during early stages of sex chromosome evolution is stronger for a compensating upregulation in males than for the countercompensation of female hyperexpression. In contrast, no elevated gene expression is detectable in stratum I. We argue that strata-specific differences in compensating male gene expression may evolve in response to differences in the prevailing mechanism of Y chromosome degeneration.

## Introduction

The suppression of recombination between the X and Y chromosomes and the subsequent Y degeneration are thought of as key stages in the evolution of heteromorphic sex chromosomes (Charlesworth 1996; Charlesworth et al. 2005). The loss of Y-linked genes is challenging for the heterogametic sex (i.e., males in XY systems) because the resulting drop in expression levels causes a potential disruption of the fine-tuned stoichiometric balance between autosomal and X-linked gene products (Bachtrog et al. 2011). Hence, a chromosome-wide upregulation of the male X copy was predicted to compensate for the sex chromosome monosomy (Ohno 1967). However, recent studies have cast doubt on this concept of global dosage compensation by reporting cases where genes are individually compensated (Ellegren et al. 2007; Mank and Ellegren 2009; Vicoso and Bachtrog 2011; Harrison et al. 2012; Julien et al. 2012). Unfortunately, the comparatively old age of most of the studied sex chromosome systems prevents more detailed insights into the critical early stages of the evolution of dosage compensation.

A study of the young XY system of the plant *Silene latifolia* provides limited evidence that dosage compensation can evolve quickly, that is, within 10 Myr, by increasing the expression of X-linked genes in response to the ongoing degeneration of the Y (Muyle et al. 2012). The authors argue that compensating mechanisms start evolving as soon as Y expression declines (but see Chibalina and Filatov 2011). This pattern is however not consistent. For example, the chicken Z chromosome shows no sign of selection for dosage compensation, neither on its youngest (between 34 and 54 Myr old) nor on the older strata (Wright et al. 2012). In fact, expression levels in males (i.e., the homogametic sex in the avian ZW sex chromosome system) increase with stratum age, which is more concordant with male-biased selection than with dosage compensation. In contrast, the youngest stratum in the mammalian X chromosome exhibits the highest proportion of genes escaping X chromosome inactivation (Carrel and Willard 2005). This, in turn, is consistent with the hypothesis of optimized dosage compensation in the older strata but not yet fully developed dosage compensation in the youngest stratum. The question arises as to when and how chromosome-wide or local compensating mechanisms evolve (Mank 2013). The analysis of the spatial dynamics of dosage compensation during the early stages of sex chromosome evolution may help elucidate this question.

**Fig. 1.—:**
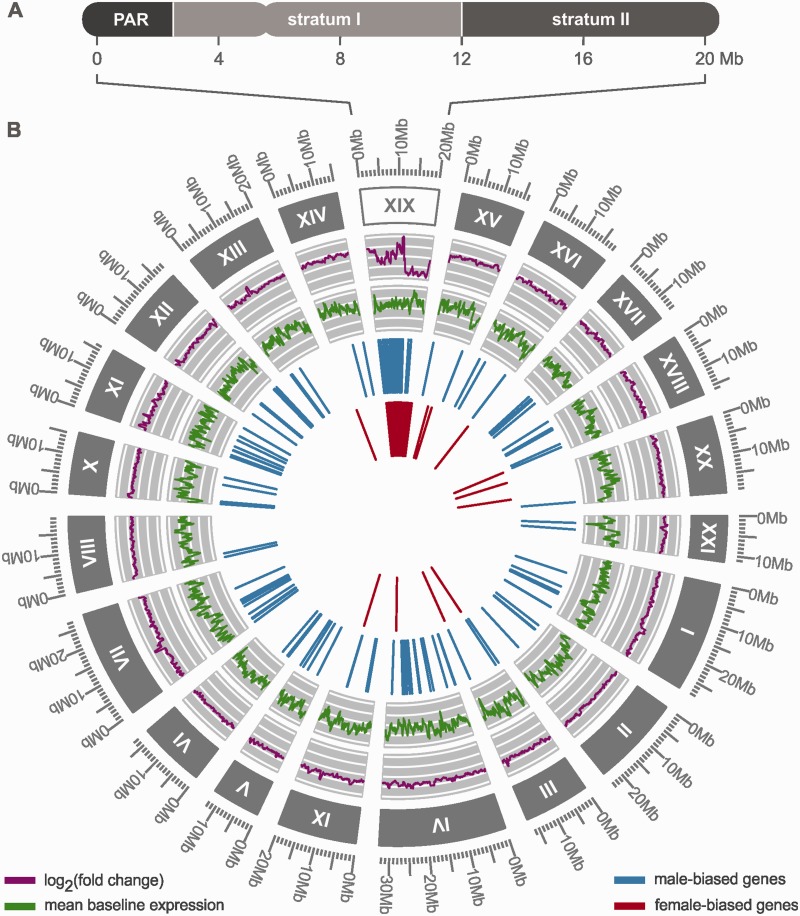
Spatial overview of the transcriptome and the sex chromosome (XIX) of the threespine stickleback. (*A*) The X chromosome of the threespine stickleback consists of three regions: The PAR, stratum I, and stratum II. (*B*) The transcriptome was assembled from a total of 30,336 genes. Chromosomes are labeled with roman numbers and sizes are provided in mega bases (Mb; unmapped scaffolds are not shown). The expression fold-change of genes and their mean baseline expression level as reported from the DESeq2 analysis were smoothed for this figure using a sliding window approach implemented in the R package “zoo” (Zeileis and Grothendieck 2005). The inner two circles show the spatial distribution of genes with male- and female-biased expression along the transcriptome. The figure was generated using the R package “ggbio” (Yin et al. 2012).

This study targets these spatial dynamics by investigating expression patterns in the young sex chromosome system of the threespine stickleback, *Gasterosteus aculeatus* (Peichel et al. 2004). The XY sex chromosome system of the threespine stickleback evolved after the split from the ninespine stickleback (*Pungitius pungitius*) (Ross et al. 2009) at least 13 Ma, which—in light of the different sex chromosome systems of these genera—constitutes an approximate age constraint for the sex chromosomes of the threespine stickleback (Bell and Foster 1994; Ross and Peichel 2008; Bell et al. 2009). This age is comparable to that of the young sex chromosome system of *S. latifolia*, but the availability of the threespine stickleback genome enables a more detailed investigation of the spatial dynamics during the evolution of dosage compensation. Previous studies have identified three regions in its sex chromosomes based on fluorescence in situ hybridization (Ross and Peichel 2008) and the analysis of recombination data (Roesti et al. 2013): A pseudoautosomal region (PAR) and two regions of differential Y degeneration (i.e., two evolutionary strata). The PAR covers approximately the first 2.5 million base pairs (Mb) and recombines in both sexes. Stratum I lies adjacent to the PAR and extends to 12 Mb, and stratum II, which covers the remainder in chromosome XIX, is largely absent from the Y due to a deletion of approximately 6 Mb (Ross and Peichel 2008) (fig. 1*A*). The sudden deletion of large blocks of the Y chromosome in stratum II and the lack of such a deletion in stratum I offer a prime opportunity to evaluate the effects of differential Y degeneration on dosage compensation. Charlesworth (1996) discussed two strategies of dosage compensation: A locus-by-locus mode, individually enhancing gene expression at randomly distributed loci that undergo selective sweeps, and a blockwise mode, changing gene expression over large blocks of the chromosome degenerating due to Muller’s ratchet.

We generated RNA sequencing data of brain tissue samples from both sexes of four different populations to analyze sex-specific expression levels along the stickleback transcriptome. In particular we were interested in strata-specific compensating mechanisms of male gene expression in response to differential Y chromosome degeneration, which provides new insights into patterns and processes during early stages of dosage compensation evolution.

## Materials and Methods

### Sampling, Sequencing, and Assembly

Specimens of threespine sticklebacks (*G. aculeatus*) were caught in four Alaskan lakes during breeding season in June 2010 (lakes Bear Paw, Lynne, Corcoran, and South Rolly). RNA from whole brain tissue was extracted with a phenol-based phase separation (Tri-reagent, Ambion) following the Trizol-protocol from SIGMA with minor modifications, that is, replacing chloroform with 1-Bromo-3-chloropropane and adding an extra ethanol wash for the RNA. For every lake population, we pooled an equivalent amount of RNA from five specimens per sex to produce representative male and female pools for each lake. This amounts to a total of eight samples (i.e., two sexes for four lakes). The sample libraries were created with Illumina TruSeq reagents and were sequenced using 100 base paired-end sequencing in two separate lanes (each containing two male and two female samples with individual labels) on an Illumina HiSeq 2000 platform at the Beijing Genomics Institute. A total of approximately 45 million reads per sample were trimmed for low quality bases using the program ConDeTri with default settings (Smeds and Künstner 2011). The retained reads were mapped with Tophat 2.0.12 (Trapnell et al. 2012) against a custom modified genome (*G. aculeatus* v. 67, Ensembl) where the reported flip in the orientation of the last two supercontings in chromosome XIX was corrected (Ross and Peichel 2008). The mapped reads were then assembled into a transcriptome using Cufflinks 2.2.1 (Trapnell et al. 2012).

### Differential Expression Analyses

We investigated two forms of differential expression: (1) Sex-specific gene expression and (2) differential gene expression between autosomal and sex-linked genes. Due to the absence of a Y chromosome sequence, it is not possible to unambiguously identify all reads originating from the Y. Hence, the reads from each male sample in the subsequent differential expression analyses likely contain reads from both the X and Y chromosomes (but see subsection “Sex Chromosome Assignment” for an approach to identify expression levels of genes expressed only from the male X chromosome, i.e., where the Y copy is lost or silenced).

1. Sex-specific expression: For the analysis of differential sex-specific gene expression (referred to in the text as XY:XX analysis) we used the R package “DESeq2” 1.4.5 on untransformed read counts, which were generated with “HTSeq” (Anders and Huber 2010; R Core Team 2013). After adjusting the *P* values for multiple testing (Benjamini and Hochberg 1995), genes with adjusted *P* values below 0.05 were accepted as significantly differentially expressed. The analysis includes the individual normalization of the read count of each sample based on the library size.
2. Differential gene expression between sex chromosomal and autosomal genes: Expression differences between sex chromosomal and autosomal genes were assessed by pooling the four sample populations of both sexes (i.e., XY:AA and XX:AA). We calculated ratios of median expression levels for a) the entire sex chromosome, b) the PAR, c) stratum I, and d) stratum II to the median expression level of all autosomal genes, respectively (“observed values” in fig. 3 and table 2). Read counts were normalized by calculating fragments per kilobase of transcript per million mapped reads (FPKM) using Cufflinks 2.21. Inactive genes and genes with very low expression values (FPKM < 1) were removed from the analysis. To estimate confidence intervals for the ratios of median gene expression levels, the data sets were bootstrapped using the R package “boot” 1.3 (Canty and Ripley 2014). Median ratios of the pooled sex chromosomal and autosomal expression values were calculated for each of the 10,000 bootstrap samples and equitailed two-sided nonparametric 95% confidence intervals of the obtained distributions were calculated with the percentile method (see package documentation). Ratios of expression levels were considered significantly different from 1 (i.e., parity between sex chromosomal and autosomal genes) on a *P* = 0.05 level if the observed values lay outside the confidence intervals.

### Sex Chromosome Assignment

Variant calling was performed with SAMtools 0.1.19 and BCFtools 0.1.19 (Li et al. 2009). Genotype calling was performed simultaneously for all eight RNA samples. This increased the statistical power of the variant allele likelihood estimation compared with the by-sample approach as information to support a variant allele call comes from all eight rather than just one sample. Output of the variant calling was parsed and filtered with a custom Python script: First, all loci with more than one alternative allele or indel were removed. Then the remaining loci were filtered using a sample-specific genotype quality threshold of 20, after which the loci were further filtered based on a Phred-scaled genotype likelihood score threshold of 25. This means that the difference between the likelihood score of the most likely genotype and alternative genotypes at a biallelic locus has to be larger than 25 for the most likely genotype call to be retained in the data set.

Loci that were heterozygous in all male samples but homozygous in all female samples in the sex chromosome were considered Y-specific, that is, these allelic variants in males constitute Y chromosomal variants. In order to estimate expression levels from the male X copy, we identified all genes carrying these Y-specific single nucleotide polymorphisms (SNPs) and removed them from the data set. We then crosschecked and refined our selection of genes without an expressed Y copy by performing variant calling on two male individuals for which we obtained whole-genome sequence data (supplementary material S1, Supplementary Material online). For further analyses, we only retained genes that 1) exhibited Y-specific SNPs neither in the RNA nor in the DNA data set (hemizygous genes) or 2) exhibited Y-specific SNPs in the exons in the DNA data set but not in the RNA (silenced genes). Male expression from these genes is most likely originating merely from the single male X copy, which enables us to approximate an Xmale:AA ratio by repeating the analysis of differential gene expression between sex chromosomal and autosomal genes with this set of genes (henceforth referred to as X-linked genes). In order to test whether any changes in expression levels in these genes were confined to males or were detectable in females, we likewise conducted the analysis with the same set of genes for females. Additionally, we evaluated the robustness of the results of the differential expression analysis by employing criteria of varying rigor for identifying genes with a Y-linked copy. These data are presented in supplementary material S2, Supplementary Material online.

## Results

We obtained RNA sequencing data from male and female brain tissue of four Alaskan lake populations of *G. aculeatus* and mapped the reads against a custom-modified genome (see Materials and Methods). Two differential expression analyses were performed based on normalized read counts: 1) Differential expression between the sexes and 2) differential expression between genes in the sex chromosome and genes in the autosomes for each sex, respectively. The analysis of the assembled transcriptome data identified 26,093 genes in the autosomes, 4,243 genes in unmapped scaffolds (covering 3.5 mega bases), and 1,464 genes in the sex chromosome (fig. 1). We found 178 genes in the PAR, 658 genes in stratum I, and 628 genes in stratum II. The gene density in the three regions ranged from 69.3 to 78.5 genes per million base pairs (table 1). Based on variant calling we identified a total of 1,557 loci in the sex chromosome that were heterozygous in each of our four male samples but homozygous in all female samples (see Materials and Methods). These SNPs were distributed across 233 genes in the sex chromosomes (to which we henceforth refer as genes with a transcribed Y-linked copy). Evaluating the RNA and DNA variant calling results yielded 865 exclusively X-linked genes in the two evolutionary strata, that is, 420 hemizygous genes and 445 genes with a silenced Y copy (table 1). The remaining 421 genes in both strata had an ambiguous Y copy status (e.g., Y-specific SNPs present in introns only) and were thus excluded from differential gene expression analyses between the sex chromosome and the autosomes.

**Table 1:** Number and Location of Genes with Sex-Biased Expression

	Autosomes	Sex Chromosome			
		Total	PAR	Stratum I	Stratum II
Male-biased	126	55	2	50	3
Female-biased	11	488	1	158	329
Total genes	26,093	1,464	178	658	628
Gene density	65.1	73.2	71.2	69.3	78.5
Y genes	NA	233	1	194	38
X-linked genes	NA	865 (445)	NA	379 (18)	486 (427)

Note.—Gene density, number of genes per million base pairs; Y genes, genes with a transcribed Y-linked copy; X-linked genes, genes without an expressed Y-linked copy (numbers in parentheses are the proportion of hemizygous genes; the remainder are the number of silenced Y genes).

### Differential Gene Expression between Sexes

**Fig. 2.—:**
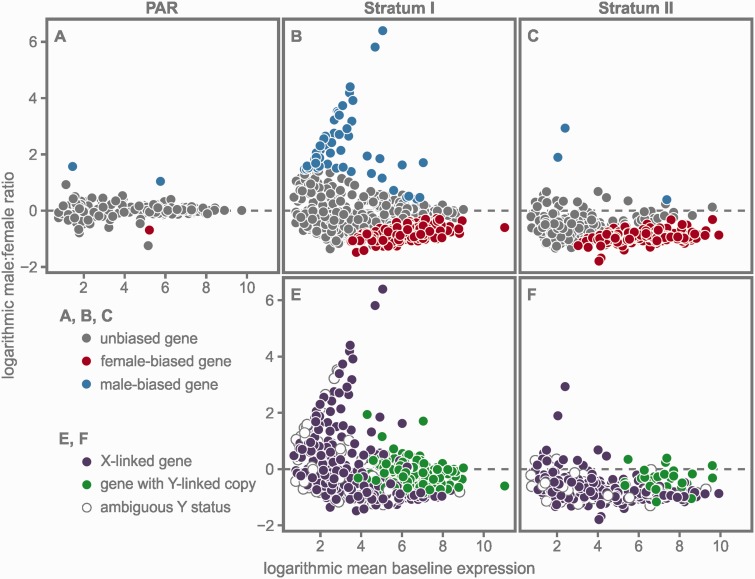
Results of the sex-specific differential expression analyses (XY:XX) for each of the three regions in the sex chromosome: The PAR, stratum I, and stratum II. (*A–C*) Genes with male-biased expression levels are shown in blue, with female-biased expression levels in red. (*E*, *F*) Genes carrying Y-specific SNPs (i.e., genes with a transcribed Y-linked copy) are shown in green for each stratum. Genes without a transcribed Y-linked copy, that is, X-linked genes, are depicted in purple. Empty circles indicate genes of ambiguous Y status (see Results).

Within the transcriptome, we identified 727 genes with significant differential expression between the sexes. Of the 502 genes with female-biased expression, 11 were located in the autosomes, 3 in unmapped scaffolds, and 488 in the sex chromosome. The 225 genes with male-biased expression were distributed more evenly: 126 were located in the autosomes, 44 in unmapped scaffolds, and 55 in the sex chromosome (table 1, fig. 1*B*). The PAR recombines in both sexes, and thus *cis*-regulated expression differences between the sexes are less likely to evolve in this region. Accordingly, we found only two male-biased genes, one female-biased gene (fig. 2*A*), and one gene carrying a Y-specific SNP in the PAR (table 1).

The majority of differentially expressed genes between the sexes was confined to the two sex-linked strata (table 1). Stratum I harbored the majority of genes with male-biased expression (50 of 55), and logarithmic fold changes were highly variable among these genes (fig. 2*B*; mean: 2.27, standard deviation [SD]: 1.24). In total, 158 genes in stratum I exhibited female-biased expression levels. Their mean logarithmic fold change was −0.79 and their variance was much smaller than that of the male-biased genes (SD: 0.24; fig. 2*B*). Almost one-third of the identified genes in stratum I maintained a transcribed Y-linked copy (194 of 658; table 1). Most of these genes had high average expression levels, whereas exclusively X-linked genes had mostly moderate or low expression levels (fig. 2*E*).

Stratum II harbored only three genes with male-biased expression, whereas the majority of genes had female-biased expression (329 of 628; table 1; fig. 2*C*). The variance of the fold changes of the female-biased genes was again comparatively low (SD: 0.14) and the logarithmic mean fold change of these genes was −0.93. As a large part of stratum II is deleted from the Y (Ross and Peichel 2008), the number of genes with a transcribed Y-linked copy was expectedly much lower than in stratum I (i.e., 38 of 628 genes; table 1). However, the average expression levels of these genes were—as in stratum I—comparatively high (fig. 2*F*).

### Differential Gene Expression between the Sex Chromosome and the Autosomes

We calculated the ratio of the median sex chromosomal expression level (i.e., XX for females and XY for males) to the median autosomal expression level (AA) using FPKM-normalized count data. The analysis was conducted with four different data sets: All sex chromosomal versus autosomal genes, PAR versus autosomal genes, stratum I versus autosomal genes, and stratum II versus autosomal genes. To estimate confidence intervals, we generated 10,000 pooled bootstrap samples of each data set and recalculated the ratios of medians as well as the 95% confidence intervals for the distributions.

**Fig. 3.—:**
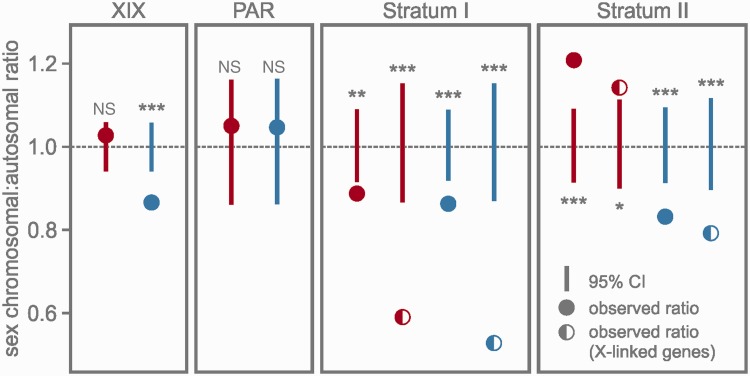
Ratios of median expression levels of sex-chromosomal and autosomal genes (XYmale:AA, XXfemale:AA). The ratios are provided for the entire sex chromosome (XIX) and the three chromosomal regions, and are depicted as color-coded circles (red: females, blue: males). For the two evolutionary strata we also provide the ratios of median expression levels of exclusively X-linked genes for both sexes, depicted as color-coded semicircles. Vertical lines indicate 95% confidence intervals (CIs) obtained by bootstrapping the data sets and calculating the ratio of medians for each of the 10,000 bootstrap samples. Statistical significances of the observed ratios are coded as follows: NS (not significant); *P < 0.05; **P < 0.01; ***P < 0.001.

The observed ratio of median sex-linked and autosomal expression levels for the entire sex chromosome was 1.03 for females (fig. 3, table 2), indicating that expression levels in females are not significantly different for genes in the sex chromosome and in the autosomes. Males however had a significantly lowered overall median expression level in the sex chromosome (0.87, *P* < 0.001; table 2). Although the observed ratios in the recombining PAR were close to parity in both sexes (males and females: 1.05; fig. 3), the two sex-linked strata showed statistically significant differences between the sexes (confidence intervals and *P* values are provided in table 2). In stratum I, the observed XXfemale:AA ratio was 0.89 and the XYmale:AA ratio was 0.86 (fig. 3). In order to isolate gene expression levels of the male X copy, we repeated the analysis with the 379 exclusively X-linked genes in stratum I. The resulting median Xmale:AA ratio was 0.53 and thus considerably lower than for the XYmale:AA comparison (fig. 3). The same set of X-linked genes yielded a median ratio of 0.59 in females, again considerably lower than the median ratio of the entire female data set for stratum I (i.e., 0.89).

**Table 2:** Observed Median Ratios of Gene Expression between Sex Chromosomal (XY/XX) and Autosomal (AA) Genes

	Observed	Upper 95% CI	Lower 95% CI
XYmale:AA PAR	1.05	1.17	0.86
XYmale:AA stratum I	0.86***	1.09	0.92
Xmale:AA stratum I (X-linked)	0.53***	1.15	0.87
XYmale:AA stratum II	0.83***	1.09	0.91
Xmale:A stratum II (X-linked)	0.79***	1.11	0.90
XYmale:AA XIX	0.87***	1.06	0.94
XXfemale:AA PAR	1.05	1.16	0.86
XXfemale:AA stratum I	0.89**	1.09	0.92
XXfemale:AA stratum I (X-linked)	0.59***	1.16	0.86
XXfemale:AA stratum II	1.21***	1.09	0.91
XXfemale:AA stratum II (X-linked)	1.14*	1.12	0.90
XXfemale:AA XIX	1.03	1.06	0.94

Note.—X-linked, set of genes without a transcribed Y copy.

Statistical significances of the observed ratios are coded as follows: *P < 0.05; **P < 0.01; ***P < 0.001.

In stratum II the observed median XXfemale:AA ratio was 1.21 (fig. 3), indicating that female gene expression levels on this stratum are significantly higher than in the autosomes (table 2). The XYmale:AA ratio was 0.83 and was thus similar to the XYmale:AA ratio observed in stratum I. The analysis of the 486 X-linked genes yielded an Xmale:AA ratio of 0.79 (fig. 3). Thus, X-linked genes in stratum II are overall more highly expressed than in stratum I (i.e., 0.79 vs. 0.53). Restricting the analysis to this set of X-linked genes in females yielded a ratio of 1.14, again constituting a statistically significant hyperexpression in females in stratum II.

## Discussion

The analysis of expression differences between the sexes (fig. 2*A*–*C*), and between genes in the entire sex chromosome and genes in the autosomes (fig. 3) is consistent with previous results (Leder et al. 2010) and with the prevailing picture of incomplete dosage compensation, that is, a ratio of less than 1 in the heterogametic sex and of near parity in the homogametic sex (e.g., Itoh et al. 2007; Mank 2013; Uebbing et al. 2013). However, separate analyses of expression levels of genes located in the two evolutionary strata reveal a more complex pattern of sex-biased gene expression and dosage compensation.

Sex-biased expression in the sex chromosome can arise due to dosage differences or due to a past history of sexually antagonistic selection (Mank 2009; Parsch and Ellegren 2013). The former is a direct consequence of the uncompensated degeneration of the Y chromosome. As a large part of stratum II has been deleted from the Y chromosome and as it exhibits substantially fewer transcribed genes (i.e., genes with transcribed Y-linked copies; table 1) than stratum I, Y degeneration has most likely progressed further in stratum II than in stratum I. Yet, the examination of the results of the XYmale:AA analysis for both strata yields very similar ratios (0.86 and 0.83; fig. 3, table 2). This appears to be counterintuitive as we would expect that the less degenerated Y stratum I yields an overall higher XYmale:AA ratio than stratum II because of its higher proportion of genes with a transcribed Y-linked copy. However, when approximating Xmale:AA ratios by analyzing exclusively X-linked genes in both strata, a clear difference is revealed in the observed ratio of expression levels: In stratum I, the Xmale:AA ratio of expression levels is 0.53 (fig. 3). In stratum II, however, the observed Xmale:AA ratio of expression levels is substantially higher (0.79; fig. 3, table 2). Indeed, this high median expression value of exclusively X-linked genes in stratum II is very close to the overall median XYmale:AA expression values in both strata (stratum I: 0.86; stratum II: 0.83) indicating that the majority of male expression in stratum II originates solely from the single copy genes in the male X chromosome. Thus we provide evidence for a systematic upregulation of exclusively X-linked genes in stratum II and, by extension, for the predicted evolution of dosage compensation after the massive deletion in the stickleback Y chromosome (Ross and Peichel 2008). However, the lost and untranscribed Y copies in stratum II are not entirely compensated (this would yield an Xmale:AA ratio of ∼1). Yet, simulations show that during the early stages of dosage compensation any level of upregulation will be favored by selection over no compensation (Hall and Wayne 2013). This is consistent with our findings that the young stickleback sex chromosome system exhibits a locally confined initial upregulation in the male X copy to counter the massive loss of Y-linked genes after the deletion in stratum II.

The systematic upregulation of gene expression in stratum II of the male X copy raises the question whether and how this increase in male gene expression levels affects female expression levels. As discussed above, the median Xmale:AA expression level in stratum I is substantially lower than the observed median XXfemale:AA expression level (fig. 3; note that although the median XXfemale:AA ratio is below 1 the deviation is within the range reported from other taxa and may vary among tissues, e.g., Julien et al. 2012; Uebbing et al. 2013; Smith et al. 2014). Remarkably, the substantial increase of expression levels of X-linked genes in males in stratum II (0.79 compared with 0.53 in stratum I) is accompanied by substantial hypertranscription in females (1.21). This hypertranscription remains statistically significant even after reducing the female data set to the corresponding exclusively X-linked genes found in the males (1.14, *P* < 0.05; fig. 3, table 2). We thus argue that the upregulation in the male X copy is not confined to males—as described for *Drosophila* (Gelbart and Kuroda 2009)—and triggers the observed significant female hypertranscription in stratum II. These findings are in concordance with the prediction of Hall and Wayne (2013) who argue that selection is stronger for a compensating upregulation of low gene expression in the heterogametic sex than for a countercompensation of the resulting hyperexpression in the homogametic sex. Additionally, the presence of upregulated genes related to female function in this tissue may also contribute to observed expression levels on stratum II. However, in the absence of protein data, the interpretation of differences in mRNA expression levels has to be taken with caution as their impact on gene product quantities also depends on the length of regulatory pathways, sex chromosome age, and selection strength (Oliver 2007; Vicoso and Charlesworth 2009; Hall and Wayne 2013).

The differences of male expression levels between stratum I and II (see XYmale:AA and Xmale:AA ratios in fig. 3) indicate that a considerable portion of total male XY expression level in stratum I is generated by genes with a transcribed Y-linked copy. Theory predicts that suppression of recombination leads to an accumulation of male-beneficial mutations in the Y chromosome (Bachtrog 2013). It is thus conceivable that a number of Y homologs in stratum I may have evolved under sexually antagonistic selection and are now highly expressed. Yet this hypothesis is not directly testable with the data at hand because—although allele counts for X and Y variants in genes with a Y-linked copy appear to be mostly equal in proportion—we cannot infer the individual contribution of the X and the Y copy to the overall male expression level in a manner that would be comparable across the genome (see Materials and Methods). However, the overall high expression levels of genes with a transcribed Y-linked copy (fig. 2*E*) offer an alternative explanation for the preservation of these genes in the Y: Assuming that transcription rates are limited, males cannot compensate for the loss of a copy of an initially highly expressed gene by further upregulating their remaining X copy, for example, through modifier genes (Rice 1984; Vicoso and Charlesworth 2009). Hence, selection toward preserving a functioning Y-linked copy of these genes will be strong. This conjecture is consistent with a study on the Y chromosome of *Drosophila miranda* (Kaiser et al. 2011), where gene loss is prevented for genes with male function as well as for highly expressed genes. Overall high expression levels of functional Y homologs were also reported from the human Y chromosome (Sayres and Makova 2013), and a recent study argued that most mammalian Y homologs persisted at least initially because of dosage constraints (Cortez et al. 2014).

It is also noteworthy that 32 of the 50 transcribed genes with male-biased expression in stratum I have no transcribed Y copy (fig. 2*B* and *E*) and consequently are only maintained in the X chromosome. Moreover, the proportion of these X-linked genes among male-biased genes in this stratum (0.64) is higher than the proportion of X-linked genes among unbiased genes (i.e., 0.56; 250 genes of 450). This is in concordance with theoretical models based on dominance that predict an increase of putative male-beneficial mutations in hemizygous regions of the X (Rice 1984). An example is the reported excess of young X-linked male-biased genes in *Drosophila* (Zhang et al. 2010). The authors argue that the proportion of these X-linked male-biased genes will diminish through time leading to an autosomal excess of male-biased genes. This may serve as an explanation for the lack of resident X-linked male-biased genes in stratum II: Only 2 of the 628 genes in stratum II had male-biased expression and were exclusively X-linked genes (fig. 2*C* and *F*). An alternative (but not mutually exclusive) explanation is that the upregulation of the male X in stratum II (fig. 3) may lead to a lack of resident male-biased genes due to the inherent difficulty to further increase transcription in the male X copy (assuming that transcription rates are limited) (Vicoso and Charlesworth 2006, 2009).

Although it is evident that degeneration in the Y has progressed further in stratum II than in stratum I (see also Roesti et al. 2013), this alone allows for no conclusive statement as to the relative age of either stratum. To obtain these ages, we estimated the number of synonymous mutations in the coding regions in the X and Y of both strata in comparison to homologous regions in the ninespine stickleback (details are provided in supplementary material S3, Supplementary Material online). However, examining the spatial distribution and magnitude of the estimated d*S* values provides no conclusive statistical evidence as to the relative ages of the two strata (fig. S3.1 in supplementary material S3, Supplementary Material online). One possible explanation is that recombination between the X and Y in both strata may have stopped in relatively close temporal proximity. Alternatively, crucial differences in d*S* values between the strata may have been erased by the large deletion in the Y, hampering reliable age estimation in this young sex chromosome system. In light of the differences in gene expression between the strata (fig. 3), their age would provide additional insights into the evolution of dosage compensation. For example, if both strata were approximately of the same age or if stratum II was younger, the prevailing mode of Y degeneration (i.e., large instantaneous deletion vs. genewise degeneration) may be more critical for the realized dosage compensation strategy (regional vs. local) than time. Stratum age notwithstanding this hypothesis is in concordance with the observed proportions of hemizygous genes and Y silenced genes in both strata (table 1): In stratum I only 18 of 379 X-linked genes are deleted from the Y (361 are silenced Y genes), in stratum II 427 of 486 are deleted (59 are silenced Y genes). We thus suggest that the compensating regional upregulation in stratum II of the male X may have evolved only after (i.e., in direct response) to the deletion.

Although incomplete dosage compensation in the heterogametic sex has been reported for numerous taxa (Mank 2013), hypertranscription in the homogametic sex as a result of a heterogametic upregulation has only been described from the flour beetle *Tribolium castaneum* (Prince et al. 2010; Mank 2013). As the sex chromosomes in *Tribolium* are comparatively old (>100 Myr), the authors argue that a stable evolutionary solution was achieved with this intermediate stage of dosage compensation and a countercompensation of females is not necessary. In contrast, threespine sticklebacks have considerably younger sex chromosomes and the currently observed expression patterns may not constitute the endpoint of their evolutionary trajectory. It is thus conceivable that in the future females will be forced to countercompensate for the progressing male X upregulation following further Y deterioration (Charlesworth 1996). A potential mechanism to cope with such overexpression is described for *Caenorhabditis elegans*, where males have entirely lost the Y chromosome and the homogametic sex (XX hermaphrodites) reduces transcription of both X copies using a dosage compensation complex (Ercan et al. 2007). A different approach for countercompensation has been described from mammals, where females inactivate one of their X chromosomes (Pessia et al. 2012).

### Summary and Conclusions

Our strata-specific analyses indicate a systematic, partially compensating upregulation of gene expression in the male X copy in stratum II and consequential female hypertranscription. In contrast, stratum I exhibits no female hypertranscription and the median gene expression level from the male X copy is considerably lower than in stratum II. A similar approach to assess the compensation patterns on different sex chromosome strata has been conducted using the avian Z chromosome (Wright et al. 2012), yet the authors found a similar overall ratio of sex chromosome/autosome expression levels, that is, no significant Z:A difference across strata.

The majority of highly expressed genes in stratum I contains a transcribed Y-linked copy and we argue that selection for preserving these copies may be strong because either males cannot compensate for the loss of highly expressed genes and/or the Y-linked genes are male-beneficial. Hence, deterioration of the Y in stratum I is not as advanced as in stratum II, which in turn explains why the male X copy in stratum I is—in contrast to stratum II—apparently not upregulated. These strata-specific gene expression patterns bear witness to the respective different evolutionary trajectories of the strata. More specifically, the patterns may have evolved in response to differences in the prevailing mode of Y chromosome degeneration in each stratum: 1) A successive gene-by-gene degeneration in stratum I, that may allow for a likewise successive gene-by-gene mechanism of dosage compensation, and 2) a deletion of large segments in the wake of chromosome inversions in stratum II (Ross and Peichel 2008). The resulting sudden and widespread drop in gene expression level in the latter case may have triggered the evolution of a regional, systematic upregulation in stratum II.

## Supplementary Material

Supplementary materials S1–S3 are available at *Genome Biology and Evolution* online (http://www.gbe.oxfordjournals.org/).

Supplementary Data
