# Supplementary material for: Spatial Dynamics of Evolving Dosage Compensation in a Young Sex Chromosome System
Source: Genome Biol Evol. 2015 Jan 23;7(2):581–90. doi: 10.1093/gbe/evv013 (PMC4350182; doi:10.1093/gbe/evv013)
Supplement: Supplementary Data [file supp_7_2_581__index.html]

Spatial dynamics of evolving dosage compensation in a young sex chromosome system — Spatial Dynamics of Evolving Dosage Compensation in a Young Sex Chromosome System — Supplementary Data 

# Spatial Dynamics of Evolving Dosage Compensation in a Young Sex Chromosome System

## Supplementary Data

files

**Files in this Data Supplement:**

- Supplementary Data - docx file
- Supplementary Data - docx file
- Supplementary Data - docx file
