# Supplementary material for: Spatial Dynamics of Evolving Dosage Compensation in a Young Sex Chromosome System
Source: Genome Biol Evol. 2015 Jan 23;7(2):581–90. doi: 10.1093/gbe/evv013 (PMC4350182; doi:10.1093/gbe/evv013)
Supplement: Supplementary Data [file supp_evv013_schultheiss_et_al_SUPPLEMENT_1.docx]

# SUPPLEMENT 1: Whole genome variant calling

### Schultheiß et al.: Spatial dynamics of evolving dosage compensation in a young sex chromosome system

###

### Fish sampling

Threespine sticklebacks were collected from Bear Paw Lake, Alaska (USA, 61.6141109448°, -149.7534433°), and from the island of Seili (Finland, 60.241321°, 21.962849°). One male specimen from each population was sequenced.

### DNA extraction and sequencing

DNA for the whole genome sequencing was extracted following a salt extraction method with RNase treatment included. The procedure was as follows: Pipette 200 µl salt extraction buffer (0.4 M NaCl; 10 mM Tris-HCl pH 8.0; 2mM EDTA pH 8.0) and 20 µl of 20 % Sodium Dodecyl Sulfate (SDS) into 1.5 ml Eppendorf tube and added 3 µl of RNAse A (2µg/µl, Promega, Nacka, Sweden) into the tube and incubate in room temperature (RT) for 2-5 minutes. Then another 100 µl salt extraction buffer and 10 µl of 20 % SDS and 5 µl of Proteinase K (Promega) was added into the tube and was left to digest at 60°C for approximately 1.5 hrs. The tube was cooled slightly on ice before adding 3 µl RNAse A and incubating at RT for 10 minutes. To bind the DNA, 225 µl of 6 M NaCl was added to the tube and then centrifuged at 11,000 g for 30 minutes. After this, 525 µl of supernatant was taken from the tube isopropanol was added in 1:1 ratio. The sample was centrifuged at 13,000 rpm for 15 minutes at 4° C. The supernatant was poured out and 300 µl of ice cold 70% EtOH was added and the sample was left to wash overnight. Prior to dissolving the sample, tube was centrifuged at -4°C for 5 minutes at 13,000 rpm. Samples were allowed to dry in RT for 30 minutes after which 30 µl of nuclease free H2O (SIGMA) was added to the tube. Quantity and quality of the DNA samples was assessed with the Nanodrop 1000x (Thermo Scientific, Vantaa, Finland). Samples were stored at 4° C until they were processed at the Finnish Institute for Molecular Medicine Finland (FiMM); DNA sequencing libraries were sequenced with 100PE Illumina GAIIx.

### DNA Sequence mapping, quality filtering, and assignment of genotypes

The custom genome used in the DNA sequence mapping was the same as used for the transcriptomes (i.e. modified from Ensembl build v67 by reverse complementing chromosome XIX positions 3824253-20240659 (see (Ross & Peichel 2008)). Reads were first trimmed using windowed adaptive trimming which uses a sliding window to determine where read quality begins to decline allowing for an average minimum base quality threshold of 20 within a sliding window of 20 bases. Once quality fails to meet this threshold the remaining sequence is trimmed. Trimmed reads shorter than 20 bases were discarded. Reads were mapped with BWA 0.6.1 (Li et al. 2009) against the altered reference genome. After the first mapping, local re-alignment to reduce alignment errors due to insertion-deletions was performed with GATK 2.3.9 (Broad Institute, (DePristo et al. 2011)). Variant calling was done similarly to the transcriptomes using SAMtools 0.1.19 and BCFtools 0.1.19 (Li et al. 2009). Output of the variant calling was parsed and filtered with a custom python script. The script first removes positions with indels and triallelic SNPs, then checks the genotypes for each individual. If the genotype quality is greater than 15 and the Phred scaled genotype likelihood score is zero with the next lowest score being above 24, then the individual genotype is retained, otherwise it is converted to N/N. All loci from transcripts on chromosome XIX remaining after the filtering step were used for identifying heterozygous loci. We used the intersect command from the BEDtools suite v2.17.0 to find variant positions in the chromosome XIX genome sequence corresponding to those in the transcriptome by comparing all SNPs called in chromosome XIX with the gtf file generated from Cufflinks (see Materials and methods and table S1.1 below).

### References

DePristo MA et al. 2011. A framework for variation discovery and genotyping using next-generation DNA sequencing data. Nat Genet. 43:491–498. doi: [10.1038/ng.806](http://dx.doi.org/10.1038/ng.806).

Li H et al. 2009. The Sequence Alignment/Map format and SAMtools. Bioinformatics. 25:2078–2079. doi: [10.1093/bioinformatics/btp352](http://dx.doi.org/10.1093/bioinformatics/btp352).

Ross JA, Peichel CL. 2008. Molecular cytogenetic evidence of rearrangements on the Y chromosome of the threespine stickleback fish. Genetics. 179:2173–2182. doi: [10.1534/genetics.108.088559](http://dx.doi.org/10.1534/genetics.108.088559).

**Table S1.1:** Number of SNPs in each gene in chromosome XIX for each of the two male samples (Alaskan sample in green, Finnish in blue), separated by exon-, intron-, up- and downstream region (the latter two regions extend 2000 base pairs from the begin/end of the transcript). We additionally provide the results of the DESeq2 analysis for these genes.

| **GeneID** | **Base mean** | **Fold change** | **XIX region** | **Exon region** | **Up/downstream region** | **Intron region** | **Exon region** | **Up/downstream region** | **Intron region** |
| --- | --- | --- | --- | --- | --- | --- | --- | --- | --- |
| XLOC_021096 | 234.0417232 | -0.045618755 | PAR | 4 | 2 | 38 | 3 | 10 | 49 |
| XLOC_021655 | 8.156568828 | 0.256231704 | PAR | 0 | 13 | 0 | 2 | 19 | 0 |
| XLOC_021656 | 52.51102158 | 0.185541079 | PAR | 0 | 14 | 33 | 2 | 15 | 29 |
| XLOC_022184 | 43.4641635 | 0.292461829 | PAR | 0 | 12 | 0 | 5 | 10 | 0 |
| XLOC_021657 | 13.13320537 | -0.101481399 | PAR | 1 | 3 | 14 | 2 | 9 | 10 |
| XLOC_021097 | 95.34529041 | 0.121426754 | PAR | 13 | 2 | 6 | 8 | 18 | 19 |
| XLOC_021098 | 4.70794907 | 0.029160093 | PAR | 1 | 0 | 27 | 4 | 12 | 87 |
| XLOC_021099 | 7.342204396 | -0.468471027 | PAR | 3 | 15 | 24 | 2 | 22 | 43 |
| XLOC_022185 | 4.09285561 | -0.145114648 | PAR | 1 | 19 | 0 | 4 | 17 | 0 |
| XLOC_021659 | 1673.801796 | 0.103971112 | PAR | 3 | 16 | 286 | 13 | 32 | 368 |
| XLOC_022186 | 11.67571267 | -0.147294145 | PAR | 3 | 6 | 0 | 5 | 11 | 0 |
| XLOC_022187 | 17.42452493 | 0.367379694 | PAR | 9 | 16 | 0 | 5 | 28 | 0 |
| XLOC_022188 | 2.780267165 | -0.148314931 | PAR | 2 | 22 | 0 | 0 | 11 | 0 |
| XLOC_022189 | 7.959376489 | 0.312949804 | PAR | 0 | 7 | 0 | 2 | 6 | 0 |
| XLOC_022190 | 10.10329584 | 0.056581016 | PAR | 0 | 7 | 0 | 0 | 6 | 0 |
| XLOC_022191 | 18.3469457 | 0.219229852 | PAR | 6 | 0 | 0 | 2 | 0 | 0 |
| XLOC_022192 | 4.678497903 | 0.267947004 | PAR | 0 | 0 | 0 | 1 | 16 | 0 |
| XLOC_021100 | 778.5117042 | -0.118400577 | PAR | 11 | 9 | 0 | 14 | 21 | 0 |
| XLOC_021660 | 23.80073802 | -0.159797631 | PAR | 0 | 9 | 0 | 4 | 14 | 0 |
| XLOC_021101 | 5.254935227 | 0.380432448 | PAR | 1 | 4 | 10 | 0 | 9 | 0 |
| XLOC_021103 | 50.83734234 | 0.403246416 | PAR | 43 | 21 | 1 | 24 | 11 | 0 |
| XLOC_022193 | 49.26579846 | 0.254406225 | PAR | 1 | 0 | 0 | 8 | 11 | 0 |
| XLOC_021666 | 9.36456704 | 0.175897382 | PAR | 1 | 5 | 0 | 2 | 23 | 0 |
| XLOC_022194 | 3.087203575 | 0.925513598 | PAR | 4 | 1 | 0 | 5 | 17 | 0 |
| XLOC_022195 | 4.736362518 | 0.492947269 | PAR | 0 | 18 | 0 | 0 | 13 | 0 |
| XLOC_021109 | 2.147908603 | -0.251718704 | PAR | 0 | 2 | 0 | 5 | 20 | 0 |
| XLOC_021670 | 316.5208987 | 1.041882637 | PAR | 0 | 3 | 0 | 0 | 15 | 9 |
| XLOC_021112 | 241.4292808 | 0.054485254 | PAR | 1 | 7 | 8 | 5 | 19 | 11 |
| XLOC_022196 | 6.446682893 | 0.33876532 | PAR | 0 | 4 | 0 | 0 | 8 | 0 |
| XLOC_021113 | 279.7748465 | -0.010483661 | PAR | 0 | 0 | 0 | 3 | 2 | 10 |
| XLOC_021672 | 412.8090391 | 0.072902121 | PAR | 6 | 5 | 8 | 6 | 24 | 22 |
| XLOC_021673 | 14.06518675 | -0.100913461 | PAR | 0 | 0 | 0 | 2 | 14 | 3 |
| XLOC_022197 | 13.58586721 | 0.006172719 | PAR | 0 | 0 | 0 | 5 | 6 | 0 |
| XLOC_021674 | 165.8389003 | 0.041202929 | PAR | 0 | 0 | 0 | 1 | 10 | 0 |
| XLOC_022198 | 590.1518475 | -0.043784132 | PAR | 0 | 0 | 0 | 2 | 4 | 0 |
| XLOC_022199 | 36.98626066 | -0.117335624 | PAR | 4 | 3 | 0 | 3 | 10 | 0 |
| XLOC_021114 | 1593.966716 | -0.073640631 | PAR | 4 | 9 | 4 | 6 | 9 | 19 |
| XLOC_021675 | 553.7308108 | 0.04206423 | PAR | 2 | 8 | 7 | 0 | 11 | 16 |
| XLOC_021676 | 392.507478 | 0.006202895 | PAR | 2 | 14 | 13 | 2 | 14 | 27 |
| XLOC_022200 | 355.8955934 | -0.141871891 | PAR | 7 | 3 | 0 | 5 | 9 | 0 |
| XLOC_021677 | 1113.511414 | -0.029039763 | PAR | 1 | 9 | 1 | 85 | 70 | 120 |
| XLOC_021115 | 20.58216089 | -0.121444686 | PAR | 2 | 0 | 0 | 5 | 13 | 49 |
| XLOC_021678 | 747.8376146 | -0.128942585 | PAR | 0 | 0 | 2 | 23 | 36 | 57 |
| XLOC_021116 | 989.9766627 | 0.124851217 | PAR | 0 | 0 | 0 | 7 | 19 | 14 |
| XLOC_022201 | 12.356876 | -0.218125696 | PAR | 0 | 0 | 0 | 3 | 13 | 0 |
| XLOC_021679 | 270.8312126 | 0.027489686 | PAR | 3 | 5 | 1 | 15 | 7 | 6 |
| XLOC_021680 | 1091.38777 | 0.080914125 | PAR | 1 | 14 | 24 | 3 | 11 | 75 |
| XLOC_021681 | 15.6572207 | -0.116547411 | PAR | 0 | 6 | 0 | 0 | 14 | 0 |
| XLOC_022202 | 3.98262802 | 0.230888282 | PAR | 2 | 9 | 0 | 2 | 23 | 0 |
| XLOC_021682 | 1217.843127 | 0.064607635 | PAR | 3 | 13 | 3 | 4 | 27 | 2 |
| XLOC_021683 | 1252.604715 | -0.073504933 | PAR | 5 | 28 | 8 | 4 | 20 | 17 |
| XLOC_021117 | 1445.975759 | -0.003545587 | PAR | 3 | 6 | 16 | 3 | 17 | 2 |
| XLOC_021684 | 569.2108382 | 0.070741178 | PAR | 2 | 16 | 5 | 11 | 23 | 34 |
| XLOC_021685 | 139.3497779 | 0.184013989 | PAR | 0 | 10 | 25 | 8 | 16 | 22 |
| XLOC_021686 | 7260.462218 | 0.009895804 | PAR | 3 | 15 | 26 | 10 | 39 | 76 |
| XLOC_021687 | 62.97146205 | -0.052516877 | PAR | 0 | 4 | 0 | 11 | 24 | 4 |
| XLOC_022203 | 4.695682564 | 0.422234671 | PAR | 0 | 0 | 0 | 1 | 11 | 0 |
| XLOC_021688 | 1585.277717 | -0.037501444 | PAR | 6 | 16 | 68 | 37 | 26 | 110 |
| XLOC_022204 | 16933.63679 | 0.007021325 | PAR | 1 | 6 | 0 | 8 | 18 | 0 |
| XLOC_021689 | 4573.533603 | 0.096886914 | PAR | 1 | 16 | 70 | 3 | 38 | 144 |
| XLOC_022205 | 219.0333604 | 0.096236576 | PAR | 4 | 16 | 0 | 10 | 18 | 0 |
| XLOC_022206 | 22.68831285 | -0.177392687 | PAR | 0 | 18 | 0 | 2 | 10 | 0 |
| XLOC_021690 | 25.67339661 | 0.249098429 | PAR | 0 | 17 | 0 | 0 | 21 | 4 |
| XLOC_022207 | 504.1053073 | -0.016846352 | PAR | 0 | 0 | 0 | 8 | 11 | 0 |
| XLOC_021691 | 1172.898818 | 0.026047422 | PAR | 0 | 0 | 4 | 0 | 14 | 47 |
| XLOC_022208 | 8.99282233 | -0.168015032 | PAR | 0 | 0 | 0 | 0 | 15 | 0 |
| XLOC_022209 | 116.1770405 | 0.187695071 | PAR | 2 | 9 | 0 | 1 | 12 | 0 |
| XLOC_021692 | 416.5628597 | 0.13874511 | PAR | 5 | 8 | 4 | 10 | 9 | 5 |
| XLOC_021118 | 354.1909235 | 0.293407623 | PAR | 0 | 9 | 1 | 19 | 25 | 16 |
| XLOC_021119 | 133.7715729 | -0.119958727 | PAR | 1 | 1 | 4 | 2 | 18 | 7 |
| XLOC_022210 | 23.84950036 | 0.173406192 | PAR | 0 | 5 | 0 | 1 | 10 | 0 |
| XLOC_021693 | 382.5131305 | 0.319229509 | PAR | 2 | 4 | 18 | 8 | 12 | 52 |
| XLOC_021694 | 241.3467993 | -0.034121423 | PAR | 4 | 8 | 0 | 10 | 16 | 7 |
| XLOC_022211 | 25.19002306 | -0.596316919 | PAR | 3 | 1 | 0 | 9 | 7 | 0 |
| XLOC_021120 | 527.595627 | 0.397357886 | PAR | 1 | 9 | 1 | 6 | 19 | 30 |
| XLOC_021695 | 3329.514487 | -0.06826942 | PAR | 6 | 5 | 7 | 17 | 11 | 40 |
| XLOC_022212 | 25.77390517 | -0.082851866 | PAR | 0 | 0 | 0 | 3 | 8 | 0 |
| XLOC_021121 | 336.1708082 | -0.100395619 | PAR | 14 | 26 | 85 | 13 | 32 | 97 |
| XLOC_021696 | 707.4563667 | -0.025350402 | PAR | 6 | 9 | 17 | 7 | 18 | 16 |
| XLOC_022213 | 10.72771621 | -0.245264439 | PAR | 2 | 14 | 0 | 0 | 6 | 0 |
| XLOC_021697 | 5.166095081 | -0.430216816 | PAR | 0 | 9 | 0 | 1 | 4 | 27 |
| XLOC_022214 | 330.1844038 | -0.046400004 | PAR | 8 | 6 | 0 | 1 | 1 | 0 |
| XLOC_021698 | 41.20833241 | -0.084933269 | PAR | 7 | 22 | 11 | 0 | 3 | 1 |
| XLOC_021699 | 5.700310706 | -0.230583782 | PAR | 3 | 14 | 17 | 0 | 1 | 2 |
| XLOC_021122 | 254.909026 | 0.218242636 | PAR | 24 | 13 | 254 | 16 | 14 | 264 |
| XLOC_022215 | 6.321004333 | -0.138244151 | PAR | 1 | 19 | 0 | 1 | 7 | 0 |
| XLOC_022216 | 11.03925206 | 0.45302003 | PAR | 1 | 7 | 0 | 2 | 23 | 0 |
| XLOC_022217 | 10.94154984 | 0.057181209 | PAR | 5 | 6 | 0 | 0 | 5 | 0 |
| XLOC_021123 | 1243.304395 | -0.104046591 | PAR | 2 | 6 | 5 | 0 | 17 | 1 |
| XLOC_021125 | 655.906689 | -0.006210126 | PAR | 0 | 7 | 6 | 3 | 25 | 50 |
| XLOC_021701 | 942.6231391 | 0.020314374 | PAR | 1 | 3 | 22 | 1 | 9 | 10 |
| XLOC_021126 | 1111.355275 | 0.116685422 | PAR | 14 | 36 | 63 | 37 | 58 | 149 |
| XLOC_021702 | 291.366384 | -0.0952991 | PAR | 1 | 13 | 0 | 3 | 14 | 1 |
| XLOC_021703 | 184.0322554 | -0.68966647 | PAR | 5 | 13 | 14 | 7 | 8 | 10 |
| XLOC_022218 | 6.872950046 | -0.377809218 | PAR | 6 | 15 | 0 | 5 | 7 | 0 |
| XLOC_021704 | 2.528062336 | -0.110254381 | PAR | 1 | 14 | 12 | 5 | 21 | 19 |
| XLOC_021705 | 2602.296196 | -0.051598186 | PAR | 12 | 21 | 19 | 9 | 57 | 55 |
| XLOC_022219 | 24.25385071 | -0.198814191 | PAR | 1 | 17 | 0 | 5 | 11 | 0 |
| XLOC_022220 | 26.91500793 | -0.319219753 | PAR | 0 | 5 | 0 | 6 | 14 | 0 |
| XLOC_021706 | 439.6455529 | -0.059975629 | PAR | 8 | 7 | 16 | 10 | 19 | 17 |
| XLOC_022221 | 12.65247669 | -0.013014122 | PAR | 1 | 16 | 0 | 2 | 16 | 0 |
| XLOC_021127 | 26.98077965 | 0.218557203 | PAR | 3 | 1 | 32 | 8 | 41 | 101 |
| XLOC_022222 | 5.01997068 | 0.196538785 | PAR | 2 | 34 | 0 | 7 | 9 | 0 |
| XLOC_021707 | 535.5068952 | 0.10402914 | PAR | 4 | 19 | 14 | 5 | 14 | 20 |
| XLOC_022223 | 4.274635905 | 1.569750096 | PAR | 0 | 5 | 0 | 0 | 1 | 0 |
| XLOC_021128 | 160.5343508 | 0.027443447 | PAR | 1 | 4 | 53 | 11 | 9 | 144 |
| XLOC_021129 | 3365.135681 | 0.006315424 | PAR | 17 | 14 | 27 | 18 | 22 | 42 |
| XLOC_022224 | 74.00533107 | 0.126361328 | PAR | 0 | 8 | 0 | 0 | 13 | 0 |
| XLOC_021130 | 159.3169708 | 0.124488121 | PAR | 8 | 14 | 21 | 7 | 10 | 33 |
| XLOC_021131 | 1373.639893 | -0.019241872 | PAR | 5 | 26 | 36 | 6 | 32 | 28 |
| XLOC_022225 | 9.156349597 | 0.170512786 | PAR | 1 | 7 | 0 | 1 | 4 | 0 |
| XLOC_022226 | 11.75537618 | -0.161706896 | PAR | 1 | 8 | 0 | 1 | 3 | 0 |
| XLOC_022227 | 17.9724429 | 0.028068197 | PAR | 4 | 8 | 0 | 0 | 6 | 0 |
| XLOC_022228 | 49.26215115 | 0.532235746 | PAR | 7 | 18 | 0 | 9 | 12 | 0 |
| XLOC_022229 | 20.2074001 | 0.095709374 | PAR | 2 | 21 | 0 | 3 | 15 | 0 |
| XLOC_022230 | 18.04566567 | -0.207057035 | PAR | 0 | 13 | 0 | 1 | 17 | 0 |
| XLOC_022231 | 1979.140605 | -0.027019451 | PAR | 5 | 11 | 0 | 3 | 9 | 0 |
| XLOC_022232 | 119.293181 | -0.306766228 | PAR | 10 | 7 | 0 | 4 | 13 | 0 |
| XLOC_021708 | 2461.642204 | 0.017819552 | PAR | 2 | 10 | 16 | 8 | 29 | 36 |
| XLOC_021132 | 1194.398437 | 0.036545903 | PAR | 2 | 6 | 37 | 3 | 26 | 46 |
| XLOC_021709 | 511.0759723 | -0.078916843 | PAR | 1 | 11 | 30 | 21 | 12 | 35 |
| XLOC_022233 | 4.291236342 | -0.141786153 | PAR | 0 | 3 | 0 | 2 | 20 | 0 |
| XLOC_022234 | 6.438952851 | 0.216332243 | PAR | 3 | 0 | 0 | 7 | 11 | 0 |
| XLOC_021134 | 421.9519317 | 0.052261197 | PAR | 12 | 11 | 0 | 10 | 22 | 0 |
| XLOC_021710 | 165.3907848 | 0.010356761 | PAR | 0 | 11 | 3 | 2 | 26 | 29 |
| XLOC_021135 | 222.2798684 | -0.113107555 | PAR | 1 | 15 | 1 | 12 | 25 | 7 |
| XLOC_022235 | 5.933387359 | -0.789368339 | PAR | 10 | 12 | 0 | 0 | 15 | 0 |
| XLOC_022236 | 5.766111655 | -0.667444659 | PAR | 0 | 2 | 0 | 1 | 3 | 0 |
| XLOC_021711 | 557.7822969 | -0.122635323 | PAR | 8 | 8 | 76 | 7 | 13 | 34 |
| XLOC_022237 | 13.46074801 | -0.207862956 | PAR | 1 | 24 | 0 | 0 | 11 | 0 |
| XLOC_021712 | 2916.806108 | -0.101847576 | PAR | 3 | 20 | 25 | 5 | 25 | 57 |
| XLOC_022238 | 5.977592362 | 0.146803316 | PAR | 3 | 10 | 0 | 0 | 11 | 0 |
| XLOC_022239 | 4.818438 | 0.499096374 | PAR | 1 | 4 | 0 | 5 | 8 | 0 |
| XLOC_021136 | 68.95302143 | -0.055296112 | PAR | 2 | 6 | 41 | 3 | 5 | 48 |
| XLOC_022240 | 24.71258391 | 0.01912545 | PAR | 2 | 9 | 0 | 3 | 3 | 0 |
| XLOC_022241 | 7.734602987 | -0.024499511 | PAR | 6 | 9 | 0 | 10 | 15 | 0 |
| XLOC_022242 | 251.2267066 | -0.056253196 | PAR | 5 | 16 | 0 | 5 | 14 | 0 |
| XLOC_021713 | 30.58090379 | -0.203463606 | PAR | 1 | 3 | 44 | 9 | 16 | 100 |
| XLOC_021140 | 3.463050097 | -0.140384126 | PAR | 0 | 0 | 0 | 0 | 19 | 0 |
| XLOC_021715 | 7.284840791 | -0.035979312 | PAR | 0 | 7 | 0 | 1 | 25 | 0 |
| XLOC_021716 | 3.462919966 | -0.339526664 | PAR | 1 | 11 | 7 | 0 | 28 | 8 |
| XLOC_021143 | 291.189134 | 0.130843876 | PAR | 0 | 6 | 9 | 6 | 19 | 28 |
| XLOC_021717 | 2544.149861 | 0.092746305 | PAR | 1 | 14 | 20 | 16 | 20 | 82 |
| XLOC_021144 | 823.1715248 | 0.049592989 | PAR | 14 | 22 | 12 | 23 | 36 | 24 |
| XLOC_022243 | 18.83997212 | 0.445501269 | PAR | 0 | 9 | 0 | 3 | 16 | 0 |
| XLOC_021145 | 1569.136655 | 0.028810352 | PAR | 1 | 6 | 66 | 2 | 31 | 105 |
| XLOC_022244 | 28.45665708 | 0.220213923 | PAR | 0 | 5 | 0 | 2 | 2 | 0 |
| XLOC_022245 | 14.68158526 | 0.049755257 | PAR | 0 | 0 | 0 | 3 | 3 | 0 |
| XLOC_022246 | 117.0126768 | -0.459867037 | PAR | 6 | 2 | 0 | 6 | 8 | 0 |
| XLOC_022247 | 5.053855872 | 0.253732977 | PAR | 0 | 1 | 0 | 0 | 1 | 0 |
| XLOC_021146 | 185.5158521 | -0.046892158 | PAR | 5 | 10 | 5 | 8 | 8 | 7 |
| XLOC_022248 | 412.0656386 | -0.049741267 | PAR | 0 | 0 | 0 | 1 | 0 | 0 |
| XLOC_022249 | 4.716409701 | -0.259208725 | PAR | 0 | 0 | 0 | 4 | 12 | 0 |
| XLOC_022250 | 4.415510583 | 0.423534442 | PAR | 0 | 6 | 0 | 3 | 37 | 0 |
| XLOC_022251 | 9.983929801 | -0.471383176 | PAR | 0 | 3 | 0 | 26 | 22 | 0 |
| XLOC_021147 | 48.66386294 | 0.190596077 | PAR | 0 | 3 | 1 | 12 | 33 | 0 |
| XLOC_021148 | 453.4474033 | 0.009587843 | PAR | 2 | 16 | 20 | 4 | 23 | 27 |
| XLOC_021149 | 990.2104869 | -0.002027725 | PAR | 10 | 29 | 24 | 6 | 19 | 16 |
| XLOC_022252 | 5.977302374 | 0.068734387 | PAR | 0 | 19 | 0 | 0 | 9 | 0 |
| XLOC_021718 | 926.6886434 | 0.027711676 | PAR | 5 | 12 | 15 | 9 | 15 | 42 |
| XLOC_022253 | 19.05872267 | 0.123423101 | PAR | 5 | 11 | 0 | 1 | 22 | 0 |
| XLOC_021719 | 90.54210088 | 0.035741012 | PAR | 0 | 2 | 19 | 10 | 9 | 46 |
| XLOC_021720 | 1462.196216 | 0.142200309 | PAR | 4 | 1 | 12 | 5 | 26 | 47 |
| XLOC_021150 | 12.17041581 | -0.05925358 | PAR | 0 | 1 | 34 | 1 | 20 | 49 |
| XLOC_021151 | 225.9138477 | 0.217928977 | PAR | 0 | 7 | 6 | 1 | 10 | 14 |
| XLOC_022254 | 15.50011481 | -0.188083153 | PAR | 1 | 5 | 0 | 0 | 7 | 0 |
| XLOC_021721 | 4464.369589 | 0.012935198 | PAR | 6 | 2 | 4 | 11 | 9 | 15 |
| XLOC_021152 | 278.1714108 | 0.101738121 | PAR | 1 | 8 | 2 | 0 | 7 | 2 |
| XLOC_021158 | 2.158578032 | -0.063213045 | PAR | 0 | 13 | 0 | 0 | 32 | 0 |
| XLOC_021162 | 2.703613518 | 0.170466935 | PAR | 0 | 5 | 0 | 0 | 25 | 0 |
| XLOC_021725 | 1446.439616 | 0.027658685 | PAR | 0 | 1 | 1 | 2 | 2 | 32 |
| XLOC_021170 | 35.32309801 | 0.347879686 | PAR | 1 | 1 | 0 | 1 | 2 | 0 |
| XLOC_021171 | 716.9804983 | 0.042307757 | PAR | 0 | 6 | 0 | 0 | 3 | 6 |
| XLOC_022255 | 9.124570791 | -0.335924872 | PAR | 4 | 9 | 0 | 3 | 6 | 0 |
| XLOC_021733 | 3155.008586 | -0.109956827 | PAR | 10 | 17 | 33 | 6 | 13 | 68 |
| XLOC_021734 | 111.0154884 | 0.166047572 | PAR | 0 | 5 | 9 | 2 | 3 | 14 |
| XLOC_021172 | 173.3705374 | -1.244252424 | PAR | 11 | 25 | 16 | 30 | 23 | 43 |
| XLOC_021582 | 465.632945 | -0.89266465 | PAR | 0 | 3 | 0 | 0 | 2 | 0 |
| XLOC_021173 | 33.89699349 | 0.545222115 | stratum I | 8 | 37 | 26 | 14 | 76 | 37 |
| XLOC_021174 | 182.5148551 | -0.615218127 | stratum I | 7 | 11 | 15 | 12 | 11 | 25 |
| XLOC_021175 | 11.80300913 | -0.480115022 | stratum I | 0 | 3 | 2 | 0 | 2 | 1 |
| XLOC_022256 | 6.084950972 | -0.542685322 | stratum I | 2 | 7 | 0 | 7 | 20 | 0 |
| XLOC_021176 | 14.0863736 | -0.586084944 | stratum I | 0 | 18 | 0 | 0 | 20 | 0 |
| XLOC_021177 | 7.56278422 | 0.350635119 | stratum I | 0 | 0 | 0 | 0 | 0 | 0 |
| XLOC_022257 | 3.293073446 | -0.488068571 | stratum I | 2 | 22 | 0 | 12 | 34 | 0 |
| XLOC_022258 | 2.457501084 | 0.335260948 | stratum I | 0 | 6 | 0 | 0 | 3 | 0 |
| XLOC_022259 | 2.366380462 | 0.452523751 | stratum I | 0 | 2 | 0 | 0 | 4 | 0 |
| XLOC_021178 | 5.113907409 | 0.801319548 | stratum I | 1 | 8 | 0 | 0 | 19 | 0 |
| XLOC_022260 | 5.813019523 | 1.952932485 | stratum I | 7 | 18 | 0 | 14 | 40 | 0 |
| XLOC_021736 | 1229.093344 | -0.573707034 | stratum I | 8 | 20 | 92 | 17 | 56 | 235 |
| XLOC_021737 | 919.6616127 | -1.002815753 | stratum I | 21 | 22 | 26 | 31 | 64 | 73 |
| XLOC_022262 | 7.371625626 | 2.158824122 | stratum I | 0 | 0 | 0 | 0 | 9 | 0 |
| XLOC_021179 | 137.2447034 | 0.572562599 | stratum I | 4 | 30 | 380 | 21 | 108 | 1075 |
| XLOC_022263 | 11.06370447 | -0.314286263 | stratum I | 14 | 24 | 0 | 14 | 81 | 0 |
| XLOC_022264 | 40.76036882 | -0.108940084 | stratum I | 7 | 39 | 0 | 34 | 71 | 0 |
| XLOC_022265 | 10.86587328 | 0.131023903 | stratum I | 2 | 25 | 0 | 7 | 61 | 0 |
| XLOC_022266 | 11.61127023 | -0.501381954 | stratum I | 0 | 30 | 0 | 0 | 67 | 0 |
| XLOC_022267 | 2875.65951 | -0.521783792 | stratum I | 14 | 7 | 0 | 44 | 27 | 0 |
| XLOC_021738 | 112.9796875 | -0.793326425 | stratum I | 10 | 12 | 14 | 23 | 44 | 28 |
| XLOC_022268 | 9.783805998 | 2.329489986 | stratum I | 0 | 10 | 0 | 0 | 42 | 0 |
| XLOC_022269 | 297.3640692 | -0.864413705 | stratum I | 2 | 25 | 0 | 16 | 31 | 0 |
| XLOC_021739 | 540.3591292 | -0.282006568 | stratum I | 19 | 7 | 39 | 17 | 33 | 100 |
| XLOC_021740 | 33.63718513 | -0.833086394 | stratum I | 1 | 40 | 9 | 1 | 48 | 23 |
| XLOC_021741 | 92.4113697 | -0.695973134 | stratum I | 20 | 29 | 2 | 46 | 40 | 11 |
| XLOC_021742 | 16.03759118 | 0.413323207 | stratum I | 6 | 23 | 8 | 0 | 47 | 31 |
| XLOC_022270 | 9.043882516 | -0.555493229 | stratum I | 0 | 18 | 0 | 1 | 20 | 0 |
| XLOC_022271 | 175.1589918 | -1.071309524 | stratum I | 13 | 17 | 0 | 37 | 43 | 0 |
| XLOC_021743 | 303.2523566 | -0.602813065 | stratum I | 21 | 41 | 221 | 32 | 107 | 577 |
| XLOC_021744 | 846.7039476 | -0.29351034 | stratum I | 44 | 7 | 88 | 95 | 38 | 315 |
| XLOC_021745 | 1030.297353 | -0.356223587 | stratum I | 22 | 0 | 22 | 59 | 2 | 53 |
| XLOC_021746 | 63.18464259 | -0.985539385 | stratum I | 9 | 33 | 10 | 23 | 106 | 44 |
| XLOC_021180 | 1427.390493 | -0.329409396 | stratum I | 31 | 50 | 96 | 109 | 120 | 314 |
| XLOC_022272 | 6.093711152 | -0.066143708 | stratum I | 3 | 7 | 0 | 7 | 49 | 0 |
| XLOC_022273 | 7.949490517 | -0.183267642 | stratum I | 0 | 13 | 0 | 0 | 55 | 0 |
| XLOC_022274 | 19.83114103 | 0.103349986 | stratum I | 11 | 26 | 0 | 32 | 31 | 0 |
| XLOC_021747 | 378.6610208 | -0.600815235 | stratum I | 20 | 15 | 0 | 20 | 45 | 0 |
| XLOC_022275 | 87.79058284 | -0.539118201 | stratum I | 2 | 26 | 0 | 10 | 48 | 0 |
| XLOC_022276 | 10.22413693 | -0.81967477 | stratum I | 2 | 5 | 0 | 13 | 39 | 0 |
| XLOC_022277 | 19.26844567 | -0.834004477 | stratum I | 3 | 13 | 0 | 22 | 41 | 0 |
| XLOC_022278 | 6.112641769 | -0.269164469 | stratum I | 3 | 29 | 0 | 11 | 56 | 0 |
| XLOC_022279 | 4.267291582 | 0.663176393 | stratum I | 0 | 19 | 0 | 0 | 28 | 0 |
| XLOC_021748 | 27.78120382 | -0.09783986 | stratum I | 0 | 20 | 0 | 3 | 43 | 2 |
| XLOC_021181 | 170.5293417 | -0.979955512 | stratum I | 9 | 27 | 19 | 33 | 63 | 49 |
| XLOC_021182 | 4355.400988 | -0.755986331 | stratum I | 12 | 40 | 20 | 29 | 80 | 47 |
| XLOC_022280 | 113.9311608 | 0.512213203 | stratum I | 31 | 19 | 0 | 80 | 54 | 0 |
| XLOC_021749 | 20.97831212 | 1.1444724 | stratum I | 12 | 34 | 72 | 32 | 102 | 218 |
| XLOC_021750 | 1184.126434 | 0.0000626 | stratum I | 28 | 43 | 167 | 46 | 92 | 511 |
| XLOC_022281 | 25.70314206 | -0.634506235 | stratum I | 1 | 25 | 0 | 6 | 76 | 0 |
| XLOC_022282 | 40.2939936 | -0.730996326 | stratum I | 4 | 15 | 0 | 16 | 64 | 0 |
| XLOC_021751 | 392.5537511 | -0.87149638 | stratum I | 11 | 31 | 16 | 33 | 55 | 72 |
| XLOC_022283 | 2457.371522 | -0.524049092 | stratum I | 6 | 22 | 0 | 34 | 73 | 0 |
| XLOC_021752 | 406.8364234 | 0.149713452 | stratum I | 14 | 2 | 37 | 29 | 37 | 115 |
| XLOC_022284 | 6.676550832 | -0.381741402 | stratum I | 0 | 25 | 0 | 17 | 32 | 0 |
| XLOC_021753 | 1558.01142 | -0.648742314 | stratum I | 15 | 61 | 57 | 39 | 98 | 80 |
| XLOC_021754 | 646.8309489 | 0.030921168 | stratum I | 19 | 19 | 36 | 32 | 46 | 108 |
| XLOC_021183 | 1439.758529 | -0.462107464 | stratum I | 34 | 40 | 83 | 54 | 107 | 195 |
| XLOC_021755 | 159.0526617 | -1.040059139 | stratum I | 8 | 9 | 35 | 10 | 11 | 116 |
| XLOC_021184 | 288.7762311 | -0.561619426 | stratum I | 46 | 6 | 2 | 125 | 11 | 17 |
| XLOC_021185 | 1196.37099 | -0.031569131 | stratum I | 22 | 28 | 19 | 66 | 91 | 48 |
| XLOC_021756 | 39.48281094 | -0.632098399 | stratum I | 23 | 27 | 20 | 21 | 100 | 33 |
| XLOC_022285 | 3.876575604 | -0.516517401 | stratum I | 0 | 23 | 0 | 4 | 62 | 0 |
| XLOC_021186 | 87.87798468 | -0.835711265 | stratum I | 4 | 22 | 134 | 13 | 104 | 287 |
| XLOC_021187 | 230.5491037 | -0.966596576 | stratum I | 15 | 0 | 0 | 20 | 0 | 14 |
| XLOC_021757 | 796.5906751 | -0.460794755 | stratum I | 25 | 0 | 38 | 70 | 0 | 103 |
| XLOC_021758 | 20.45602184 | 0.620314001 | stratum I | 0 | 10 | 67 | 0 | 79 | 185 |
| XLOC_021759 | 37.18062717 | -0.118598226 | stratum I | 8 | 25 | 26 | 40 | 74 | 60 |
| XLOC_022288 | 2.90418523 | 0.420218204 | stratum I | 0 | 24 | 0 | 5 | 96 | 0 |
| XLOC_021760 | 2.074475272 | 0.465283754 | stratum I | 3 | 32 | 83 | 10 | 95 | 191 |
| XLOC_021761 | 4.920491619 | -0.38574667 | stratum I | 10 | 25 | 1 | 15 | 59 | 21 |
| XLOC_021188 | 14.44157637 | 2.752513515 | stratum I | 14 | 21 | 52 | 28 | 61 | 80 |
| XLOC_021189 | 1908.821473 | -0.910780118 | stratum I | 23 | 27 | 17 | 69 | 48 | 32 |
| XLOC_021190 | 588.5596771 | -0.389318109 | stratum I | 43 | 43 | 87 | 89 | 117 | 256 |
| XLOC_022289 | 12.34360512 | -0.572271967 | stratum I | 1 | 31 | 0 | 15 | 69 | 0 |
| XLOC_021191 | 3388.512229 | -0.017031368 | stratum I | 48 | 43 | 522 | 140 | 124 | 1196 |
| XLOC_022290 | 21.98466604 | -0.356383069 | stratum I | 2 | 43 | 0 | 10 | 80 | 0 |
| XLOC_021192 | 2094.025258 | -0.694561646 | stratum I | 26 | 31 | 307 | 48 | 64 | 861 |
| XLOC_022291 | 4.131560611 | -0.063883052 | stratum I | 10 | 27 | 0 | 13 | 64 | 0 |
| XLOC_021762 | 841.5266507 | 0.081754503 | stratum I | 55 | 57 | 180 | 111 | 112 | 431 |
| XLOC_021193 | 20.12950166 | -1.0336117 | stratum I | 0 | 43 | 0 | 0 | 87 | 10 |
| XLOC_022292 | 3.368312619 | 1.466380364 | stratum I | 0 | 14 | 0 | 0 | 23 | 0 |
| XLOC_021194 | 160.768977 | -1.26378231 | stratum I | 0 | 9 | 0 | 0 | 18 | 0 |
| XLOC_021195 | 33.30704827 | -1.115525419 | stratum I | 1 | 14 | 52 | 5 | 59 | 136 |
| XLOC_022293 | 20.1215991 | 2.141721957 | stratum I | 0 | 0 | 0 | 0 | 0 | 0 |
| XLOC_022294 | 37.27443978 | -0.153283548 | stratum I | 34 | 21 | 0 | 53 | 57 | 0 |
| XLOC_021763 | 349.9418056 | -1.135187292 | stratum I | 22 | 23 | 1 | 47 | 75 | 3 |
| XLOC_022295 | 12.29982045 | -0.617283435 | stratum I | 2 | 10 | 0 | 14 | 36 | 0 |
| XLOC_021764 | 13.02670374 | -0.66864783 | stratum I | 0 | 30 | 3 | 1 | 72 | 4 |
| XLOC_021765 | 310.9082924 | -0.134641395 | stratum I | 41 | 22 | 102 | 73 | 42 | 255 |
| XLOC_021766 | 381.5956613 | -0.620809876 | stratum I | 16 | 8 | 71 | 69 | 49 | 162 |
| XLOC_021767 | 70.52203535 | -0.808336442 | stratum I | 16 | 29 | 22 | 54 | 97 | 79 |
| XLOC_021768 | 89.98522324 | -0.736436282 | stratum I | 7 | 16 | 5 | 42 | 45 | 26 |
| XLOC_022296 | 4.562875676 | 1.403713669 | stratum I | 0 | 0 | 0 | 1 | 3 | 0 |
| XLOC_021769 | 19.4197619 | -0.724663461 | stratum I | 24 | 13 | 94 | 45 | 42 | 205 |
| XLOC_021196 | 246.3146886 | -0.885868071 | stratum I | 25 | 23 | 42 | 65 | 67 | 118 |
| XLOC_022297 | 17.59931092 | -0.851152336 | stratum I | 3 | 36 | 0 | 2 | 88 | 0 |
| XLOC_022298 | 4.728049131 | -0.54900573 | stratum I | 3 | 27 | 0 | 14 | 56 | 0 |
| XLOC_021198 | 48.69373994 | -0.924914716 | stratum I | 30 | 22 | 21 | 49 | 72 | 55 |
| XLOC_022299 | 32.43368241 | -0.82557417 | stratum I | 0 | 29 | 0 | 11 | 62 | 0 |
| XLOC_021770 | 4741.468959 | -0.269569756 | stratum I | 40 | 53 | 164 | 107 | 115 | 429 |
| XLOC_021199 | 848.2856836 | -0.907936548 | stratum I | 16 | 16 | 22 | 41 | 29 | 62 |
| XLOC_021771 | 4173.920537 | -0.565786637 | stratum I | 42 | 27 | 125 | 101 | 111 | 309 |
| XLOC_022300 | 27.51683389 | -0.854977313 | stratum I | 15 | 27 | 0 | 19 | 56 | 0 |
| XLOC_022301 | 12.55302687 | -0.684184967 | stratum I | 0 | 21 | 0 | 0 | 87 | 0 |
| XLOC_021200 | 507.3193312 | -1.022258639 | stratum I | 9 | 11 | 88 | 53 | 77 | 237 |
| XLOC_022302 | 20.61626191 | -0.932062979 | stratum I | 0 | 1 | 0 | 1 | 11 | 0 |
| XLOC_022303 | 22.62508803 | -0.924860291 | stratum I | 0 | 11 | 0 | 0 | 27 | 0 |
| XLOC_022304 | 22.32182623 | -0.709938049 | stratum I | 0 | 17 | 0 | 1 | 39 | 0 |
| XLOC_022305 | 15.87275196 | -0.624934958 | stratum I | 3 | 25 | 0 | 2 | 46 | 0 |
| XLOC_022306 | 25.6575544 | -0.775435111 | stratum I | 1 | 28 | 0 | 6 | 72 | 0 |
| XLOC_021201 | 54.69997739 | -0.330721133 | stratum I | 34 | 21 | 32 | 80 | 60 | 77 |
| XLOC_021772 | 111.0073808 | -1.053454906 | stratum I | 28 | 37 | 2 | 54 | 65 | 3 |
| XLOC_021202 | 264.6281795 | -0.186128329 | stratum I | 30 | 21 | 49 | 70 | 38 | 93 |
| XLOC_021203 | 26.97513012 | -0.911297045 | stratum I | 33 | 49 | 14 | 46 | 92 | 29 |
| XLOC_022307 | 16.95011457 | -0.591211215 | stratum I | 2 | 51 | 0 | 5 | 83 | 0 |
| XLOC_021773 | 1527.984458 | -0.041918797 | stratum I | 29 | 8 | 133 | 52 | 25 | 330 |
| XLOC_022308 | 12.40850787 | -0.93476968 | stratum I | 0 | 34 | 0 | 5 | 70 | 0 |
| XLOC_022309 | 10.52058392 | 1.654248177 | stratum I | 0 | 0 | 0 | 0 | 0 | 0 |
| XLOC_021206 | 311.8252029 | -1.258488695 | stratum I | 14 | 8 | 0 | 34 | 7 | 3 |
| XLOC_022310 | 9.457417317 | 2.646982203 | stratum I | 0 | 22 | 0 | 0 | 43 | 0 |
| XLOC_022311 | 14.56119302 | -0.317036335 | stratum I | 13 | 31 | 0 | 35 | 57 | 0 |
| XLOC_021208 | 7653.466871 | -0.359724251 | stratum I | 37 | 33 | 412 | 82 | 66 | 1036 |
| XLOC_021209 | 207.3026991 | -0.351154634 | stratum I | 38 | 7 | 135 | 91 | 40 | 314 |
| XLOC_021210 | 2063.63439 | -0.225902708 | stratum I | 82 | 45 | 63 | 169 | 111 | 160 |
| XLOC_021775 | 151.9393251 | 1.155992131 | stratum I | 10 | 20 | 15 | 13 | 51 | 35 |
| XLOC_022312 | 10.50264408 | 0.222936496 | stratum I | 7 | 24 | 0 | 12 | 65 | 0 |
| XLOC_021776 | 546.1070667 | -0.678098443 | stratum I | 16 | 26 | 28 | 33 | 54 | 85 |
| XLOC_021777 | 2.86115394 | -0.431430733 | stratum I | 0 | 8 | 0 | 0 | 25 | 0 |
| XLOC_021778 | 200.4807026 | -0.998639463 | stratum I | 7 | 7 | 0 | 12 | 17 | 6 |
| XLOC_022313 | 9.867749605 | -0.448620289 | stratum I | 15 | 0 | 0 | 14 | 11 | 0 |
| XLOC_021212 | 5.772937375 | -0.645960107 | stratum I | 0 | 0 | 0 | 0 | 21 | 0 |
| XLOC_022314 | 7.108500433 | -0.855573932 | stratum I | 0 | 0 | 0 | 0 | 0 | 0 |
| XLOC_021780 | 4.137063206 | -0.303539399 | stratum I | 0 | 6 | 0 | 1 | 6 | 0 |
| XLOC_021216 | 9.316923135 | 0.06819281 | stratum I | 2 | 21 | 28 | 12 | 85 | 100 |
| XLOC_021782 | 85.57290507 | -0.860962726 | stratum I | 12 | 7 | 7 | 29 | 36 | 9 |
| XLOC_021783 | 792.5254677 | -0.595690006 | stratum I | 15 | 18 | 27 | 43 | 23 | 89 |
| XLOC_021784 | 102.2641839 | -0.500466132 | stratum I | 12 | 26 | 6 | 12 | 48 | 4 |
| XLOC_021785 | 6.59500631 | -0.375349069 | stratum I | 6 | 21 | 6 | 15 | 32 | 12 |
| XLOC_021218 | 6748.994251 | -0.808605154 | stratum I | 0 | 3 | 0 | 1 | 11 | 5 |
| XLOC_021219 | 317.889571 | -0.754046802 | stratum I | 0 | 0 | 0 | 0 | 6 | 0 |
| XLOC_022315 | 29.39370338 | -0.411416236 | stratum I | 0 | 22 | 0 | 6 | 51 | 0 |
| XLOC_021220 | 324.7818983 | -0.253593984 | stratum I | 21 | 32 | 132 | 59 | 108 | 354 |
| XLOC_022316 | 10.59975196 | -0.111148007 | stratum I | 2 | 17 | 0 | 20 | 37 | 0 |
| XLOC_021221 | 93.90676464 | -0.668861811 | stratum I | 18 | 24 | 12 | 33 | 47 | 28 |
| XLOC_021222 | 1091.909123 | -0.339471279 | stratum I | 27 | 27 | 66 | 66 | 117 | 150 |
| XLOC_022317 | 6.393621935 | 1.518020348 | stratum I | 2 | 37 | 0 | 3 | 88 | 0 |
| XLOC_021224 | 1230.134041 | -1.023916376 | stratum I | 58 | 65 | 40 | 102 | 159 | 95 |
| XLOC_021225 | 161.4754216 | 0.197940452 | stratum I | 25 | 48 | 16 | 63 | 108 | 42 |
| XLOC_022318 | 25.4254606 | 0.13445229 | stratum I | 4 | 18 | 0 | 9 | 46 | 0 |
| XLOC_021787 | 142.1054069 | 0.39628285 | stratum I | 37 | 39 | 62 | 87 | 108 | 148 |
| XLOC_022319 | 7.248714379 | -0.71106128 | stratum I | 0 | 12 | 0 | 4 | 45 | 0 |
| XLOC_021788 | 285.6949855 | 0.096255772 | stratum I | 29 | 60 | 36 | 70 | 129 | 96 |
| XLOC_021226 | 1581.094426 | -0.874166646 | stratum I | 19 | 28 | 45 | 52 | 73 | 119 |
| XLOC_021789 | 4.828238608 | -0.131871567 | stratum I | 2 | 17 | 44 | 3 | 39 | 111 |
| XLOC_021227 | 250.9365861 | -0.31033812 | stratum I | 35 | 25 | 29 | 76 | 45 | 62 |
| XLOC_021228 | 930.591469 | -0.831280823 | stratum I | 42 | 28 | 19 | 79 | 76 | 65 |
| XLOC_021229 | 72.99255622 | 1.940094126 | stratum I | 24 | 29 | 2 | 79 | 74 | 6 |
| XLOC_021790 | 37.32424716 | -1.100384775 | stratum I | 21 | 32 | 17 | 56 | 86 | 57 |
| XLOC_022320 | 736.4368022 | -0.473503299 | stratum I | 31 | 16 | 0 | 64 | 36 | 0 |
| XLOC_021791 | 213.0894488 | -0.698943136 | stratum I | 7 | 44 | 241 | 5 | 108 | 655 |
| XLOC_022321 | 3.824956223 | -0.4139429 | stratum I | 0 | 16 | 0 | 5 | 65 | 0 |
| XLOC_022322 | 12.22863879 | -0.219253314 | stratum I | 13 | 22 | 0 | 26 | 58 | 0 |
| XLOC_021230 | 1497.616882 | -0.595111991 | stratum I | 18 | 75 | 1324 | 54 | 188 | 3156 |
| XLOC_022323 | 17.97529189 | -0.403004151 | stratum I | 16 | 23 | 0 | 36 | 62 | 0 |
| XLOC_022324 | 37.35225146 | -0.678722367 | stratum I | 11 | 9 | 0 | 36 | 41 | 0 |
| XLOC_021231 | 41.93909553 | -0.339872818 | stratum I | 0 | 26 | 270 | 7 | 86 | 611 |
| XLOC_022325 | 6.937376262 | -0.075132085 | stratum I | 0 | 9 | 0 | 0 | 33 | 0 |
| XLOC_021792 | 285.1198398 | -0.456557324 | stratum I | 15 | 27 | 36 | 27 | 70 | 99 |
| XLOC_021232 | 11.90925313 | -1.355912961 | stratum I | 11 | 25 | 15 | 11 | 63 | 39 |
| XLOC_021233 | 181.3040641 | -1.154311714 | stratum I | 15 | 39 | 54 | 41 | 111 | 208 |
| XLOC_022326 | 10.44873931 | -0.287899331 | stratum I | 8 | 39 | 0 | 23 | 84 | 0 |
| XLOC_022327 | 13.0227527 | -0.393484726 | stratum I | 3 | 14 | 0 | 36 | 70 | 0 |
| XLOC_021793 | 1858.663452 | -0.645149237 | stratum I | 8 | 4 | 4 | 22 | 37 | 19 |
| XLOC_021234 | 3.529708442 | -0.518270389 | stratum I | 1 | 11 | 108 | 4 | 49 | 340 |
| XLOC_022328 | 33.41282519 | -1.0940852 | stratum I | 19 | 11 | 0 | 51 | 40 | 0 |
| XLOC_021235 | 306.1392771 | -0.101124715 | stratum I | 17 | 34 | 105 | 28 | 59 | 278 |
| XLOC_022329 | 48.97168217 | -0.710444079 | stratum I | 3 | 21 | 0 | 16 | 53 | 0 |
| XLOC_021236 | 2.024208548 | 0.478230509 | stratum I | 2 | 37 | 33 | 26 | 81 | 58 |
| XLOC_021237 | 444.1036131 | -0.794492502 | stratum I | 27 | 26 | 54 | 30 | 64 | 85 |
| XLOC_021238 | 365.2935475 | -0.470787996 | stratum I | 23 | 20 | 64 | 48 | 82 | 141 |
| XLOC_022330 | 126.9667118 | -1.093208892 | stratum I | 1 | 28 | 0 | 6 | 72 | 0 |
| XLOC_021239 | 3419.26504 | -0.009218607 | stratum I | 6 | 30 | 27 | 16 | 62 | 69 |
| XLOC_021240 | 8.434755566 | -0.718080482 | stratum I | 3 | 18 | 2 | 15 | 47 | 9 |
| XLOC_021241 | 48.48569587 | -0.892436435 | stratum I | 16 | 17 | 1 | 35 | 47 | 2 |
| XLOC_021794 | 302.5337069 | -0.588846366 | stratum I | 48 | 16 | 34 | 110 | 59 | 105 |
| XLOC_021795 | 13.74918533 | -0.033253303 | stratum I | 5 | 18 | 35 | 9 | 46 | 67 |
| XLOC_021242 | 164.730203 | 0.18476179 | stratum I | 22 | 18 | 30 | 40 | 65 | 66 |
| XLOC_021796 | 38.41422508 | -0.698769518 | stratum I | 15 | 30 | 24 | 21 | 57 | 38 |
| XLOC_021243 | 279.8033583 | -0.479026143 | stratum I | 3 | 39 | 39 | 20 | 77 | 94 |
| XLOC_021797 | 100.780312 | -0.858355662 | stratum I | 4 | 20 | 6 | 17 | 43 | 8 |
| XLOC_021798 | 187.294319 | -0.738042048 | stratum I | 10 | 13 | 3 | 15 | 39 | 10 |
| XLOC_021244 | 313.2887659 | -0.349419242 | stratum I | 9 | 14 | 12 | 36 | 23 | 41 |
| XLOC_022331 | 462.9684785 | -0.943611376 | stratum I | 7 | 16 | 0 | 9 | 49 | 0 |
| XLOC_021799 | 510.0505177 | -0.063565052 | stratum I | 13 | 21 | 9 | 24 | 56 | 41 |
| XLOC_021245 | 664.7386325 | -0.295395629 | stratum I | 42 | 31 | 53 | 81 | 83 | 126 |
| XLOC_021800 | 1231.104674 | -0.367062986 | stratum I | 7 | 32 | 106 | 26 | 115 | 310 |
| XLOC_022332 | 8.862540979 | -0.375220503 | stratum I | 6 | 15 | 0 | 10 | 37 | 0 |
| XLOC_022333 | 1018.796354 | -0.241844205 | stratum I | 8 | 6 | 0 | 17 | 19 | 0 |
| XLOC_021801 | 411.2319382 | 0.554719001 | stratum I | 10 | 11 | 117 | 20 | 31 | 252 |
| XLOC_022334 | 6.363311507 | -0.580604242 | stratum I | 0 | 10 | 0 | 0 | 17 | 0 |
| XLOC_021246 | 41.72380607 | -0.472918836 | stratum I | 3 | 37 | 31 | 29 | 78 | 84 |
| XLOC_021802 | 153.9618728 | -0.937045778 | stratum I | 9 | 32 | 20 | 24 | 53 | 29 |
| XLOC_022336 | 5.189299201 | 0.288117559 | stratum I | 9 | 14 | 0 | 12 | 30 | 0 |
| XLOC_022337 | 3.916569597 | -0.001251363 | stratum I | 6 | 17 | 0 | 13 | 22 | 0 |
| XLOC_021803 | 421.6750684 | -0.595225992 | stratum I | 35 | 9 | 239 | 48 | 18 | 636 |
| XLOC_022338 | 9.817222877 | -0.283558705 | stratum I | 0 | 9 | 0 | 8 | 36 | 0 |
| XLOC_022339 | 10.56634709 | -0.302719029 | stratum I | 0 | 26 | 0 | 0 | 51 | 0 |
| XLOC_021247 | 440.7965698 | -0.905023408 | stratum I | 19 | 10 | 22 | 41 | 30 | 73 |
| XLOC_021804 | 73.19498763 | -0.939213633 | stratum I | 2 | 8 | 30 | 12 | 18 | 76 |
| XLOC_021249 | 1279.529166 | -0.60573766 | stratum I | 22 | 19 | 477 | 45 | 45 | 1276 |
| XLOC_022340 | 8.785372485 | -0.577000002 | stratum I | 0 | 0 | 0 | 0 | 13 | 0 |
| XLOC_022341 | 10.46980675 | -0.412267974 | stratum I | 7 | 13 | 0 | 19 | 54 | 0 |
| XLOC_022342 | 19.9365701 | -0.659914061 | stratum I | 4 | 31 | 0 | 6 | 63 | 0 |
| XLOC_021251 | 6.483026139 | 0.985519233 | stratum I | 1 | 6 | 32 | 2 | 29 | 114 |
| XLOC_022343 | 2.704868519 | 0.783912734 | stratum I | 5 | 10 | 0 | 5 | 47 | 0 |
| XLOC_021805 | 225.2566131 | -0.64025029 | stratum I | 6 | 19 | 20 | 20 | 42 | 63 |
| XLOC_022344 | 3.728818169 | 1.476832741 | stratum I | 3 | 10 | 0 | 3 | 45 | 0 |
| XLOC_022345 | 1041.403864 | -0.161229823 | stratum I | 5 | 19 | 0 | 16 | 37 | 0 |
| XLOC_021806 | 509.9116345 | -0.020599053 | stratum I | 5 | 24 | 41 | 7 | 65 | 78 |
| XLOC_022346 | 19.74044545 | -0.566803684 | stratum I | 2 | 12 | 0 | 5 | 38 | 0 |
| XLOC_021807 | 477.4411929 | -0.118436403 | stratum I | 24 | 32 | 117 | 51 | 90 | 251 |
| XLOC_021808 | 827.4764157 | 0.414374569 | stratum I | 38 | 58 | 120 | 80 | 146 | 267 |
| XLOC_021809 | 2223.045596 | -0.277520859 | stratum I | 16 | 39 | 179 | 38 | 74 | 329 |
| XLOC_022347 | 11.13330367 | -0.17884664 | stratum I | 0 | 13 | 0 | 3 | 21 | 0 |
| XLOC_022348 | 9.679275474 | -0.844663207 | stratum I | 2 | 9 | 0 | 4 | 22 | 0 |
| XLOC_022349 | 8.898374644 | -0.185291114 | stratum I | 1 | 11 | 0 | 4 | 21 | 0 |
| XLOC_021810 | 5100.70637 | -0.006166139 | stratum I | 16 | 26 | 19 | 38 | 66 | 71 |
| XLOC_022350 | 1245.060653 | -0.021758263 | stratum I | 25 | 25 | 0 | 16 | 51 | 0 |
| XLOC_021811 | 2.227083017 | -0.053403996 | stratum I | 5 | 30 | 0 | 9 | 39 | 0 |
| XLOC_021252 | 404.7221347 | 0.324039924 | stratum I | 9 | 24 | 28 | 31 | 37 | 52 |
| XLOC_021812 | 775.0742277 | -0.283149934 | stratum I | 12 | 13 | 45 | 45 | 29 | 94 |
| XLOC_021253 | 5.04500141 | -0.578125923 | stratum I | 0 | 2 | 7 | 1 | 10 | 10 |
| XLOC_022351 | 2.295634046 | 1.040816526 | stratum I | 1 | 1 | 0 | 3 | 14 | 0 |
| XLOC_022352 | 4.176702688 | -0.66123555 | stratum I | 0 | 5 | 0 | 0 | 17 | 0 |
| XLOC_022353 | 4.866558625 | 0.0829467 | stratum I | 3 | 7 | 0 | 3 | 27 | 0 |
| XLOC_021254 | 51.32624752 | -1.078161148 | stratum I | 22 | 11 | 0 | 64 | 38 | 4 |
| XLOC_021813 | 360.0656267 | -1.100819672 | stratum I | 13 | 9 | 5 | 39 | 21 | 15 |
| XLOC_021814 | 179.2984731 | -0.884606888 | stratum I | 18 | 16 | 27 | 41 | 67 | 90 |
| XLOC_022354 | 2.554337302 | 0.919916462 | stratum I | 1 | 19 | 0 | 0 | 74 | 0 |
| XLOC_021255 | 185.2333145 | -0.380583872 | stratum I | 25 | 12 | 5 | 43 | 72 | 21 |
| XLOC_022355 | 28.81999006 | -0.341849191 | stratum I | 0 | 29 | 0 | 19 | 77 | 0 |
| XLOC_021815 | 195.7086684 | -0.848562241 | stratum I | 1 | 24 | 11 | 12 | 63 | 37 |
| XLOC_021816 | 2266.986697 | -0.996818134 | stratum I | 18 | 14 | 25 | 42 | 55 | 52 |
| XLOC_022356 | 17.07514206 | -1.079768355 | stratum I | 8 | 15 | 0 | 18 | 27 | 0 |
| XLOC_021256 | 1304.060493 | -0.446328742 | stratum I | 28 | 38 | 263 | 80 | 104 | 600 |
| XLOC_021257 | 3737.991269 | -0.711675376 | stratum I | 33 | 55 | 21 | 57 | 125 | 41 |
| XLOC_021258 | 1798.964188 | -0.211076518 | stratum I | 82 | 129 | 111 | 148 | 200 | 218 |
| XLOC_022357 | 12.58992641 | 0.89745834 | stratum I | 1 | 14 | 0 | 3 | 46 | 0 |
| XLOC_021259 | 584.1043469 | -0.240476775 | stratum I | 19 | 38 | 102 | 34 | 112 | 216 |
| XLOC_022358 | 6.858547089 | -0.234645348 | stratum I | 4 | 29 | 0 | 13 | 72 | 0 |
| XLOC_022359 | 16.1743598 | 1.509536914 | stratum I | 13 | 28 | 0 | 26 | 71 | 0 |
| XLOC_022360 | 6.99318152 | 1.578766405 | stratum I | 8 | 31 | 0 | 11 | 82 | 0 |
| XLOC_021260 | 524.2665851 | 0.021416691 | stratum I | 53 | 14 | 286 | 78 | 42 | 614 |
| XLOC_022361 | 29.72498328 | 1.019985829 | stratum I | 1 | 23 | 0 | 2 | 56 | 0 |
| XLOC_022362 | 5.063446122 | -0.248879327 | stratum I | 11 | 18 | 0 | 20 | 46 | 0 |
| XLOC_021261 | 301.6398848 | 0.260997913 | stratum I | 9 | 24 | 0 | 36 | 49 | 0 |
| XLOC_022363 | 8.260634135 | 0.547489399 | stratum I | 0 | 22 | 0 | 6 | 44 | 0 |
| XLOC_021817 | 137.4613009 | -0.351465884 | stratum I | 9 | 38 | 115 | 27 | 81 | 255 |
| XLOC_022364 | 6.05370008 | 2.054673083 | stratum I | 5 | 22 | 0 | 4 | 56 | 0 |
| XLOC_021818 | 811.0283296 | -0.432619803 | stratum I | 9 | 33 | 25 | 34 | 59 | 64 |
| XLOC_021262 | 259.0748414 | -0.466273802 | stratum I | 18 | 24 | 3 | 23 | 43 | 14 |
| XLOC_021819 | 2140.056353 | -0.343760466 | stratum I | 9 | 41 | 3 | 16 | 89 | 4 |
| XLOC_021263 | 710.1949638 | -0.273898975 | stratum I | 17 | 19 | 13 | 27 | 46 | 40 |
| XLOC_021264 | 1370.608834 | 0.033329589 | stratum I | 23 | 36 | 291 | 48 | 87 | 777 |
| XLOC_021820 | 8.114635013 | -0.500236545 | stratum I | 8 | 19 | 3 | 24 | 36 | 14 |
| XLOC_021265 | 242.1518582 | -0.795865701 | stratum I | 8 | 7 | 2 | 9 | 41 | 9 |
| XLOC_021821 | 1119.112361 | -0.833300834 | stratum I | 8 | 39 | 49 | 32 | 73 | 119 |
| XLOC_022365 | 5.953093138 | -0.730356068 | stratum I | 0 | 3 | 0 | 2 | 20 | 0 |
| XLOC_022366 | 635.5243042 | -1.072515366 | stratum I | 1 | 2 | 0 | 9 | 18 | 0 |
| XLOC_021266 | 3042.390996 | 0.108841216 | stratum I | 81 | 80 | 157 | 151 | 173 | 278 |
| XLOC_022367 | 64.38172119 | -0.160977275 | stratum I | 13 | 44 | 0 | 14 | 75 | 0 |
| XLOC_022368 | 57.7187039 | -0.82093995 | stratum I | 0 | 65 | 0 | 6 | 93 | 0 |
| XLOC_021822 | 1294.833165 | -0.67463494 | stratum I | 11 | 32 | 49 | 44 | 65 | 112 |
| XLOC_022369 | 5.634635248 | 1.134122304 | stratum I | 6 | 19 | 0 | 9 | 40 | 0 |
| XLOC_022370 | 4.453185047 | 0.601990217 | stratum I | 0 | 34 | 0 | 5 | 67 | 0 |
| XLOC_022371 | 2.813722888 | 1.156857157 | stratum I | 1 | 44 | 0 | 2 | 81 | 0 |
| XLOC_022372 | 2.26139082 | 1.0502298 | stratum I | 3 | 46 | 0 | 3 | 94 | 0 |
| XLOC_021823 | 29.43607931 | -0.58658347 | stratum I | 0 | 0 | 0 | 0 | 0 | 0 |
| XLOC_021824 | 33.3638556 | 1.388785483 | stratum I | 24 | 28 | 10 | 47 | 81 | 25 |
| XLOC_022373 | 46.43193782 | -0.946534688 | stratum I | 13 | 32 | 0 | 19 | 62 | 0 |
| XLOC_021825 | 54.52339628 | -1.083253295 | stratum I | 2 | 17 | 1 | 1 | 70 | 18 |
| XLOC_021267 | 5508.974455 | -0.253402431 | stratum I | 67 | 117 | 266 | 213 | 331 | 647 |
| XLOC_021826 | 135.5721998 | -0.626589097 | stratum I | 15 | 35 | 15 | 54 | 102 | 43 |
| XLOC_022374 | 123.7297191 | -0.516679238 | stratum I | 41 | 28 | 0 | 84 | 70 | 0 |
| XLOC_021827 | 53.60480664 | -0.448182874 | stratum I | 46 | 54 | 22 | 88 | 114 | 34 |
| XLOC_021828 | 58.16149246 | -0.649891488 | stratum I | 28 | 84 | 136 | 82 | 141 | 341 |
| XLOC_022375 | 5.090759092 | -0.572820353 | stratum I | 6 | 6 | 0 | 7 | 34 | 0 |
| XLOC_021829 | 2328.395393 | -0.401762696 | stratum I | 35 | 27 | 274 | 57 | 74 | 577 |
| XLOC_021268 | 70.11661273 | -0.896810976 | stratum I | 39 | 38 | 56 | 73 | 81 | 131 |
| XLOC_021830 | 16.80364834 | -0.336120126 | stratum I | 32 | 23 | 9 | 54 | 80 | 30 |
| XLOC_022376 | 476.8832418 | -0.375974875 | stratum I | 20 | 18 | 0 | 65 | 58 | 0 |
| XLOC_021831 | 105.5129954 | 0.216076339 | stratum I | 33 | 19 | 325 | 74 | 54 | 795 |
| XLOC_022377 | 35.08916047 | -0.117335353 | stratum I | 5 | 4 | 0 | 35 | 27 | 0 |
| XLOC_022378 | 15.50455297 | -0.650760171 | stratum I | 7 | 13 | 0 | 23 | 43 | 0 |
| XLOC_021832 | 50.7749017 | -0.990198748 | stratum I | 8 | 17 | 3 | 29 | 34 | 28 |
| XLOC_021833 | 3.200336218 | -0.704762578 | stratum I | 1 | 10 | 4 | 3 | 20 | 10 |
| XLOC_021834 | 136.2842948 | 1.848997416 | stratum I | 28 | 53 | 114 | 52 | 108 | 252 |
| XLOC_021835 | 180.2517253 | -0.770613464 | stratum I | 24 | 17 | 26 | 42 | 40 | 72 |
| XLOC_021269 | 1637.221092 | -0.876200206 | stratum I | 0 | 0 | 0 | 0 | 0 | 0 |
| XLOC_021271 | 25.89557173 | -0.83795517 | stratum I | 1 | 3 | 1 | 0 | 12 | 20 |
| XLOC_022379 | 2.215520698 | -0.429714231 | stratum I | 0 | 1 | 0 | 0 | 20 | 0 |
| XLOC_022380 | 10.84518604 | 1.001529269 | stratum I | 1 | 0 | 0 | 15 | 5 | 0 |
| XLOC_022381 | 6.976500321 | -0.829551076 | stratum I | 0 | 0 | 0 | 0 | 5 | 0 |
| XLOC_022383 | 9.958233448 | -1.013430635 | stratum I | 0 | 0 | 0 | 0 | 0 | 0 |
| XLOC_021272 | 50.45249515 | -0.465138687 | stratum I | 36 | 31 | 103 | 86 | 87 | 229 |
| XLOC_021273 | 177.0942049 | -1.050016805 | stratum I | 71 | 50 | 132 | 141 | 92 | 278 |
| XLOC_021838 | 322.0888067 | 0.105550372 | stratum I | 61 | 37 | 156 | 113 | 80 | 248 |
| XLOC_021274 | 276.8714225 | 0.375907309 | stratum I | 16 | 39 | 40 | 20 | 74 | 84 |
| XLOC_021839 | 180.6550892 | -0.116372674 | stratum I | 38 | 26 | 23 | 68 | 61 | 46 |
| XLOC_021275 | 2441.367896 | -0.650706054 | stratum I | 16 | 14 | 25 | 38 | 55 | 77 |
| XLOC_022384 | 24.61542413 | -0.063037822 | stratum I | 2 | 30 | 0 | 4 | 62 | 0 |
| XLOC_021276 | 155.4819198 | -0.667569645 | stratum I | 14 | 31 | 29 | 60 | 49 | 62 |
| XLOC_021277 | 1774.156179 | -0.319401076 | stratum I | 37 | 94 | 103 | 74 | 199 | 231 |
| XLOC_021278 | 635.4889608 | -0.940835048 | stratum I | 9 | 13 | 15 | 34 | 64 | 54 |
| XLOC_021841 | 4.078452272 | -0.599432288 | stratum I | 0 | 0 | 0 | 11 | 21 | 6 |
| XLOC_021842 | 111.88969 | -1.272407834 | stratum I | 0 | 0 | 0 | 5 | 34 | 24 |
| XLOC_022385 | 3.882902533 | 1.401951489 | stratum I | 0 | 9 | 0 | 1 | 58 | 0 |
| XLOC_021843 | 186.0310263 | 0.323538726 | stratum I | 19 | 8 | 9 | 30 | 57 | 37 |
| XLOC_021844 | 3630.656773 | -0.096347697 | stratum I | 43 | 49 | 55 | 86 | 116 | 122 |
| XLOC_022386 | 7.802015721 | -0.498695776 | stratum I | 3 | 26 | 0 | 8 | 54 | 0 |
| XLOC_021279 | 1700.368645 | -0.026997759 | stratum I | 19 | 36 | 20 | 35 | 75 | 37 |
| XLOC_021845 | 4.84038166 | -0.131224994 | stratum I | 24 | 36 | 2 | 45 | 65 | 4 |
| XLOC_021846 | 35.84938874 | -1.069188775 | stratum I | 11 | 7 | 13 | 33 | 0 | 59 |
| XLOC_021280 | 45.93337645 | -0.731157906 | stratum I | 41 | 29 | 38 | 61 | 51 | 85 |
| XLOC_021847 | 1305.210926 | 0.131731715 | stratum I | 42 | 26 | 17 | 70 | 62 | 20 |
| XLOC_021848 | 550.102027 | -0.126589872 | stratum I | 20 | 67 | 60 | 49 | 135 | 112 |
| XLOC_022387 | 15.14274689 | -0.459463566 | stratum I | 7 | 30 | 0 | 25 | 62 | 0 |
| XLOC_021281 | 361.8655502 | -0.077840904 | stratum I | 81 | 28 | 32 | 129 | 60 | 58 |
| XLOC_021282 | 2187.398517 | 0.04980923 | stratum I | 64 | 46 | 8 | 130 | 88 | 23 |
| XLOC_021283 | 242.3259681 | -0.518807601 | stratum I | 32 | 50 | 15 | 64 | 99 | 17 |
| XLOC_021849 | 2685.189879 | -0.646193774 | stratum I | 55 | 66 | 123 | 115 | 143 | 270 |
| XLOC_022388 | 7.083640585 | -0.521665394 | stratum I | 1 | 14 | 0 | 5 | 46 | 0 |
| XLOC_021284 | 963.7577549 | 0.468805468 | stratum I | 24 | 39 | 59 | 55 | 94 | 112 |
| XLOC_022389 | 5.562867785 | -0.029886862 | stratum I | 6 | 29 | 0 | 6 | 67 | 0 |
| XLOC_021850 | 2742.199985 | -0.508351504 | stratum I | 46 | 55 | 120 | 104 | 105 | 293 |
| XLOC_021285 | 72.30858843 | 0.377524296 | stratum I | 34 | 24 | 143 | 82 | 87 | 297 |
| XLOC_021286 | 284.9925151 | -0.136907499 | stratum I | 15 | 32 | 114 | 46 | 82 | 299 |
| XLOC_021287 | 245.8643683 | -0.460134488 | stratum I | 16 | 15 | 98 | 50 | 54 | 243 |
| XLOC_022390 | 29.51014748 | 2.648312462 | stratum I | 5 | 22 | 0 | 14 | 67 | 0 |
| XLOC_021288 | 5584.987891 | -0.085046673 | stratum I | 150 | 66 | 303 | 319 | 132 | 683 |
| XLOC_021289 | 579.115105 | -0.0639283 | stratum I | 37 | 69 | 277 | 86 | 166 | 681 |
| XLOC_021290 | 405.9112327 | 1.623110223 | stratum I | 12 | 47 | 100 | 50 | 161 | 241 |
| XLOC_021851 | 2158.164196 | -0.449246855 | stratum I | 27 | 26 | 4 | 66 | 41 | 12 |
| XLOC_022391 | 13.29044164 | 0.85978842 | stratum I | 4 | 26 | 0 | 8 | 65 | 0 |
| XLOC_022392 | 2.718094691 | 0.919996334 | stratum I | 3 | 32 | 0 | 5 | 70 | 0 |
| XLOC_022393 | 3.669378995 | -0.292054924 | stratum I | 5 | 15 | 0 | 14 | 62 | 0 |
| XLOC_021291 | 48.95097027 | -0.843088103 | stratum I | 0 | 16 | 0 | 7 | 55 | 0 |
| XLOC_021852 | 1035.815349 | 0.03273619 | stratum I | 31 | 37 | 30 | 38 | 78 | 80 |
| XLOC_021853 | 112.684388 | 0.188088507 | stratum I | 10 | 41 | 11 | 34 | 73 | 30 |
| XLOC_021292 | 199.5716587 | -0.666964238 | stratum I | 37 | 50 | 6 | 78 | 74 | 22 |
| XLOC_021854 | 24.126471 | -0.609352962 | stratum I | 16 | 23 | 38 | 14 | 63 | 83 |
| XLOC_021855 | 124.838864 | -0.605293519 | stratum I | 16 | 14 | 8 | 41 | 41 | 28 |
| XLOC_022394 | 13.35679009 | -0.164872007 | stratum I | 2 | 41 | 0 | 8 | 76 | 0 |
| XLOC_021293 | 438.6667019 | 0.048583414 | stratum I | 33 | 67 | 356 | 101 | 147 | 873 |
| XLOC_022395 | 2.688043057 | 0.740878512 | stratum I | 0 | 19 | 0 | 1 | 31 | 0 |
| XLOC_022396 | 3.689214366 | 1.350874199 | stratum I | 5 | 25 | 0 | 9 | 48 | 0 |
| XLOC_022397 | 19.60445814 | -0.663843869 | stratum I | 7 | 17 | 0 | 8 | 50 | 0 |
| XLOC_022398 | 24.44710002 | -0.472783866 | stratum I | 9 | 28 | 0 | 19 | 47 | 0 |
| XLOC_022399 | 8.590692608 | -0.286694667 | stratum I | 11 | 12 | 0 | 17 | 20 | 0 |
| XLOC_022400 | 46.69688707 | -0.293557202 | stratum I | 26 | 31 | 0 | 51 | 64 | 0 |
| XLOC_022401 | 574.8306735 | 0.021331768 | stratum I | 8 | 23 | 0 | 13 | 51 | 0 |
| XLOC_022402 | 2178.128582 | -0.388656706 | stratum I | 12 | 21 | 0 | 21 | 56 | 0 |
| XLOC_021856 | 97.32421631 | -0.950659419 | stratum I | 12 | 31 | 11 | 26 | 82 | 12 |
| XLOC_021294 | 9.809773592 | -0.48732469 | stratum I | 2 | 23 | 22 | 12 | 45 | 44 |
| XLOC_021857 | 610.0814287 | -0.219689221 | stratum I | 20 | 35 | 10 | 54 | 64 | 9 |
| XLOC_021295 | 23.57780109 | -0.02000648 | stratum I | 14 | 25 | 25 | 34 | 63 | 76 |
| XLOC_021296 | 1988.441031 | -0.092174249 | stratum I | 15 | 28 | 24 | 27 | 72 | 59 |
| XLOC_022403 | 10.61094093 | -0.06617978 | stratum I | 3 | 27 | 0 | 13 | 57 | 0 |
| XLOC_021299 | 505.8615294 | 0.470814164 | stratum I | 30 | 32 | 12 | 86 | 100 | 32 |
| XLOC_021859 | 367.149492 | -0.013953971 | stratum I | 40 | 51 | 628 | 99 | 168 | 1478 |
| XLOC_022404 | 10.18869221 | 0.702047453 | stratum I | 2 | 29 | 0 | 11 | 85 | 0 |
| XLOC_021300 | 28.39017859 | -0.385949885 | stratum I | 1 | 18 | 5 | 4 | 28 | 12 |
| XLOC_021301 | 13.3710084 | -0.523945974 | stratum I | 0 | 10 | 0 | 0 | 19 | 0 |
| XLOC_021302 | 370.0903492 | 0.05000857 | stratum I | 18 | 26 | 16 | 70 | 37 | 40 |
| XLOC_021861 | 1069.469319 | -0.088075493 | stratum I | 42 | 24 | 87 | 103 | 81 | 160 |
| XLOC_022405 | 12.19446749 | -0.010372221 | stratum I | 1 | 25 | 0 | 7 | 43 | 0 |
| XLOC_021862 | 25.00429964 | -0.271849963 | stratum I | 0 | 9 | 1 | 7 | 47 | 16 |
| XLOC_021304 | 5.271305146 | 0.783201107 | stratum I | 4 | 7 | 4 | 16 | 31 | 13 |
| XLOC_022406 | 8.089093253 | 0.054170895 | stratum I | 0 | 4 | 0 | 0 | 20 | 0 |
| XLOC_022407 | 14.17455944 | -0.445449672 | stratum I | 0 | 0 | 0 | 0 | 1 | 0 |
| XLOC_021305 | 788.4872097 | -0.8247114 | stratum I | 0 | 0 | 0 | 0 | 0 | 0 |
| XLOC_022408 | 15.31972376 | -0.539868509 | stratum I | 0 | 0 | 0 | 0 | 0 | 0 |
| XLOC_021306 | 64.15108483 | -0.652755483 | stratum I | 6 | 11 | 0 | 17 | 41 | 0 |
| XLOC_021863 | 561.2283474 | 0.513060613 | stratum I | 7 | 22 | 27 | 28 | 51 | 82 |
| XLOC_021864 | 463.2537953 | 0.220493226 | stratum I | 38 | 34 | 27 | 61 | 77 | 46 |
| XLOC_021307 | 2346.755733 | -0.103004784 | stratum I | 36 | 65 | 63 | 66 | 118 | 109 |
| XLOC_022409 | 107.9544525 | -0.903271847 | stratum I | 11 | 40 | 0 | 24 | 72 | 0 |
| XLOC_021308 | 110.5532133 | -0.741577029 | stratum I | 26 | 39 | 33 | 52 | 68 | 96 |
| XLOC_022410 | 20.77837933 | -0.64308683 | stratum I | 21 | 21 | 0 | 38 | 50 | 0 |
| XLOC_022411 | 5.720457491 | -0.581072305 | stratum I | 7 | 44 | 0 | 14 | 82 | 0 |
| XLOC_022412 | 4.123411656 | 0.92004097 | stratum I | 0 | 27 | 0 | 1 | 71 | 0 |
| XLOC_022413 | 23.415033 | -0.491742931 | stratum I | 21 | 32 | 0 | 48 | 70 | 0 |
| XLOC_021309 | 548.5060627 | -0.103862116 | stratum I | 53 | 44 | 255 | 115 | 80 | 519 |
| XLOC_022414 | 12.30163492 | 0.452629592 | stratum I | 16 | 13 | 0 | 29 | 37 | 0 |
| XLOC_022415 | 3.526519351 | 1.101803085 | stratum I | 2 | 22 | 0 | 2 | 50 | 0 |
| XLOC_021865 | 28.76349832 | -0.659515439 | stratum I | 11 | 32 | 416 | 23 | 53 | 944 |
| XLOC_022416 | 39.54570569 | 0.90199716 | stratum I | 10 | 5 | 0 | 25 | 36 | 0 |
| XLOC_021866 | 21.30221199 | -0.228116026 | stratum I | 6 | 40 | 82 | 11 | 95 | 146 |
| XLOC_021310 | 130.639112 | 0.115538976 | stratum I | 23 | 64 | 92 | 52 | 129 | 143 |
| XLOC_021867 | 3808.924462 | -0.167629211 | stratum I | 63 | 42 | 8 | 100 | 118 | 29 |
| XLOC_021868 | 1626.714208 | -0.072070822 | stratum I | 10 | 27 | 22 | 17 | 51 | 43 |
| XLOC_021311 | 114.0752997 | -0.673578691 | stratum I | 5 | 44 | 4 | 14 | 91 | 7 |
| XLOC_021869 | 846.6836633 | -0.200807631 | stratum I | 34 | 66 | 83 | 97 | 148 | 229 |
| XLOC_022417 | 6.382981085 | -0.263943922 | stratum I | 4 | 17 | 0 | 15 | 53 | 0 |
| XLOC_022418 | 9.334641575 | -0.190882691 | stratum I | 0 | 22 | 0 | 2 | 64 | 0 |
| XLOC_022419 | 4.873129039 | -0.050296723 | stratum I | 2 | 13 | 0 | 0 | 42 | 0 |
| XLOC_022420 | 3.877963155 | -0.561749589 | stratum I | 4 | 21 | 0 | 12 | 53 | 0 |
| XLOC_022421 | 18.54644015 | -0.845189736 | stratum I | 6 | 17 | 0 | 19 | 59 | 0 |
| XLOC_021312 | 1003.410447 | 0.306840888 | stratum I | 46 | 31 | 0 | 70 | 82 | 1 |
| XLOC_021870 | 1139.779124 | 1.705379661 | stratum I | 28 | 54 | 86 | 71 | 104 | 175 |
| XLOC_021313 | 1005.542064 | 0.026199337 | stratum I | 72 | 37 | 135 | 156 | 100 | 310 |
| XLOC_021871 | 152.3637804 | -0.809791132 | stratum I | 48 | 60 | 26 | 98 | 129 | 71 |
| XLOC_021314 | 675.2979654 | -0.570979867 | stratum I | 25 | 45 | 33 | 50 | 81 | 61 |
| XLOC_021315 | 306.8157802 | -0.421529891 | stratum I | 15 | 34 | 2 | 42 | 77 | 4 |
| XLOC_021872 | 327.2883129 | 0.106443968 | stratum I | 14 | 24 | 35 | 33 | 75 | 94 |
| XLOC_022422 | 46.00369924 | -0.539497742 | stratum I | 18 | 39 | 0 | 27 | 91 | 0 |
| XLOC_021316 | 8.296078754 | 0.183527398 | stratum I | 3 | 6 | 9 | 10 | 19 | 30 |
| XLOC_021317 | 600.342327 | -0.469324335 | stratum I | 4 | 25 | 14 | 20 | 59 | 22 |
| XLOC_021318 | 12.86235 | -0.827753745 | stratum I | 3 | 31 | 9 | 5 | 59 | 24 |
| XLOC_021320 | 371.5950991 | -0.291860624 | stratum I | 24 | 29 | 13 | 39 | 75 | 19 |
| XLOC_021321 | 4708.788658 | -0.074836351 | stratum I | 43 | 39 | 70 | 79 | 82 | 161 |
| XLOC_021322 | 836.5035753 | 0.31895747 | stratum I | 27 | 35 | 37 | 48 | 80 | 65 |
| XLOC_021323 | 599.9911015 | -0.538314948 | stratum I | 7 | 42 | 1 | 22 | 79 | 5 |
| XLOC_021324 | 394.7076984 | -0.24197163 | stratum I | 46 | 21 | 17 | 73 | 57 | 26 |
| XLOC_021873 | 1938.060081 | -0.024152844 | stratum I | 75 | 52 | 314 | 139 | 152 | 878 |
| XLOC_021325 | 9.600198259 | -0.937411622 | stratum I | 8 | 17 | 30 | 33 | 38 | 81 |
| XLOC_021327 | 103.8503033 | -0.902043401 | stratum I | 1 | 8 | 0 | 0 | 1 | 0 |
| XLOC_022423 | 10.13750617 | 0.774113204 | stratum I | 4 | 1 | 0 | 0 | 0 | 0 |
| XLOC_021877 | 1891.100641 | -0.495392522 | stratum I | 28 | 35 | 41 | 48 | 81 | 102 |
| XLOC_021328 | 50.24016258 | 0.02136224 | stratum I | 33 | 16 | 20 | 50 | 48 | 34 |
| XLOC_021878 | 882.3305622 | -1.077498159 | stratum I | 41 | 54 | 24 | 103 | 111 | 62 |
| XLOC_021329 | 8.160702709 | 0.488358207 | stratum I | 3 | 39 | 13 | 9 | 85 | 47 |
| XLOC_021880 | 270.8897906 | -0.479695091 | stratum I | 14 | 25 | 12 | 43 | 59 | 28 |
| XLOC_021331 | 175.884227 | 0.531103096 | stratum I | 20 | 24 | 34 | 65 | 67 | 79 |
| XLOC_021881 | 255.6042222 | -0.013067813 | stratum I | 16 | 24 | 51 | 53 | 51 | 104 |
| XLOC_022424 | 43.48354063 | -0.643844631 | stratum I | 7 | 16 | 0 | 10 | 42 | 0 |
| XLOC_021882 | 612.6629686 | -0.223121832 | stratum I | 19 | 49 | 90 | 30 | 82 | 202 |
| XLOC_021883 | 2279.343841 | -0.373600483 | stratum I | 58 | 111 | 272 | 123 | 188 | 574 |
| XLOC_022425 | 10.98085037 | -0.454571288 | stratum I | 1 | 29 | 0 | 9 | 89 | 0 |
| XLOC_022426 | 17.00914187 | -0.679825509 | stratum I | 0 | 34 | 0 | 11 | 86 | 0 |
| XLOC_022427 | 15.10232991 | -1.116456558 | stratum I | 12 | 41 | 0 | 21 | 66 | 0 |
| XLOC_021884 | 241.6019857 | -1.207251274 | stratum I | 14 | 49 | 0 | 23 | 64 | 0 |
| XLOC_021332 | 40.828086 | -0.876762185 | stratum I | 20 | 44 | 29 | 34 | 57 | 96 |
| XLOC_022428 | 7.84577684 | -0.637144538 | stratum I | 18 | 35 | 0 | 19 | 62 | 0 |
| XLOC_021333 | 18.57091667 | 2.706817368 | stratum I | 10 | 25 | 72 | 25 | 45 | 117 |
| XLOC_021885 | 10.02235612 | -0.581013206 | stratum I | 7 | 17 | 6 | 20 | 39 | 16 |
| XLOC_021334 | 919.0773876 | -0.981630068 | stratum I | 0 | 10 | 11 | 4 | 41 | 36 |
| XLOC_021335 | 22.52441989 | 1.543021299 | stratum I | 17 | 57 | 33 | 19 | 98 | 68 |
| XLOC_021336 | 2306.3619 | -0.071196491 | stratum I | 50 | 78 | 94 | 96 | 143 | 166 |
| XLOC_021886 | 263.3272609 | -0.870890874 | stratum I | 1 | 22 | 12 | 8 | 57 | 25 |
| XLOC_021337 | 528.9818648 | -0.236901894 | stratum I | 44 | 27 | 193 | 122 | 76 | 508 |
| XLOC_022429 | 30.82749 | -0.656262537 | stratum I | 8 | 21 | 0 | 33 | 78 | 0 |
| XLOC_021887 | 76.53931124 | -0.827013809 | stratum I | 10 | 26 | 23 | 31 | 64 | 51 |
| XLOC_021338 | 788.4753056 | -0.170249863 | stratum I | 17 | 27 | 9 | 19 | 55 | 12 |
| XLOC_022430 | 1432.741799 | -0.216368451 | stratum I | 11 | 28 | 0 | 26 | 52 | 0 |
| XLOC_021888 | 1411.589604 | -0.15593217 | stratum I | 21 | 28 | 23 | 63 | 55 | 48 |
| XLOC_022431 | 56.2763306 | 0.322971662 | stratum I | 0 | 4 | 0 | 1 | 26 | 0 |
| XLOC_021339 | 2916.944431 | 0.200265636 | stratum I | 24 | 24 | 37 | 49 | 72 | 81 |
| XLOC_022432 | 6.115326072 | -0.804152546 | stratum I | 0 | 8 | 0 | 0 | 28 | 0 |
| XLOC_022433 | 119.8084077 | -1.076607252 | stratum I | 13 | 17 | 0 | 34 | 37 | 0 |
| XLOC_022434 | 569.2300049 | -0.380696655 | stratum I | 119 | 25 | 0 | 212 | 58 | 0 |
| XLOC_022435 | 29.7428594 | -0.559246523 | stratum I | 20 | 25 | 0 | 59 | 70 | 0 |
| XLOC_022436 | 12.57485241 | -0.224845551 | stratum I | 6 | 33 | 0 | 11 | 53 | 0 |
| XLOC_021340 | 41.55371907 | -0.324521947 | stratum I | 19 | 20 | 2 | 25 | 46 | 4 |
| XLOC_022437 | 6.376939466 | -0.142154804 | stratum I | 4 | 21 | 0 | 8 | 62 | 0 |
| XLOC_021341 | 12.83364409 | 2.419169221 | stratum I | 8 | 52 | 253 | 18 | 116 | 500 |
| XLOC_022438 | 95.44961919 | -0.581666874 | stratum I | 30 | 28 | 0 | 60 | 71 | 0 |
| XLOC_022439 | 4.07514494 | 1.519287457 | stratum I | 4 | 33 | 0 | 5 | 74 | 0 |
| XLOC_022440 | 12.08497196 | 0.604099654 | stratum I | 11 | 16 | 0 | 20 | 58 | 0 |
| XLOC_022441 | 10.20394966 | -0.606331465 | stratum I | 7 | 36 | 0 | 19 | 56 | 0 |
| XLOC_022442 | 6.867153973 | 0.610545366 | stratum I | 4 | 26 | 0 | 7 | 52 | 0 |
| XLOC_022443 | 23.850074 | -0.561715863 | stratum I | 22 | 34 | 0 | 56 | 71 | 0 |
| XLOC_021889 | 450.4243864 | 0.033757634 | stratum I | 28 | 44 | 167 | 32 | 92 | 337 |
| XLOC_022444 | 13.74132514 | 0.241737984 | stratum I | 11 | 40 | 0 | 10 | 76 | 0 |
| XLOC_021342 | 126.7424743 | -0.600496031 | stratum I | 40 | 36 | 40 | 57 | 91 | 55 |
| XLOC_021890 | 2703.133683 | -0.121132282 | stratum I | 20 | 42 | 33 | 43 | 69 | 68 |
| XLOC_021343 | 7.405277768 | -0.411232873 | stratum I | 7 | 18 | 29 | 10 | 54 | 33 |
| XLOC_021344 | 342.3703748 | 0.204092641 | stratum I | 22 | 64 | 20 | 57 | 86 | 52 |
| XLOC_021891 | 89.98503639 | 1.31975305 | stratum I | 21 | 29 | 18 | 42 | 65 | 27 |
| XLOC_021345 | 635.7905015 | -0.603069127 | stratum I | 7 | 25 | 12 | 19 | 55 | 21 |
| XLOC_021346 | 1076.710804 | -0.505484941 | stratum I | 40 | 42 | 39 | 79 | 80 | 80 |
| XLOC_022445 | 57.43948109 | -0.630638846 | stratum I | 2 | 16 | 0 | 5 | 36 | 0 |
| XLOC_021892 | 48.6676887 | -0.249610994 | stratum I | 21 | 28 | 45 | 33 | 74 | 99 |
| XLOC_021893 | 70.71973667 | -0.786393044 | stratum I | 12 | 33 | 1 | 9 | 72 | 1 |
| XLOC_021347 | 436.6081178 | -0.7911807 | stratum I | 12 | 48 | 20 | 24 | 119 | 56 |
| XLOC_021894 | 837.3004424 | -0.606232743 | stratum I | 35 | 45 | 44 | 81 | 89 | 84 |
| XLOC_022446 | 3.083756772 | -0.27840375 | stratum I | 0 | 35 | 0 | 3 | 78 | 0 |
| XLOC_021348 | 1273.532226 | -0.30621695 | stratum I | 27 | 45 | 69 | 61 | 111 | 127 |
| XLOC_021895 | 967.7027272 | -0.640095129 | stratum I | 15 | 23 | 150 | 29 | 32 | 301 |
| XLOC_022447 | 19.66845234 | 0.720276328 | stratum I | 3 | 9 | 0 | 3 | 22 | 0 |
| XLOC_022448 | 3.334301909 | -0.45523817 | stratum I | 0 | 14 | 0 | 0 | 25 | 0 |
| XLOC_022449 | 30.71645967 | 4.187792788 | stratum I | 5 | 18 | 0 | 16 | 35 | 0 |
| XLOC_022450 | 2.36575512 | -0.142026469 | stratum I | 9 | 5 | 0 | 20 | 16 | 0 |
| XLOC_021349 | 496.268451 | 0.023651692 | stratum I | 14 | 20 | 24 | 29 | 52 | 58 |
| XLOC_021350 | 53.77109218 | -0.653944903 | stratum I | 12 | 36 | 9 | 45 | 68 | 38 |
| XLOC_022451 | 8.745592557 | 2.55187999 | stratum I | 6 | 37 | 0 | 10 | 83 | 0 |
| XLOC_021897 | 736.6814889 | -0.213777033 | stratum I | 54 | 61 | 291 | 101 | 165 | 655 |
| XLOC_022452 | 2.720089075 | 0.725369746 | stratum I | 1 | 23 | 0 | 4 | 53 | 0 |
| XLOC_022453 | 6.948722728 | 0.811385071 | stratum I | 4 | 24 | 0 | 8 | 78 | 0 |
| XLOC_022454 | 7.612415177 | 0.485341035 | stratum I | 1 | 26 | 0 | 15 | 73 | 0 |
| XLOC_022455 | 6.744384689 | 1.403603703 | stratum I | 7 | 17 | 0 | 11 | 42 | 0 |
| XLOC_022456 | 16.88840235 | 3.523014688 | stratum I | 0 | 11 | 0 | 1 | 18 | 0 |
| XLOC_021353 | 1121.568709 | -0.986806924 | stratum I | 11 | 18 | 103 | 25 | 72 | 210 |
| XLOC_021354 | 1406.997089 | -0.643740473 | stratum I | 52 | 30 | 49 | 88 | 63 | 139 |
| XLOC_021898 | 61487.43073 | -0.599716681 | stratum I | 63 | 84 | 174 | 99 | 188 | 406 |
| XLOC_022457 | 9.086869529 | -0.609540301 | stratum I | 2 | 30 | 0 | 13 | 67 | 0 |
| XLOC_021899 | 163.4754361 | 0.521222485 | stratum I | 30 | 47 | 51 | 53 | 116 | 124 |
| XLOC_022458 | 3.526716009 | -0.114937236 | stratum I | 14 | 32 | 0 | 21 | 71 | 0 |
| XLOC_021355 | 583.0191863 | -0.571474116 | stratum I | 21 | 23 | 0 | 33 | 42 | 0 |
| XLOC_022459 | 14.16887462 | -0.664596573 | stratum I | 9 | 52 | 0 | 14 | 79 | 0 |
| XLOC_022460 | 3.852882969 | 1.585921722 | stratum I | 1 | 38 | 0 | 3 | 80 | 0 |
| XLOC_022461 | 36.28458837 | 3.91540973 | stratum I | 5 | 34 | 0 | 21 | 62 | 0 |
| XLOC_021356 | 301.4980558 | -0.855233611 | stratum I | 35 | 18 | 167 | 62 | 50 | 385 |
| XLOC_021357 | 209.7217062 | -0.686938289 | stratum I | 2 | 6 | 150 | 32 | 34 | 372 |
| XLOC_021900 | 59.19266025 | -0.37618962 | stratum I | 8 | 25 | 0 | 14 | 70 | 0 |
| XLOC_022462 | 6.288608225 | -0.44520001 | stratum I | 0 | 26 | 0 | 17 | 67 | 0 |
| XLOC_021358 | 176.3801424 | -0.661173785 | stratum I | 35 | 57 | 36 | 69 | 100 | 58 |
| XLOC_021901 | 358.0363175 | 0.296228261 | stratum I | 75 | 60 | 47 | 94 | 99 | 100 |
| XLOC_021902 | 599.3720468 | -0.361539741 | stratum I | 29 | 35 | 119 | 66 | 67 | 188 |
| XLOC_021903 | 26.52446542 | 0.012831212 | stratum I | 23 | 35 | 49 | 32 | 87 | 100 |
| XLOC_021360 | 1211.009765 | -0.000766221 | stratum I | 28 | 45 | 67 | 54 | 68 | 120 |
| XLOC_021361 | 706.7338493 | 0.062240888 | stratum I | 14 | 47 | 25 | 37 | 57 | 78 |
| XLOC_021904 | 6484.940151 | -0.648875045 | stratum I | 17 | 25 | 16 | 38 | 61 | 30 |
| XLOC_021362 | 535.3689081 | 0.013065713 | stratum I | 27 | 45 | 587 | 48 | 103 | 1408 |
| XLOC_022463 | 38.7611021 | -0.936951679 | stratum I | 0 | 29 | 0 | 2 | 62 | 0 |
| XLOC_022464 | 19.7932602 | 0.335856095 | stratum I | 1 | 22 | 0 | 7 | 62 | 0 |
| XLOC_022465 | 1250.187586 | -0.253188542 | stratum I | 5 | 7 | 0 | 18 | 22 | 0 |
| XLOC_021905 | 146.6669075 | 0.208103056 | stratum I | 9 | 27 | 157 | 11 | 68 | 377 |
| XLOC_021906 | 1179.76161 | 0.197810748 | stratum I | 34 | 42 | 241 | 68 | 100 | 661 |
| XLOC_022466 | 44.65104259 | -0.069757532 | stratum I | 2 | 15 | 0 | 20 | 35 | 0 |
| XLOC_022467 | 36.18716688 | -0.457301095 | stratum I | 12 | 24 | 0 | 20 | 56 | 0 |
| XLOC_022468 | 43.79812372 | -0.708619219 | stratum I | 9 | 13 | 0 | 33 | 47 | 0 |
| XLOC_022469 | 3.42104467 | 1.289460908 | stratum I | 1 | 16 | 0 | 3 | 29 | 0 |
| XLOC_022470 | 5.414246061 | 0.846902648 | stratum I | 10 | 3 | 0 | 12 | 14 | 0 |
| XLOC_021363 | 393.9339455 | -0.682650163 | stratum I | 25 | 34 | 28 | 91 | 100 | 68 |
| XLOC_022471 | 317.2775133 | -0.601481143 | stratum I | 7 | 7 | 0 | 20 | 35 | 0 |
| XLOC_021907 | 444.3381693 | -0.37238301 | stratum I | 9 | 25 | 69 | 25 | 51 | 102 |
| XLOC_021364 | 134.4261081 | 0.15914049 | stratum I | 12 | 12 | 23 | 22 | 33 | 27 |
| XLOC_021365 | 165.6342707 | 0.044881977 | stratum I | 26 | 36 | 15 | 53 | 63 | 30 |
| XLOC_021908 | 101.0446927 | 0.269096703 | stratum I | 19 | 16 | 114 | 30 | 24 | 183 |
| XLOC_021366 | 168.7976639 | -0.140729066 | stratum I | 0 | 23 | 1 | 20 | 60 | 6 |
| XLOC_022472 | 7.822896578 | -0.135521906 | stratum I | 0 | 0 | 0 | 0 | 7 | 0 |
| XLOC_021909 | 250.1480125 | -1.100204481 | stratum I | 0 | 10 | 0 | 3 | 25 | 3 |
| XLOC_021367 | 2790.763084 | -0.097417353 | stratum I | 25 | 3 | 122 | 31 | 8 | 177 |
| XLOC_021910 | 277.4150867 | -0.649825083 | stratum I | 29 | 15 | 57 | 32 | 46 | 114 |
| XLOC_021368 | 1147.459323 | -0.847670386 | stratum I | 16 | 55 | 401 | 38 | 135 | 727 |
| XLOC_022473 | 34.44019853 | 3.178776239 | stratum I | 2 | 14 | 0 | 4 | 38 | 0 |
| XLOC_022474 | 12.05326696 | 2.046101416 | stratum I | 11 | 21 | 0 | 13 | 52 | 0 |
| XLOC_022475 | 4.144092631 | 0.910135465 | stratum I | 4 | 13 | 0 | 1 | 34 | 0 |
| XLOC_022476 | 27.15211746 | 2.914273649 | stratum I | 17 | 14 | 0 | 21 | 40 | 0 |
| XLOC_022477 | 48.55468539 | 0.171161129 | stratum I | 17 | 17 | 0 | 35 | 26 | 0 |
| XLOC_022478 | 5.498077807 | -0.160674259 | stratum I | 5 | 21 | 0 | 5 | 37 | 0 |
| XLOC_022479 | 36.8814355 | -0.836455554 | stratum I | 16 | 32 | 0 | 19 | 29 | 0 |
| XLOC_021369 | 530.7230158 | 0.05736557 | stratum I | 11 | 12 | 0 | 13 | 34 | 0 |
| XLOC_022480 | 85.97999112 | -0.165569008 | stratum I | 4 | 13 | 0 | 8 | 18 | 0 |
| XLOC_021911 | 95.22908316 | -0.254736445 | stratum I | 2 | 20 | 13 | 1 | 34 | 27 |
| XLOC_021912 | 189.3047257 | -0.909962471 | stratum I | 2 | 11 | 5 | 7 | 25 | 13 |
| XLOC_022481 | 12.68414804 | -0.748318209 | stratum I | 3 | 8 | 0 | 6 | 20 | 0 |
| XLOC_021370 | 2841.971301 | -0.092666521 | stratum I | 10 | 28 | 39 | 45 | 69 | 102 |
| XLOC_021371 | 112.483181 | -0.401640113 | stratum I | 3 | 29 | 0 | 10 | 56 | 0 |
| XLOC_021913 | 415.561573 | 0.247917477 | stratum I | 7 | 48 | 200 | 12 | 109 | 436 |
| XLOC_021915 | 62.27822239 | -1.41602316 | stratum I | 40 | 20 | 98 | 114 | 83 | 138 |
| XLOC_021916 | 6.572188602 | -0.152457462 | stratum I | 2 | 9 | 1 | 9 | 27 | 5 |
| XLOC_021373 | 3791.428249 | -0.016572384 | stratum I | 25 | 8 | 58 | 41 | 44 | 124 |
| XLOC_022482 | 18.0673103 | -0.036861115 | stratum I | 7 | 23 | 0 | 8 | 60 | 0 |
| XLOC_021374 | 370.0027596 | 0.120802742 | stratum I | 13 | 50 | 116 | 24 | 109 | 282 |
| XLOC_021375 | 185.5580306 | 0.187309918 | stratum I | 4 | 34 | 71 | 2 | 78 | 187 |
| XLOC_021917 | 2535.640541 | -0.312324168 | stratum I | 19 | 55 | 189 | 52 | 157 | 461 |
| XLOC_022483 | 47.73320742 | -0.598765846 | stratum I | 0 | 16 | 0 | 1 | 28 | 0 |
| XLOC_022484 | 30.31486167 | -0.911192769 | stratum I | 12 | 19 | 0 | 25 | 39 | 0 |
| XLOC_021377 | 204.614493 | -0.753413206 | stratum I | 0 | 0 | 0 | 0 | 5 | 0 |
| XLOC_021918 | 18.00146967 | -1.165924589 | stratum I | 0 | 0 | 0 | 2 | 11 | 3 |
| XLOC_021919 | 104.3323053 | -0.920722943 | stratum I | 0 | 0 | 0 | 9 | 5 | 8 |
| XLOC_021378 | 2.835337912 | -0.239926202 | stratum I | 0 | 0 | 0 | 0 | 0 | 0 |
| XLOC_021379 | 345.5411029 | -0.027704578 | stratum I | 2 | 8 | 1 | 6 | 10 | 1 |
| XLOC_021920 | 56.9413761 | -0.132361621 | stratum I | 9 | 25 | 9 | 16 | 42 | 10 |
| XLOC_022485 | 7.1309369 | 1.875488219 | stratum I | 4 | 21 | 0 | 9 | 39 | 0 |
| XLOC_021921 | 518.8077298 | 0.074629805 | stratum I | 61 | 68 | 63 | 117 | 116 | 88 |
| XLOC_021380 | 11.43537051 | -0.253860532 | stratum I | 13 | 37 | 38 | 57 | 94 | 84 |
| XLOC_021922 | 34.86532606 | 0.564307379 | stratum I | 53 | 43 | 97 | 115 | 73 | 200 |
| XLOC_021381 | 87.03623121 | 0.682070463 | stratum I | 22 | 26 | 37 | 53 | 57 | 103 |
| XLOC_021382 | 1135.920003 | -0.540566635 | stratum I | 30 | 28 | 247 | 84 | 68 | 620 |
| XLOC_022487 | 12.27982242 | -0.131434818 | stratum I | 5 | 13 | 0 | 7 | 45 | 0 |
| XLOC_022488 | 33.21689912 | -0.328522302 | stratum I | 4 | 29 | 0 | 12 | 46 | 0 |
| XLOC_021923 | 311.4293242 | -0.869216964 | stratum I | 39 | 22 | 36 | 99 | 85 | 66 |
| XLOC_021924 | 2626.794537 | -0.720210101 | stratum I | 57 | 45 | 128 | 117 | 107 | 249 |
| XLOC_022489 | 4.691433013 | 0.640437563 | stratum I | 5 | 20 | 0 | 7 | 53 | 0 |
| XLOC_021383 | 43.55479997 | -0.518317046 | stratum I | 24 | 83 | 42 | 62 | 185 | 99 |
| XLOC_022490 | 9.457849802 | -0.492913023 | stratum I | 17 | 33 | 0 | 20 | 85 | 0 |
| XLOC_021385 | 92.60041506 | -0.971393143 | stratum I | 14 | 18 | 8 | 48 | 37 | 13 |
| XLOC_021925 | 16.01747354 | -1.074186765 | stratum I | 26 | 22 | 4 | 63 | 43 | 7 |
| XLOC_021386 | 8151.241512 | 0.041966689 | stratum I | 30 | 45 | 58 | 54 | 67 | 104 |
| XLOC_021926 | 98.28280757 | 0.310153163 | stratum I | 6 | 24 | 17 | 11 | 48 | 26 |
| XLOC_022491 | 19.09732832 | -0.328720689 | stratum I | 3 | 8 | 0 | 3 | 32 | 0 |
| XLOC_022492 | 107.6531159 | 5.812899231 | stratum I | 1 | 32 | 0 | 14 | 64 | 0 |
| XLOC_022493 | 14.54477193 | 3.222145073 | stratum I | 1 | 36 | 0 | 3 | 75 | 0 |
| XLOC_022494 | 156.5580182 | 6.395593413 | stratum I | 9 | 27 | 0 | 17 | 77 | 0 |
| XLOC_022495 | 7.273535023 | 2.303092914 | stratum I | 3 | 37 | 0 | 6 | 84 | 0 |
| XLOC_022496 | 17.89938023 | 3.471988942 | stratum I | 0 | 32 | 0 | 2 | 84 | 0 |
| XLOC_021927 | 31.76705866 | 4.40124231 | stratum I | 2 | 46 | 19 | 15 | 121 | 51 |
| XLOC_022497 | 18.64630847 | 3.392572671 | stratum I | 5 | 33 | 0 | 9 | 87 | 0 |
| XLOC_022498 | 21.99846756 | 3.732638185 | stratum I | 3 | 27 | 0 | 5 | 87 | 0 |
| XLOC_021387 | 266.9495575 | 0.719314083 | stratum I | 51 | 73 | 157 | 96 | 144 | 316 |
| XLOC_021388 | 70.97019671 | -0.356756894 | stratum I | 22 | 44 | 48 | 41 | 132 | 99 |
| XLOC_021929 | 11.55680918 | -0.344676666 | stratum I | 13 | 30 | 85 | 38 | 85 | 267 |
| XLOC_021389 | 229.9228356 | -0.521183859 | stratum I | 37 | 14 | 162 | 102 | 56 | 316 |
| XLOC_021930 | 91.90482621 | -1.069102146 | stratum I | 1 | 19 | 0 | 4 | 45 | 0 |
| XLOC_022499 | 6.171597632 | -0.732400675 | stratum I | 0 | 0 | 0 | 0 | 0 | 0 |
| XLOC_021390 | 341.3467034 | -1.036336642 | stratum I | 22 | 6 | 21 | 30 | 31 | 57 |
| XLOC_021391 | 76.76654885 | -0.812141508 | stratum I | 15 | 5 | 7 | 41 | 17 | 52 |
| XLOC_021934 | 817.5123455 | -0.878622528 | stratum I | 19 | 17 | 8 | 41 | 52 | 28 |
| XLOC_022500 | 5.125007183 | -0.013691062 | stratum I | 0 | 10 | 0 | 0 | 28 | 0 |
| XLOC_021392 | 2006.208176 | -0.968183861 | stratum I | 36 | 15 | 20 | 135 | 21 | 47 |
| XLOC_021935 | 525.3152226 | -0.445747833 | stratum I | 32 | 11 | 137 | 68 | 17 | 374 |
| XLOC_022501 | 16.33265383 | -0.060052816 | stratum I | 13 | 27 | 0 | 41 | 51 | 0 |
| XLOC_021394 | 367.408181 | -0.879797463 | stratum I | 27 | 30 | 29 | 53 | 59 | 82 |
| XLOC_021396 | 13.44463668 | -0.5219136 | stratum I | 6 | 14 | 0 | 10 | 48 | 16 |
| XLOC_021936 | 481.8622787 | -0.126824687 | stratum I | 39 | 51 | 54 | 109 | 127 | 128 |
| XLOC_021397 | 424.7187369 | -0.271158893 | stratum I | 24 | 16 | 0 | 57 | 55 | 0 |
| XLOC_021937 | 731.2814109 | -0.930200077 | stratum I | 20 | 46 | 25 | 32 | 81 | 47 |
| XLOC_022502 | 1128.319241 | -0.128986888 | stratum I | 27 | 21 | 0 | 24 | 49 | 0 |
| XLOC_021938 | 1872.518814 | -0.361701155 | stratum I | 31 | 57 | 715 | 57 | 101 | 1745 |
| XLOC_022503 | 5.593120974 | 1.831895608 | stratum I | 4 | 27 | 0 | 4 | 57 | 0 |
| XLOC_022504 | 6.0256827 | 1.892605066 | stratum I | 4 | 31 | 0 | 6 | 56 | 0 |
| XLOC_022505 | 4.566553338 | 0.949597156 | stratum I | 0 | 12 | 0 | 7 | 40 | 0 |
| XLOC_022506 | 10.67221898 | -0.256786571 | stratum I | 7 | 15 | 0 | 11 | 52 | 0 |
| XLOC_022507 | 3.037024622 | 0.701335724 | stratum I | 7 | 5 | 0 | 7 | 46 | 0 |
| XLOC_022508 | 9.243896221 | -0.213544429 | stratum I | 7 | 10 | 0 | 13 | 22 | 0 |
| XLOC_022509 | 5.000669153 | -0.307233337 | stratum I | 5 | 9 | 0 | 7 | 17 | 0 |
| XLOC_022510 | 14.42114119 | -0.343044543 | stratum I | 8 | 41 | 0 | 21 | 64 | 0 |
| XLOC_022511 | 5.862301907 | 1.685459289 | stratum I | 2 | 4 | 0 | 9 | 40 | 0 |
| XLOC_022512 | 60.81636214 | -0.26775895 | stratum I | 20 | 4 | 0 | 54 | 35 | 0 |
| XLOC_022513 | 61.65403705 | 0.496820509 | stratum I | 24 | 18 | 0 | 49 | 47 | 0 |
| XLOC_022514 | 62.60548096 | -0.599849928 | stratum I | 11 | 26 | 0 | 32 | 45 | 0 |
| XLOC_022515 | 72.16224422 | -0.829485212 | stratum I | 5 | 15 | 0 | 15 | 36 | 0 |
| XLOC_022516 | 33.67466899 | -0.731155671 | stratum I | 11 | 24 | 0 | 28 | 39 | 0 |
| XLOC_021939 | 46.85873643 | -0.965311837 | stratum I | 11 | 27 | 0 | 12 | 66 | 2 |
| XLOC_021398 | 2277.616425 | -0.012275933 | stratum I | 59 | 94 | 350 | 141 | 211 | 816 |
| XLOC_021399 | 1564.660952 | -0.736471533 | stratum I | 23 | 50 | 33 | 60 | 120 | 114 |
| XLOC_022517 | 46.91733974 | -0.317214665 | stratum I | 1 | 11 | 0 | 8 | 35 | 0 |
| XLOC_021400 | 158.5101208 | -1.056191297 | stratum I | 25 | 25 | 9 | 43 | 76 | 26 |
| XLOC_021401 | 528.908526 | -0.674533678 | stratum I | 3 | 15 | 50 | 15 | 54 | 132 |
| XLOC_022518 | 8.543790551 | -0.042879546 | stratum I | 3 | 18 | 0 | 12 | 61 | 0 |
| XLOC_022519 | 23.08596474 | -0.134435469 | stratum I | 10 | 21 | 0 | 21 | 64 | 0 |
| XLOC_022520 | 11.84526158 | 1.346527464 | stratum I | 10 | 27 | 0 | 21 | 64 | 0 |
| XLOC_021940 | 2836.346795 | -0.96302093 | stratum I | 8 | 14 | 27 | 17 | 53 | 71 |
| XLOC_021402 | 29.25117106 | -0.653820724 | stratum I | 10 | 19 | 6 | 14 | 72 | 31 |
| XLOC_021941 | 1004.950899 | -0.842932485 | stratum I | 26 | 53 | 265 | 70 | 124 | 610 |
| XLOC_021967 | 6411.889543 | -0.907820479 | stratum I | 0 | 0 | 0 | 0 | 0 | 0 |
| XLOC_021969 | 1407.824888 | -0.751370125 | stratum I | 0 | 5 | 13 | 0 | 4 | 21 |
| XLOC_021478 | 740.0627115 | -0.021359604 | stratum I | 0 | 0 | 2 | 0 | 0 | 20 |
| XLOC_022096 | 79.29217033 | -0.950241761 | stratum I | 0 | 0 | 0 | 0 | 0 | 0 |
| XLOC_022103 | 570.4463001 | -1.04710353 | stratum I | 0 | 0 | 0 | 0 | 0 | 0 |
| XLOC_022634 | 4.504904019 | 0.289789525 | stratum I | 0 | 0 | 0 | 0 | 0 | 0 |
| XLOC_021620 | 704.6724229 | -1.072244318 | stratum I | 0 | 0 | 0 | 0 | 0 | 0 |
| XLOC_022165 | 844.4453924 | -0.959970961 | stratum I | 0 | 0 | 0 | 0 | 0 | 0 |
| XLOC_022174 | 1457.044985 | -0.954847932 | stratum I | 0 | 0 | 0 | 0 | 0 | 0 |
| XLOC_021167 | 53.42464288 | -0.055404269 | stratum II | 0 | 0 | 0 | 0 | 10 | 5 |
| XLOC_022286 | 9.818771859 | -0.203711387 | stratum II | 0 | 0 | 1 | 0 | 2 | 2 |
| XLOC_021836 | 80.52826048 | -0.955734133 | stratum II | 0 | 0 | 0 | 0 | 0 | 0 |
| XLOC_021270 | 51.17209809 | -1.06843269 | stratum II | 0 | 0 | 0 | 0 | 0 | 0 |
| XLOC_021837 | 96.64774731 | -1.235528746 | stratum II | 0 | 7 | 0 | 0 | 14 | 0 |
| XLOC_022382 | 43.03289269 | -1.482789284 | stratum II | 0 | 0 | 0 | 0 | 0 | 0 |
| XLOC_021326 | 16.63003756 | -0.578738331 | stratum II | 0 | 0 | 0 | 0 | 12 | 0 |
| XLOC_021376 | 29.68706687 | -0.788555805 | stratum II | 0 | 0 | 3 | 0 | 0 | 2 |
| XLOC_021931 | 31.69147356 | -1.071156046 | stratum II | 0 | 0 | 0 | 0 | 0 | 0 |
| XLOC_021932 | 205.4187058 | -1.260713115 | stratum II | 0 | 0 | 0 | 0 | 0 | 0 |
| XLOC_021403 | 386.0563779 | -0.885464322 | stratum II | 24 | 44 | 33 | 63 | 101 | 86 |
| XLOC_021942 | 1373.873478 | -1.033087055 | stratum II | 18 | 28 | 9 | 37 | 79 | 15 |
| XLOC_021404 | 332.2196763 | -1.033213034 | stratum II | 0 | 14 | 0 | 0 | 55 | 0 |
| XLOC_021405 | 2.263466735 | -0.098692497 | stratum II | 6 | 9 | 0 | 12 | 31 | 0 |
| XLOC_021943 | 706.5942623 | -0.865850496 | stratum II | 2 | 14 | 8 | 23 | 32 | 30 |
| XLOC_021406 | 11.83230294 | -0.44180994 | stratum II | 21 | 43 | 63 | 68 | 147 | 198 |
| XLOC_021944 | 174.8591205 | -0.560638814 | stratum II | 16 | 16 | 10 | 32 | 69 | 42 |
| XLOC_021407 | 955.9268673 | -1.007085128 | stratum II | 1 | 32 | 4 | 8 | 87 | 9 |
| XLOC_021945 | 1008.775175 | -1.024452621 | stratum II | 7 | 13 | 10 | 21 | 35 | 14 |
| XLOC_021946 | 1746.129184 | -0.936402654 | stratum II | 1 | 11 | 0 | 1 | 23 | 0 |
| XLOC_021947 | 319.7594828 | -0.950632058 | stratum II | 4 | 5 | 5 | 9 | 25 | 13 |
| XLOC_021408 | 103.3060097 | -0.891361803 | stratum II | 5 | 9 | 5 | 12 | 27 | 26 |
| XLOC_022521 | 7.696138587 | 1.895190892 | stratum II | 4 | 8 | 0 | 5 | 34 | 0 |
| XLOC_021409 | 583.7230128 | -0.176186018 | stratum II | 21 | 3 | 23 | 38 | 15 | 89 |
| XLOC_021410 | 75.677394 | -0.916470636 | stratum II | 0 | 0 | 0 | 0 | 0 | 0 |
| XLOC_021948 | 1515.776352 | -0.740438012 | stratum II | 9 | 15 | 8 | 15 | 48 | 27 |
| XLOC_022522 | 20.5774009 | -0.302597326 | stratum II | 0 | 2 | 0 | 2 | 5 | 0 |
| XLOC_022523 | 3.857353683 | -0.205026023 | stratum II | 0 | 0 | 0 | 0 | 0 | 0 |
| XLOC_022524 | 35.41157434 | -0.531205732 | stratum II | 0 | 0 | 0 | 0 | 0 | 0 |
| XLOC_022525 | 5.082046726 | -1.176318224 | stratum II | 0 | 0 | 0 | 0 | 0 | 0 |
| XLOC_021411 | 3.91052623 | -0.448702062 | stratum II | 0 | 0 | 0 | 0 | 0 | 0 |
| XLOC_021412 | 41.77139669 | -0.812627881 | stratum II | 0 | 0 | 0 | 0 | 6 | 0 |
| XLOC_021413 | 512.4841423 | -0.197929342 | stratum II | 24 | 0 | 22 | 46 | 6 | 50 |
| XLOC_021414 | 181.7846809 | -0.813005752 | stratum II | 0 | 0 | 0 | 0 | 0 | 0 |
| XLOC_021415 | 6311.923999 | -0.969311011 | stratum II | 0 | 0 | 0 | 0 | 0 | 0 |
| XLOC_022526 | 48.10042698 | -0.699293749 | stratum II | 0 | 0 | 0 | 0 | 0 | 0 |
| XLOC_022527 | 48.20462019 | -0.68733038 | stratum II | 0 | 0 | 0 | 0 | 0 | 0 |
| XLOC_022528 | 30.69069078 | -1.154307724 | stratum II | 0 | 0 | 0 | 0 | 0 | 0 |
| XLOC_021416 | 61.93613846 | -1.08559072 | stratum II | 1 | 0 | 0 | 0 | 0 | 0 |
| XLOC_021417 | 1975.426409 | -0.872615564 | stratum II | 6 | 0 | 4 | 13 | 14 | 25 |
| XLOC_022529 | 176.1472455 | -1.148659615 | stratum II | 0 | 0 | 0 | 0 | 0 | 0 |
| XLOC_021950 | 32.23108231 | -0.76545872 | stratum II | 0 | 0 | 0 | 0 | 0 | 0 |
| XLOC_021951 | 1241.348435 | -0.95792567 | stratum II | 0 | 8 | 0 | 0 | 19 | 0 |
| XLOC_021418 | 4298.477405 | -0.514535241 | stratum II | 13 | 1 | 19 | 42 | 3 | 35 |
| XLOC_021419 | 1891.568229 | -0.765680475 | stratum II | 5 | 7 | 13 | 22 | 28 | 48 |
| XLOC_022530 | 33.89262317 | -0.555574804 | stratum II | 0 | 16 | 0 | 0 | 68 | 0 |
| XLOC_021420 | 556.6190165 | -0.984889877 | stratum II | 4 | 6 | 9 | 26 | 20 | 41 |
| XLOC_022531 | 13.92208441 | -0.894157137 | stratum II | 0 | 0 | 0 | 0 | 0 | 0 |
| XLOC_021421 | 653.5937587 | -0.860834426 | stratum II | 0 | 0 | 0 | 0 | 0 | 0 |
| XLOC_021422 | 13.14251956 | -1.12566703 | stratum II | 0 | 0 | 0 | 0 | 0 | 0 |
| XLOC_021952 | 235.6970074 | -0.952242055 | stratum II | 0 | 0 | 0 | 0 | 0 | 0 |
| XLOC_022532 | 17.34720118 | -0.839631995 | stratum II | 0 | 0 | 0 | 0 | 0 | 0 |
| XLOC_022533 | 15.28483925 | -0.682557681 | stratum II | 1 | 0 | 0 | 0 | 0 | 0 |
| XLOC_022534 | 29.29046453 | -0.855429325 | stratum II | 0 | 0 | 0 | 0 | 0 | 0 |
| XLOC_021953 | 1384.699597 | -0.84035365 | stratum II | 0 | 0 | 0 | 0 | 0 | 0 |
| XLOC_021423 | 5.288841301 | -0.389903582 | stratum II | 0 | 0 | 0 | 0 | 0 | 0 |
| XLOC_021955 | 4074.980908 | -1.025527831 | stratum II | 0 | 0 | 0 | 0 | 0 | 0 |
| XLOC_022535 | 25.50614102 | -1.12515612 | stratum II | 0 | 0 | 0 | 0 | 0 | 0 |
| XLOC_022536 | 68.65540967 | -1.035104827 | stratum II | 0 | 0 | 0 | 0 | 0 | 0 |
| XLOC_022537 | 26.37140794 | -0.65807679 | stratum II | 0 | 0 | 0 | 0 | 0 | 0 |
| XLOC_022538 | 37.41225984 | -0.831203598 | stratum II | 0 | 0 | 0 | 0 | 0 | 0 |
| XLOC_022539 | 17.79543186 | -0.956231814 | stratum II | 0 | 1 | 0 | 0 | 1 | 1 |
| XLOC_022540 | 18.55539272 | -0.276007897 | stratum II | 0 | 0 | 0 | 0 | 0 | 0 |
| XLOC_022541 | 14.4813922 | -0.794958669 | stratum II | 0 | 0 | 0 | 0 | 0 | 0 |
| XLOC_022542 | 4.907043643 | -0.942697818 | stratum II | 0 | 0 | 0 | 0 | 0 | 0 |
| XLOC_022543 | 6.570913066 | -0.105217319 | stratum II | 0 | 3 | 0 | 0 | 8 | 0 |
| XLOC_022544 | 7.110489292 | -0.758870168 | stratum II | 0 | 4 | 0 | 0 | 8 | 0 |
| XLOC_021424 | 3700.829951 | -0.762805533 | stratum II | 8 | 0 | 2 | 5 | 0 | 3 |
| XLOC_021425 | 180.1016285 | -0.891966245 | stratum II | 0 | 0 | 0 | 0 | 0 | 0 |
| XLOC_022545 | 5.649749235 | -0.674892128 | stratum II | 0 | 0 | 0 | 0 | 0 | 0 |
| XLOC_021426 | 517.7573925 | -0.925915023 | stratum II | 0 | 0 | 0 | 0 | 0 | 0 |
| XLOC_021427 | 310.2318912 | -0.822158232 | stratum II | 0 | 0 | 0 | 0 | 0 | 0 |
| XLOC_022546 | 4.32677721 | 0.354617974 | stratum II | 0 | 0 | 0 | 0 | 0 | 0 |
| XLOC_021428 | 1062.527061 | -0.981122969 | stratum II | 0 | 0 | 0 | 0 | 0 | 0 |
| XLOC_022547 | 8.88570738 | -0.354045093 | stratum II | 0 | 0 | 0 | 0 | 0 | 0 |
| XLOC_021956 | 3.939685439 | -0.497277783 | stratum II | 0 | 0 | 0 | 0 | 0 | 0 |
| XLOC_022548 | 4.295376193 | -0.456634293 | stratum II | 0 | 0 | 0 | 0 | 0 | 0 |
| XLOC_021957 | 306.6328497 | -0.895022535 | stratum II | 0 | 0 | 0 | 0 | 0 | 0 |
| XLOC_021429 | 1967.967467 | -0.326452088 | stratum II | 10 | 0 | 28 | 33 | 15 | 49 |
| XLOC_021430 | 219.3509343 | -0.816874138 | stratum II | 0 | 5 | 0 | 0 | 10 | 0 |
| XLOC_021958 | 295.9897207 | -1.055508651 | stratum II | 0 | 0 | 0 | 0 | 0 | 0 |
| XLOC_021959 | 84.33564151 | -0.923553806 | stratum II | 0 | 0 | 0 | 0 | 0 | 0 |
| XLOC_021960 | 417.3512068 | -0.872936744 | stratum II | 1 | 0 | 0 | 0 | 0 | 1 |
| XLOC_021431 | 11.19796266 | -0.867979577 | stratum II | 0 | 0 | 0 | 0 | 0 | 0 |
| XLOC_021961 | 3.846240551 | -0.504393041 | stratum II | 0 | 0 | 0 | 0 | 0 | 0 |
| XLOC_021432 | 438.152922 | -0.354101017 | stratum II | 0 | 4 | 0 | 13 | 15 | 7 |
| XLOC_021433 | 106.5151714 | -0.490973922 | stratum II | 14 | 0 | 6 | 30 | 11 | 19 |
| XLOC_021434 | 2016.032137 | -0.927540486 | stratum II | 0 | 0 | 0 | 0 | 4 | 4 |
| XLOC_021435 | 75.53577158 | -1.106771248 | stratum II | 0 | 0 | 0 | 0 | 1 | 0 |
| XLOC_021962 | 1180.003865 | -1.009209026 | stratum II | 0 | 0 | 0 | 0 | 0 | 0 |
| XLOC_021963 | 562.7252989 | -0.900958097 | stratum II | 0 | 0 | 0 | 0 | 0 | 0 |
| XLOC_021966 | 506.3378867 | -1.088737743 | stratum II | 0 | 0 | 0 | 0 | 2 | 0 |
| XLOC_022549 | 33.21336467 | -0.596946828 | stratum II | 0 | 0 | 0 | 0 | 0 | 0 |
| XLOC_021968 | 123.8496409 | -0.93656762 | stratum II | 0 | 0 | 0 | 0 | 0 | 0 |
| XLOC_021436 | 31.49199265 | -1.050729189 | stratum II | 0 | 1 | 1 | 0 | 15 | 3 |
| XLOC_021437 | 161.1297296 | -0.913559837 | stratum II | 0 | 0 | 0 | 4 | 0 | 0 |
| XLOC_021438 | 370.2025487 | -0.787038787 | stratum II | 0 | 0 | 0 | 0 | 0 | 0 |
| XLOC_022550 | 4.187991022 | -0.498258066 | stratum II | 0 | 0 | 0 | 0 | 0 | 0 |
| XLOC_022551 | 10.66758078 | -0.691487214 | stratum II | 0 | 0 | 0 | 0 | 0 | 0 |
| XLOC_022552 | 3.995203454 | -0.992231345 | stratum II | 0 | 0 | 0 | 0 | 0 | 0 |
| XLOC_021970 | 130.9579581 | -1.143393952 | stratum II | 0 | 0 | 0 | 0 | 0 | 0 |
| XLOC_021440 | 141.8769845 | -0.967137815 | stratum II | 0 | 0 | 0 | 0 | 0 | 0 |
| XLOC_021971 | 966.5081571 | -0.886363381 | stratum II | 0 | 0 | 0 | 0 | 0 | 0 |
| XLOC_022553 | 15.00616893 | -0.498688197 | stratum II | 0 | 0 | 1 | 0 | 1 | 0 |
| XLOC_022554 | 145.669952 | -1.019231086 | stratum II | 0 | 0 | 0 | 0 | 0 | 0 |
| XLOC_022555 | 31.28710777 | -0.557061538 | stratum II | 0 | 0 | 0 | 0 | 0 | 0 |
| XLOC_021441 | 4517.1045 | -0.951619711 | stratum II | 0 | 0 | 0 | 0 | 9 | 0 |
| XLOC_021442 | 54.82816124 | -1.076384269 | stratum II | 0 | 0 | 0 | 0 | 0 | 5 |
| XLOC_021443 | 143.8081517 | -0.824392905 | stratum II | 0 | 0 | 0 | 0 | 0 | 1 |
| XLOC_021972 | 473.5130956 | -1.016021836 | stratum II | 0 | 0 | 0 | 0 | 0 | 0 |
| XLOC_021444 | 647.6604021 | -0.90094018 | stratum II | 0 | 0 | 0 | 0 | 0 | 0 |
| XLOC_022556 | 178.8064363 | -0.975790881 | stratum II | 0 | 0 | 0 | 0 | 0 | 0 |
| XLOC_022557 | 28.05376841 | -0.576842499 | stratum II | 0 | 0 | 0 | 0 | 5 | 0 |
| XLOC_022558 | 415.3696415 | -0.933037816 | stratum II | 0 | 0 | 0 | 5 | 0 | 0 |
| XLOC_021973 | 49.68521586 | -0.753471322 | stratum II | 0 | 0 | 0 | 15 | 1 | 5 |
| XLOC_021974 | 1090.027962 | -0.929799252 | stratum II | 0 | 0 | 0 | 0 | 0 | 0 |
| XLOC_021445 | 18.14597522 | -0.689310015 | stratum II | 0 | 0 | 0 | 0 | 4 | 0 |
| XLOC_022559 | 10.58277141 | -0.636824088 | stratum II | 0 | 0 | 0 | 0 | 4 | 0 |
| XLOC_021975 | 746.3520406 | -1.01430481 | stratum II | 0 | 0 | 0 | 0 | 0 | 0 |
| XLOC_022560 | 6.76877791 | -0.398611956 | stratum II | 0 | 0 | 0 | 0 | 0 | 1 |
| XLOC_021976 | 40.2215153 | -0.731950506 | stratum II | 0 | 0 | 0 | 0 | 0 | 0 |
| XLOC_021446 | 71.5552508 | -0.978098286 | stratum II | 0 | 0 | 1 | 0 | 0 | 0 |
| XLOC_021977 | 14.90303965 | -1.312934257 | stratum II | 0 | 0 | 0 | 0 | 0 | 0 |
| XLOC_021447 | 189.7442171 | -0.809788654 | stratum II | 0 | 0 | 0 | 0 | 0 | 0 |
| XLOC_021448 | 178.6056673 | -0.872406432 | stratum II | 0 | 0 | 0 | 0 | 0 | 0 |
| XLOC_021449 | 138.6892878 | -0.93186684 | stratum II | 0 | 0 | 0 | 0 | 0 | 0 |
| XLOC_022561 | 1018.830729 | -1.092554567 | stratum II | 0 | 0 | 0 | 0 | 0 | 0 |
| XLOC_021450 | 191.2507256 | -0.989338567 | stratum II | 0 | 0 | 0 | 0 | 0 | 0 |
| XLOC_021451 | 2838.915802 | -0.964502894 | stratum II | 0 | 0 | 0 | 0 | 1 | 0 |
| XLOC_021452 | 366.9308598 | -0.209026644 | stratum II | 3 | 0 | 3 | 4 | 6 | 8 |
| XLOC_021453 | 1502.198034 | 0.239533783 | stratum II | 1 | 6 | 3 | 5 | 9 | 1 |
| XLOC_021978 | 12.3218773 | -0.615338795 | stratum II | 0 | 1 | 0 | 0 | 0 | 0 |
| XLOC_021979 | 4.469288418 | -0.462415926 | stratum II | 0 | 0 | 1 | 0 | 0 | 1 |
| XLOC_021454 | 57.45211088 | -0.895933589 | stratum II | 0 | 0 | 0 | 0 | 0 | 3 |
| XLOC_021980 | 2862.208572 | -1.001423536 | stratum II | 2 | 0 | 1 | 4 | 0 | 3 |
| XLOC_022562 | 14.20750282 | -0.613465568 | stratum II | 0 | 0 | 0 | 0 | 0 | 0 |
| XLOC_022563 | 28.39580157 | -0.247308784 | stratum II | 0 | 0 | 0 | 0 | 0 | 0 |
| XLOC_021455 | 54.82313531 | -1.052192096 | stratum II | 0 | 0 | 0 | 1 | 2 | 1 |
| XLOC_021981 | 4.304975118 | 0.374231547 | stratum II | 0 | 1 | 2 | 0 | 0 | 5 |
| XLOC_021982 | 87.35237766 | -1.137870325 | stratum II | 0 | 0 | 0 | 0 | 1 | 0 |
| XLOC_021983 | 6.807431409 | -1.329281045 | stratum II | 0 | 0 | 0 | 0 | 0 | 0 |
| XLOC_021456 | 230.2760904 | -1.055296734 | stratum II | 0 | 0 | 0 | 0 | 0 | 1 |
| XLOC_021457 | 83.18285782 | 0.463897936 | stratum II | 0 | 0 | 0 | 0 | 2 | 2 |
| XLOC_021984 | 6.454691231 | -0.672796701 | stratum II | 0 | 0 | 0 | 0 | 2 | 0 |
| XLOC_021985 | 86.03270689 | -0.875787036 | stratum II | 0 | 0 | 0 | 0 | 0 | 0 |
| XLOC_021458 | 247.7336511 | -0.740006305 | stratum II | 0 | 5 | 0 | 0 | 15 | 0 |
| XLOC_021987 | 814.6278079 | -1.048461219 | stratum II | 0 | 0 | 0 | 0 | 0 | 0 |
| XLOC_021988 | 88.5347073 | -0.960560913 | stratum II | 0 | 0 | 0 | 0 | 0 | 0 |
| XLOC_022564 | 46.35671169 | -0.558889564 | stratum II | 0 | 0 | 0 | 0 | 0 | 0 |
| XLOC_022565 | 58.43035684 | -0.810420279 | stratum II | 0 | 0 | 0 | 0 | 0 | 0 |
| XLOC_021459 | 186.9864597 | -0.98406325 | stratum II | 0 | 0 | 0 | 0 | 0 | 0 |
| XLOC_021991 | 529.0630568 | -0.903390947 | stratum II | 9 | 0 | 1 | 7 | 4 | 3 |
| XLOC_022566 | 18.85737819 | -0.965673462 | stratum II | 0 | 0 | 0 | 0 | 5 | 0 |
| XLOC_021992 | 743.4566851 | -0.942831967 | stratum II | 0 | 0 | 0 | 0 | 0 | 0 |
| XLOC_021993 | 135.2807428 | -0.970176021 | stratum II | 0 | 0 | 0 | 0 | 0 | 0 |
| XLOC_021462 | 311.9630099 | -0.882674928 | stratum II | 0 | 0 | 0 | 0 | 14 | 0 |
| XLOC_021463 | 6.293023818 | -0.510544431 | stratum II | 0 | 0 | 0 | 6 | 14 | 2 |
| XLOC_021464 | 695.7752808 | -0.868899924 | stratum II | 0 | 0 | 0 | 0 | 0 | 0 |
| XLOC_022567 | 10.71828993 | -0.747661492 | stratum II | 0 | 0 | 0 | 0 | 0 | 0 |
| XLOC_021466 | 202.2816975 | -1.07740371 | stratum II | 0 | 0 | 2 | 0 | 0 | 5 |
| XLOC_021467 | 183.4565189 | -0.956847767 | stratum II | 0 | 0 | 0 | 0 | 0 | 0 |
| XLOC_021994 | 3.83739325 | -0.671515243 | stratum II | 0 | 0 | 4 | 2 | 2 | 8 |
| XLOC_022568 | 7.212356747 | 0.35163038 | stratum II | 0 | 3 | 0 | 0 | 6 | 0 |
| XLOC_022569 | 3.828163171 | 0.562879281 | stratum II | 3 | 1 | 0 | 5 | 3 | 0 |
| XLOC_021995 | 5.810914833 | -0.688034368 | stratum II | 0 | 0 | 0 | 0 | 0 | 0 |
| XLOC_021996 | 252.8276885 | -0.907785995 | stratum II | 0 | 0 | 0 | 0 | 0 | 0 |
| XLOC_021997 | 5.365002799 | -0.63128338 | stratum II | 0 | 0 | 0 | 0 | 0 | 0 |
| XLOC_021468 | 242.9270842 | -0.793988472 | stratum II | 0 | 0 | 0 | 0 | 0 | 0 |
| XLOC_021469 | 726.69158 | -0.854212522 | stratum II | 0 | 0 | 0 | 0 | 0 | 0 |
| XLOC_021998 | 3.530975398 | -0.828023092 | stratum II | 0 | 0 | 0 | 0 | 0 | 0 |
| XLOC_021999 | 5.152101855 | -0.712424757 | stratum II | 0 | 0 | 0 | 0 | 0 | 0 |
| XLOC_021470 | 20.16834171 | -0.916307201 | stratum II | 0 | 0 | 0 | 0 | 3 | 0 |
| XLOC_021471 | 403.1994022 | -0.835255678 | stratum II | 0 | 0 | 0 | 0 | 3 | 2 |
| XLOC_022570 | 114.9518368 | -0.958429624 | stratum II | 0 | 0 | 0 | 0 | 0 | 0 |
| XLOC_021472 | 59.70538675 | -0.425014517 | stratum II | 0 | 0 | 0 | 0 | 0 | 0 |
| XLOC_021473 | 384.9331637 | -0.86063129 | stratum II | 0 | 6 | 0 | 0 | 20 | 0 |
| XLOC_022000 | 563.0949203 | -0.75257241 | stratum II | 0 | 0 | 0 | 0 | 0 | 0 |
| XLOC_022001 | 6.406482303 | -0.248534723 | stratum II | 0 | 0 | 0 | 0 | 0 | 0 |
| XLOC_021474 | 32.84207872 | -0.984079935 | stratum II | 0 | 0 | 0 | 0 | 0 | 0 |
| XLOC_021475 | 11.51126322 | -0.460587294 | stratum II | 0 | 0 | 0 | 0 | 3 | 1 |
| XLOC_022003 | 20.83688164 | -0.412671297 | stratum II | 0 | 0 | 0 | 3 | 0 | 4 |
| XLOC_022571 | 8.035775394 | 0.160594619 | stratum II | 0 | 0 | 0 | 0 | 0 | 0 |
| XLOC_022572 | 2.539122065 | -0.052661003 | stratum II | 0 | 0 | 0 | 0 | 1 | 0 |
| XLOC_022573 | 4.408555284 | 0.673021485 | stratum II | 0 | 0 | 0 | 0 | 1 | 0 |
| XLOC_021476 | 1068.402932 | -0.842218148 | stratum II | 0 | 0 | 0 | 0 | 0 | 0 |
| XLOC_022576 | 3.739346798 | -0.73652326 | stratum II | 0 | 0 | 0 | 0 | 0 | 0 |
| XLOC_021477 | 10.45442716 | -0.507439931 | stratum II | 0 | 0 | 0 | 0 | 0 | 0 |
| XLOC_022004 | 34.18463706 | -0.066596649 | stratum II | 0 | 0 | 0 | 2 | 0 | 0 |
| XLOC_021479 | 420.2514656 | -0.757003582 | stratum II | 0 | 4 | 0 | 0 | 16 | 0 |
| XLOC_021480 | 396.9392591 | -0.406513111 | stratum II | 16 | 0 | 18 | 32 | 0 | 37 |
| XLOC_022577 | 401.3791556 | -0.968092853 | stratum II | 0 | 0 | 0 | 0 | 0 | 0 |
| XLOC_022578 | 4.228864075 | -1.165714105 | stratum II | 0 | 0 | 0 | 0 | 0 | 0 |
| XLOC_021481 | 22.75146054 | -0.574851505 | stratum II | 0 | 0 | 0 | 0 | 0 | 0 |
| XLOC_021482 | 4.972134222 | -0.636646123 | stratum II | 0 | 0 | 0 | 0 | 0 | 0 |
| XLOC_021483 | 388.2424694 | -0.878919398 | stratum II | 0 | 0 | 0 | 0 | 0 | 0 |
| XLOC_022579 | 11.24654013 | -0.523265058 | stratum II | 0 | 0 | 0 | 0 | 0 | 0 |
| XLOC_022006 | 9.557568158 | -0.674338063 | stratum II | 0 | 2 | 0 | 0 | 4 | 0 |
| XLOC_021484 | 39.42137119 | -0.961900994 | stratum II | 0 | 0 | 0 | 0 | 0 | 0 |
| XLOC_021485 | 15.15893955 | -0.417765795 | stratum II | 0 | 5 | 0 | 0 | 12 | 0 |
| XLOC_022580 | 10.43388323 | -1.100781641 | stratum II | 0 | 1 | 0 | 5 | 2 | 0 |
| XLOC_022007 | 79.44640556 | -1.214436462 | stratum II | 2 | 1 | 0 | 10 | 2 | 1 |
| XLOC_022008 | 28.05340793 | -0.740267951 | stratum II | 0 | 0 | 0 | 0 | 0 | 11 |
| XLOC_022009 | 42.64177877 | -0.741901358 | stratum II | 0 | 3 | 0 | 4 | 1 | 16 |
| XLOC_022010 | 3.508624859 | -0.430185419 | stratum II | 0 | 3 | 0 | 0 | 2 | 0 |
| XLOC_022581 | 10.97201573 | 2.932707897 | stratum II | 0 | 0 | 0 | 0 | 0 | 0 |
| XLOC_022011 | 16.71129578 | -0.789464492 | stratum II | 0 | 0 | 0 | 0 | 0 | 0 |
| XLOC_022012 | 174.7195527 | -0.509413546 | stratum II | 0 | 0 | 0 | 0 | 0 | 0 |
| XLOC_022014 | 104.2192421 | -0.81653309 | stratum II | 0 | 0 | 0 | 0 | 0 | 1 |
| XLOC_022015 | 80.7072442 | -1.053195158 | stratum II | 0 | 0 | 0 | 0 | 1 | 0 |
| XLOC_022016 | 4221.102073 | -0.980160642 | stratum II | 0 | 0 | 0 | 0 | 0 | 0 |
| XLOC_021488 | 1069.575777 | -0.975010053 | stratum II | 0 | 0 | 0 | 0 | 0 | 1 |
| XLOC_021489 | 239.0907669 | -0.799907008 | stratum II | 0 | 0 | 0 | 0 | 0 | 0 |
| XLOC_021490 | 190.1052253 | -0.970604662 | stratum II | 0 | 0 | 0 | 0 | 0 | 0 |
| XLOC_022017 | 29.70108071 | -0.770626464 | stratum II | 0 | 0 | 0 | 0 | 0 | 0 |
| XLOC_022018 | 38.0120618 | -0.799642287 | stratum II | 0 | 0 | 0 | 0 | 0 | 0 |
| XLOC_021492 | 357.9387804 | -0.909678718 | stratum II | 0 | 0 | 0 | 0 | 0 | 0 |
| XLOC_022019 | 457.5751851 | -0.915988327 | stratum II | 0 | 0 | 0 | 0 | 0 | 0 |
| XLOC_021493 | 179.4151299 | -0.815710674 | stratum II | 0 | 0 | 0 | 0 | 0 | 0 |
| XLOC_021494 | 53.8999134 | -0.710763093 | stratum II | 0 | 0 | 0 | 0 | 0 | 0 |
| XLOC_022582 | 8.210416146 | -0.557542352 | stratum II | 0 | 0 | 0 | 0 | 2 | 0 |
| XLOC_021495 | 245.0833968 | -0.847979149 | stratum II | 0 | 0 | 0 | 0 | 0 | 0 |
| XLOC_022020 | 63.66705616 | -1.685244753 | stratum II | 0 | 0 | 0 | 0 | 0 | 0 |
| XLOC_022021 | 8.904173859 | -0.948906176 | stratum II | 0 | 0 | 0 | 0 | 5 | 0 |
| XLOC_021496 | 2068.715948 | -0.948535591 | stratum II | 3 | 1 | 1 | 8 | 0 | 11 |
| XLOC_022022 | 118.5467398 | -0.622490276 | stratum II | 0 | 0 | 0 | 0 | 0 | 0 |
| XLOC_022583 | 17.29889892 | -0.471685733 | stratum II | 0 | 0 | 0 | 0 | 0 | 0 |
| XLOC_021497 | 284.9499315 | -0.964206122 | stratum II | 0 | 0 | 0 | 0 | 0 | 0 |
| XLOC_022023 | 3826.209966 | -0.476197121 | stratum II | 1 | 2 | 1 | 4 | 12 | 1 |
| XLOC_021498 | 450.9131054 | -0.919489835 | stratum II | 0 | 4 | 0 | 9 | 7 | 6 |
| XLOC_022024 | 389.1021073 | -0.933405877 | stratum II | 0 | 0 | 0 | 0 | 0 | 1 |
| XLOC_022025 | 206.3444286 | -0.836218511 | stratum II | 0 | 0 | 0 | 0 | 0 | 0 |
| XLOC_022026 | 370.8379324 | -0.89848759 | stratum II | 0 | 0 | 0 | 0 | 1 | 1 |
| XLOC_022027 | 658.590516 | -0.951978922 | stratum II | 0 | 0 | 0 | 0 | 10 | 0 |
| XLOC_022584 | 1391.534342 | -1.016336162 | stratum II | 0 | 0 | 0 | 1 | 10 | 0 |
| XLOC_022028 | 1855.63529 | -0.77603667 | stratum II | 0 | 0 | 0 | 1 | 1 | 4 |
| XLOC_022585 | 22.32498062 | -0.567554673 | stratum II | 0 | 0 | 0 | 0 | 1 | 0 |
| XLOC_022586 | 25.2157856 | -0.832554475 | stratum II | 3 | 1 | 0 | 0 | 2 | 0 |
| XLOC_021499 | 117.6823438 | -0.857610114 | stratum II | 4 | 2 | 8 | 31 | 14 | 21 |
| XLOC_022029 | 39.49337479 | -0.721520727 | stratum II | 0 | 6 | 0 | 5 | 20 | 5 |
| XLOC_022030 | 562.3156231 | -0.900956308 | stratum II | 0 | 0 | 0 | 0 | 0 | 0 |
| XLOC_022587 | 15.21365341 | -0.501353889 | stratum II | 0 | 0 | 0 | 0 | 0 | 0 |
| XLOC_021501 | 46.21152055 | -0.758564624 | stratum II | 4 | 0 | 3 | 3 | 1 | 4 |
| XLOC_022031 | 2.547487018 | 0.301395121 | stratum II | 1 | 4 | 0 | 2 | 4 | 0 |
| XLOC_021502 | 248.3421457 | -1.002726483 | stratum II | 0 | 0 | 1 | 0 | 0 | 0 |
| XLOC_022588 | 7.921969368 | -0.817904743 | stratum II | 0 | 0 | 0 | 0 | 0 | 0 |
| XLOC_022032 | 1292.083142 | -0.770804042 | stratum II | 3 | 0 | 2 | 19 | 1 | 7 |
| XLOC_022589 | 30.8086816 | -1.088886832 | stratum II | 0 | 0 | 0 | 0 | 3 | 0 |
| XLOC_022033 | 633.1061699 | -1.118969823 | stratum II | 0 | 0 | 0 | 1 | 0 | 3 |
| XLOC_022590 | 7.776410928 | -1.060844617 | stratum II | 0 | 0 | 0 | 0 | 0 | 0 |
| XLOC_022034 | 12.98078015 | -0.712166907 | stratum II | 0 | 0 | 0 | 0 | 0 | 0 |
| XLOC_021503 | 153.9820351 | -1.2053511 | stratum II | 0 | 0 | 0 | 0 | 0 | 4 |
| XLOC_021504 | 784.5092934 | -0.883804184 | stratum II | 0 | 0 | 0 | 0 | 0 | 3 |
| XLOC_021505 | 44.4565157 | -0.446446621 | stratum II | 0 | 0 | 0 | 0 | 0 | 0 |
| XLOC_021506 | 464.1844579 | -0.986146669 | stratum II | 3 | 0 | 0 | 1 | 0 | 0 |
| XLOC_022591 | 11.13040836 | -0.775560497 | stratum II | 0 | 0 | 0 | 0 | 0 | 0 |
| XLOC_022036 | 6200.578176 | -0.933201478 | stratum II | 0 | 3 | 0 | 0 | 2 | 0 |
| XLOC_022037 | 1013.090995 | -0.851931401 | stratum II | 1 | 3 | 1 | 7 | 21 | 1 |
| XLOC_021507 | 113.3163182 | -0.367582908 | stratum II | 6 | 2 | 1 | 25 | 20 | 8 |
| XLOC_022592 | 65.17370392 | -1.077831739 | stratum II | 0 | 0 | 0 | 0 | 0 | 0 |
| XLOC_022038 | 194.8070131 | -0.884537374 | stratum II | 0 | 0 | 1 | 0 | 0 | 1 |
| XLOC_022593 | 10.0611489 | 0.334544485 | stratum II | 0 | 1 | 0 | 1 | 0 | 0 |
| XLOC_022039 | 847.0148262 | -1.046929993 | stratum II | 0 | 0 | 0 | 0 | 0 | 0 |
| XLOC_021508 | 1235.650147 | -0.682783706 | stratum II | 0 | 0 | 0 | 0 | 0 | 0 |
| XLOC_022594 | 26.6042295 | 0.129778927 | stratum II | 0 | 0 | 0 | 0 | 0 | 0 |
| XLOC_021509 | 244.6683179 | -0.905757267 | stratum II | 0 | 4 | 4 | 0 | 8 | 7 |
| XLOC_021510 | 190.9173556 | -0.358898068 | stratum II | 3 | 4 | 2 | 13 | 9 | 2 |
| XLOC_022595 | 8.778425266 | -1.173790558 | stratum II | 0 | 1 | 0 | 0 | 7 | 0 |
| XLOC_022040 | 768.6210665 | -0.919387301 | stratum II | 0 | 0 | 1 | 0 | 0 | 1 |
| XLOC_022596 | 11.37932976 | -0.386295284 | stratum II | 0 | 0 | 0 | 0 | 0 | 0 |
| XLOC_022041 | 197.2261802 | -1.399050443 | stratum II | 0 | 0 | 0 | 0 | 0 | 0 |
| XLOC_022042 | 6.479731023 | -0.65849282 | stratum II | 0 | 0 | 0 | 0 | 0 | 0 |
| XLOC_022043 | 412.6196766 | -0.767430047 | stratum II | 0 | 0 | 1 | 0 | 0 | 0 |
| XLOC_022044 | 8.103649017 | -0.490517698 | stratum II | 0 | 1 | 0 | 0 | 9 | 1 |
| XLOC_021511 | 141.7887388 | -1.186981683 | stratum II | 0 | 0 | 0 | 0 | 0 | 0 |
| XLOC_022045 | 209.7399907 | -0.811629482 | stratum II | 18 | 2 | 6 | 33 | 23 | 18 |
| XLOC_021512 | 533.0505919 | -0.981066388 | stratum II | 0 | 16 | 1 | 13 | 25 | 11 |
| XLOC_022597 | 6.884697038 | -0.480087268 | stratum II | 0 | 0 | 0 | 0 | 0 | 0 |
| XLOC_021513 | 445.4953963 | -0.922520741 | stratum II | 0 | 14 | 0 | 0 | 29 | 0 |
| XLOC_022598 | 4.642766852 | -0.476324512 | stratum II | 0 | 0 | 0 | 0 | 0 | 1 |
| XLOC_021514 | 864.5222537 | -0.272502037 | stratum II | 16 | 13 | 22 | 46 | 50 | 71 |
| XLOC_022046 | 10835.2508 | -0.713434186 | stratum II | 0 | 0 | 0 | 2 | 4 | 0 |
| XLOC_022599 | 18.66764987 | -0.972445949 | stratum II | 0 | 0 | 0 | 0 | 0 | 0 |
| XLOC_021515 | 770.5083816 | -0.925877378 | stratum II | 0 | 0 | 0 | 0 | 0 | 0 |
| XLOC_022047 | 395.9270561 | -0.841754556 | stratum II | 0 | 3 | 0 | 0 | 2 | 0 |
| XLOC_021516 | 1098.22309 | -0.969597997 | stratum II | 0 | 14 | 0 | 0 | 19 | 0 |
| XLOC_022048 | 749.2676233 | -0.416519245 | stratum II | 12 | 1 | 17 | 47 | 8 | 71 |
| XLOC_021517 | 58.44553166 | -0.864111285 | stratum II | 0 | 7 | 0 | 0 | 15 | 0 |
| XLOC_022049 | 3721.85172 | -0.965748971 | stratum II | 0 | 4 | 0 | 0 | 22 | 0 |
| XLOC_022600 | 8.328993156 | -0.943183355 | stratum II | 0 | 0 | 0 | 0 | 3 | 0 |
| XLOC_021518 | 23.67605733 | -0.735402842 | stratum II | 0 | 0 | 0 | 0 | 0 | 0 |
| XLOC_021519 | 293.8881347 | -0.866246676 | stratum II | 0 | 0 | 0 | 0 | 0 | 0 |
| XLOC_021520 | 1659.698913 | -0.907446062 | stratum II | 0 | 0 | 0 | 0 | 0 | 0 |
| XLOC_022601 | 6.130544985 | -0.187754721 | stratum II | 0 | 0 | 0 | 0 | 0 | 0 |
| XLOC_021521 | 1633.974344 | -0.858824985 | stratum II | 0 | 0 | 0 | 0 | 0 | 0 |
| XLOC_022050 | 354.3847736 | -0.86998652 | stratum II | 0 | 0 | 0 | 0 | 0 | 0 |
| XLOC_021522 | 453.0155617 | -0.845244147 | stratum II | 0 | 0 | 0 | 0 | 0 | 0 |
| XLOC_021523 | 526.9681422 | -0.991530293 | stratum II | 0 | 0 | 0 | 0 | 0 | 0 |
| XLOC_021524 | 62.5854839 | -0.558696609 | stratum II | 0 | 0 | 0 | 0 | 0 | 0 |
| XLOC_021525 | 12.37702466 | -0.924069451 | stratum II | 0 | 0 | 0 | 0 | 0 | 0 |
| XLOC_022051 | 1290.080859 | -1.071758316 | stratum II | 0 | 0 | 0 | 0 | 0 | 0 |
| XLOC_021526 | 380.5999294 | -1.010838002 | stratum II | 0 | 0 | 0 | 0 | 0 | 0 |
| XLOC_022052 | 34.72144704 | -0.378432538 | stratum II | 0 | 0 | 0 | 0 | 0 | 0 |
| XLOC_021527 | 20441.87608 | -0.867778806 | stratum II | 0 | 0 | 0 | 0 | 0 | 0 |
| XLOC_022602 | 3.633332169 | 0.355949691 | stratum II | 0 | 0 | 0 | 0 | 0 | 0 |
| XLOC_022054 | 131.4940623 | -0.877838941 | stratum II | 0 | 1 | 1 | 0 | 0 | 0 |
| XLOC_021528 | 762.1569192 | -0.846861963 | stratum II | 0 | 0 | 0 | 0 | 0 | 0 |
| XLOC_022055 | 47.05894274 | -0.952282143 | stratum II | 0 | 0 | 0 | 0 | 0 | 0 |
| XLOC_022056 | 782.2374688 | -0.973958987 | stratum II | 0 | 0 | 0 | 0 | 0 | 1 |
| XLOC_021529 | 144.3073902 | -0.91095503 | stratum II | 0 | 10 | 0 | 0 | 27 | 0 |
| XLOC_022057 | 89.73271215 | -0.756994215 | stratum II | 8 | 5 | 11 | 21 | 24 | 42 |
| XLOC_022058 | 227.7207463 | -0.861128627 | stratum II | 0 | 8 | 0 | 6 | 38 | 0 |
| XLOC_022059 | 5590.410703 | -0.171826068 | stratum II | 10 | 14 | 18 | 36 | 40 | 63 |
| XLOC_022060 | 9852.293269 | -0.883605919 | stratum II | 0 | 0 | 0 | 0 | 0 | 2 |
| XLOC_022061 | 42.04309884 | -0.962994095 | stratum II | 0 | 0 | 0 | 0 | 0 | 0 |
| XLOC_022603 | 452.6274529 | -1.046345657 | stratum II | 0 | 0 | 0 | 0 | 0 | 0 |
| XLOC_022062 | 292.5028944 | -0.923062666 | stratum II | 0 | 1 | 0 | 0 | 4 | 3 |
| XLOC_022063 | 203.6012192 | -0.472986042 | stratum II | 7 | 0 | 29 | 15 | 2 | 70 |
| XLOC_022604 | 10.16983086 | -0.787721914 | stratum II | 0 | 2 | 0 | 0 | 8 | 0 |
| XLOC_022064 | 546.6804876 | -0.94080266 | stratum II | 0 | 4 | 12 | 0 | 11 | 23 |
| XLOC_022065 | 1598.296494 | 0.390012716 | stratum II | 15 | 9 | 47 | 22 | 16 | 78 |
| XLOC_022066 | 1518.729259 | -1.019278849 | stratum II | 0 | 26 | 0 | 0 | 61 | 0 |
| XLOC_021530 | 611.9314672 | -0.478359587 | stratum II | 12 | 9 | 32 | 34 | 23 | 64 |
| XLOC_021531 | 458.0818257 | -0.32745722 | stratum II | 17 | 9 | 20 | 41 | 29 | 50 |
| XLOC_021532 | 43.1080617 | -1.031039417 | stratum II | 0 | 4 | 0 | 13 | 19 | 0 |
| XLOC_022067 | 549.9845543 | -0.82207426 | stratum II | 7 | 0 | 0 | 25 | 0 | 11 |
| XLOC_021533 | 775.5045088 | -0.884075105 | stratum II | 0 | 0 | 0 | 0 | 0 | 0 |
| XLOC_021534 | 6862.629877 | -0.834010289 | stratum II | 3 | 1 | 1 | 3 | 20 | 2 |
| XLOC_021535 | 543.1826777 | -0.293727384 | stratum II | 5 | 11 | 0 | 17 | 26 | 8 |
| XLOC_021536 | 1040.867568 | -0.954157876 | stratum II | 0 | 0 | 0 | 0 | 0 | 0 |
| XLOC_022605 | 60.03786249 | -1.043141955 | stratum II | 0 | 0 | 0 | 0 | 0 | 0 |
| XLOC_022606 | 14.55749005 | -0.549171072 | stratum II | 0 | 0 | 0 | 0 | 0 | 0 |
| XLOC_022607 | 13.14799993 | -0.260942871 | stratum II | 0 | 0 | 0 | 0 | 0 | 0 |
| XLOC_022068 | 552.7374272 | -0.918148328 | stratum II | 1 | 0 | 0 | 0 | 2 | 0 |
| XLOC_021537 | 139.134163 | -0.810279028 | stratum II | 0 | 0 | 1 | 0 | 0 | 5 |
| XLOC_022608 | 7.711323099 | -0.759322936 | stratum II | 0 | 0 | 0 | 0 | 0 | 0 |
| XLOC_022070 | 9.613429492 | -1.043207535 | stratum II | 0 | 0 | 0 | 0 | 0 | 0 |
| XLOC_022071 | 1391.717754 | -0.775908488 | stratum II | 0 | 0 | 0 | 0 | 0 | 0 |
| XLOC_021538 | 2234.700006 | -0.060684828 | stratum II | 5 | 0 | 12 | 13 | 0 | 37 |
| XLOC_022072 | 81.84809085 | -0.724336577 | stratum II | 0 | 0 | 0 | 0 | 0 | 0 |
| XLOC_022073 | 121.8496195 | -0.869877385 | stratum II | 0 | 0 | 0 | 0 | 0 | 0 |
| XLOC_022074 | 14.75734855 | -0.501881036 | stratum II | 0 | 0 | 0 | 0 | 0 | 0 |
| XLOC_022075 | 7.009593977 | -0.466333601 | stratum II | 0 | 0 | 0 | 0 | 0 | 0 |
| XLOC_021539 | 14851.42595 | 0.126260634 | stratum II | 3 | 0 | 6 | 6 | 0 | 20 |
| XLOC_021540 | 157.6385028 | -0.920764929 | stratum II | 0 | 3 | 0 | 0 | 2 | 0 |
| XLOC_022076 | 1493.258042 | -0.985132141 | stratum II | 0 | 0 | 0 | 1 | 0 | 1 |
| XLOC_022609 | 31.48988926 | -0.946161431 | stratum II | 0 | 0 | 0 | 0 | 2 | 0 |
| XLOC_022077 | 7.30265668 | -0.919089141 | stratum II | 0 | 0 | 0 | 0 | 0 | 0 |
| XLOC_021541 | 57.64462962 | -1.793038063 | stratum II | 5 | 0 | 0 | 7 | 5 | 0 |
| XLOC_022610 | 2267.559429 | -0.80663157 | stratum II | 0 | 1 | 0 | 0 | 0 | 0 |
| XLOC_022079 | 5009.204885 | -0.811803271 | stratum II | 0 | 0 | 0 | 0 | 1 | 0 |
| XLOC_022080 | 188.6908213 | -0.975111167 | stratum II | 0 | 1 | 0 | 0 | 10 | 0 |
| XLOC_021542 | 5339.282699 | -1.047735244 | stratum II | 6 | 1 | 6 | 19 | 12 | 45 |
| XLOC_022081 | 715.4377268 | -0.662528209 | stratum II | 3 | 0 | 1 | 12 | 0 | 4 |
| XLOC_021543 | 383.6388889 | -0.98722731 | stratum II | 0 | 0 | 0 | 0 | 0 | 0 |
| XLOC_022082 | 6.292385727 | -0.415762424 | stratum II | 0 | 0 | 0 | 0 | 0 | 0 |
| XLOC_022083 | 1028.468943 | -0.274873072 | stratum II | 2 | 0 | 0 | 8 | 4 | 10 |
| XLOC_022611 | 18.5880046 | -0.757973027 | stratum II | 0 | 0 | 0 | 0 | 8 | 0 |
| XLOC_022084 | 1764.29243 | -0.929994348 | stratum II | 0 | 2 | 0 | 0 | 0 | 0 |
| XLOC_022085 | 155.1055706 | -0.751398457 | stratum II | 0 | 0 | 0 | 0 | 0 | 0 |
| XLOC_021544 | 11.52830795 | -0.572117428 | stratum II | 0 | 0 | 0 | 0 | 0 | 0 |
| XLOC_022612 | 4.837830927 | -0.626139035 | stratum II | 0 | 0 | 0 | 0 | 0 | 0 |
| XLOC_021545 | 21.98877578 | -0.613498323 | stratum II | 0 | 10 | 0 | 0 | 15 | 0 |
| XLOC_022086 | 3288.341089 | -0.647493105 | stratum II | 34 | 5 | 28 | 67 | 8 | 116 |
| XLOC_022613 | 110.4940988 | -0.679591345 | stratum II | 0 | 8 | 0 | 0 | 13 | 0 |
| XLOC_022614 | 48.06769685 | -0.660406397 | stratum II | 0 | 11 | 0 | 0 | 57 | 0 |
| XLOC_022615 | 6.652867475 | -0.536200026 | stratum II | 0 | 0 | 0 | 0 | 0 | 0 |
| XLOC_022087 | 65.98521327 | -0.921500634 | stratum II | 0 | 0 | 0 | 0 | 0 | 0 |
| XLOC_021546 | 5.989529604 | -0.687795231 | stratum II | 0 | 0 | 0 | 0 | 0 | 0 |
| XLOC_022088 | 659.1986087 | -0.893581274 | stratum II | 0 | 0 | 0 | 0 | 0 | 0 |
| XLOC_021547 | 704.5552578 | -0.605616917 | stratum II | 11 | 0 | 6 | 29 | 1 | 22 |
| XLOC_022616 | 1208.002371 | -0.973510131 | stratum II | 0 | 6 | 0 | 12 | 12 | 0 |
| XLOC_022089 | 1338.120952 | -0.817134987 | stratum II | 0 | 0 | 0 | 0 | 0 | 0 |
| XLOC_022617 | 60.56280319 | -0.874407147 | stratum II | 0 | 0 | 0 | 0 | 0 | 0 |
| XLOC_022090 | 492.2171214 | -1.050417544 | stratum II | 0 | 0 | 0 | 0 | 0 | 0 |
| XLOC_021548 | 1604.33045 | -0.623394043 | stratum II | 47 | 1 | 5 | 93 | 6 | 27 |
| XLOC_021549 | 219.0649132 | -0.561424933 | stratum II | 0 | 0 | 0 | 0 | 0 | 0 |
| XLOC_021550 | 3810.423114 | -1.160324872 | stratum II | 0 | 0 | 0 | 0 | 0 | 0 |
| XLOC_021551 | 584.7998368 | -0.902290647 | stratum II | 0 | 0 | 0 | 0 | 0 | 0 |
| XLOC_021552 | 97.39699374 | -0.937933874 | stratum II | 0 | 0 | 0 | 0 | 0 | 0 |
| XLOC_021553 | 5306.309324 | -0.980143139 | stratum II | 0 | 0 | 0 | 0 | 0 | 0 |
| XLOC_021554 | 288.7894028 | -1.010210476 | stratum II | 0 | 0 | 0 | 0 | 0 | 0 |
| XLOC_022091 | 3453.600193 | -1.087550163 | stratum II | 0 | 0 | 0 | 0 | 0 | 0 |
| XLOC_022618 | 33.92028169 | -1.303809486 | stratum II | 0 | 0 | 0 | 0 | 0 | 0 |
| XLOC_021555 | 5.425432093 | -0.399867665 | stratum II | 0 | 0 | 0 | 0 | 0 | 0 |
| XLOC_021556 | 198.8466553 | -1.201291604 | stratum II | 0 | 12 | 0 | 0 | 27 | 0 |
| XLOC_021557 | 3388.585666 | -0.492627355 | stratum II | 26 | 1 | 9 | 36 | 7 | 25 |
| XLOC_022092 | 35.22067693 | -0.694455976 | stratum II | 0 | 1 | 0 | 0 | 0 | 0 |
| XLOC_022093 | 133.3440147 | -0.719283075 | stratum II | 0 | 0 | 0 | 0 | 0 | 0 |
| XLOC_021558 | 414.6370465 | -1.068760477 | stratum II | 0 | 0 | 0 | 0 | 2 | 0 |
| XLOC_022094 | 168.2630771 | -0.999334286 | stratum II | 0 | 0 | 0 | 0 | 0 | 0 |
| XLOC_022095 | 1808.408422 | -1.012832316 | stratum II | 0 | 0 | 0 | 0 | 0 | 0 |
| XLOC_021559 | 1223.661265 | -0.932283941 | stratum II | 0 | 0 | 0 | 0 | 0 | 0 |
| XLOC_022619 | 6.960180657 | -0.454664037 | stratum II | 0 | 0 | 0 | 0 | 0 | 0 |
| XLOC_021560 | 148.4767731 | -0.907333715 | stratum II | 0 | 0 | 0 | 0 | 1 | 0 |
| XLOC_022097 | 6778.175993 | -0.983421822 | stratum II | 0 | 0 | 0 | 0 | 0 | 0 |
| XLOC_022620 | 38.98210213 | -0.790689531 | stratum II | 0 | 0 | 0 | 0 | 0 | 0 |
| XLOC_022098 | 546.4045953 | -0.986678591 | stratum II | 0 | 0 | 0 | 0 | 0 | 0 |
| XLOC_021561 | 307.1688107 | -0.86350718 | stratum II | 0 | 0 | 0 | 0 | 0 | 0 |
| XLOC_021562 | 42.02995764 | -0.531030721 | stratum II | 0 | 0 | 0 | 0 | 0 | 0 |
| XLOC_022621 | 10.49928916 | -0.549469183 | stratum II | 0 | 0 | 0 | 0 | 0 | 0 |
| XLOC_022099 | 24.92775448 | -0.520739925 | stratum II | 0 | 4 | 0 | 0 | 7 | 0 |
| XLOC_022100 | 171.1423093 | -0.881769276 | stratum II | 0 | 0 | 0 | 0 | 0 | 0 |
| XLOC_021563 | 300.9096523 | -0.789057419 | stratum II | 0 | 0 | 0 | 0 | 0 | 0 |
| XLOC_022101 | 95.02815796 | -0.925828713 | stratum II | 0 | 0 | 0 | 0 | 0 | 0 |
| XLOC_022622 | 30.4677065 | -0.73475921 | stratum II | 0 | 0 | 0 | 0 | 0 | 0 |
| XLOC_022102 | 187.5305356 | -1.016947213 | stratum II | 0 | 0 | 0 | 0 | 0 | 0 |
| XLOC_021565 | 11.30155062 | -0.92008752 | stratum II | 0 | 0 | 0 | 0 | 0 | 0 |
| XLOC_021566 | 249.9237505 | -0.883768319 | stratum II | 0 | 0 | 0 | 1 | 0 | 1 |
| XLOC_021567 | 364.1172316 | -0.950321155 | stratum II | 0 | 0 | 0 | 0 | 1 | 0 |
| XLOC_021568 | 26.68720462 | -0.190426918 | stratum II | 0 | 4 | 0 | 0 | 18 | 0 |
| XLOC_022104 | 353.1398964 | -0.956907721 | stratum II | 0 | 6 | 0 | 0 | 7 | 0 |
| XLOC_021569 | 17.92417048 | -0.78143812 | stratum II | 0 | 0 | 0 | 0 | 0 | 0 |
| XLOC_022105 | 373.6156124 | -0.794042177 | stratum II | 0 | 0 | 0 | 0 | 0 | 0 |
| XLOC_022106 | 285.2369473 | -1.143996952 | stratum II | 0 | 0 | 0 | 0 | 0 | 0 |
| XLOC_022107 | 984.3644991 | -0.939848035 | stratum II | 0 | 0 | 0 | 0 | 0 | 0 |
| XLOC_022108 | 181.0064033 | -0.969831055 | stratum II | 0 | 0 | 0 | 0 | 0 | 3 |
| XLOC_021570 | 35.87922642 | -0.863427688 | stratum II | 0 | 0 | 1 | 0 | 0 | 0 |
| XLOC_021571 | 478.8920752 | -0.93265125 | stratum II | 0 | 0 | 0 | 0 | 0 | 0 |
| XLOC_022109 | 3905.240134 | -1.023599524 | stratum II | 0 | 0 | 0 | 0 | 6 | 0 |
| XLOC_022623 | 48.43437627 | -0.904443648 | stratum II | 0 | 0 | 0 | 0 | 0 | 0 |
| XLOC_022624 | 5.662731722 | -0.436267346 | stratum II | 0 | 0 | 0 | 6 | 0 | 0 |
| XLOC_022625 | 6.838177407 | -0.510204528 | stratum II | 0 | 0 | 0 | 0 | 6 | 0 |
| XLOC_021572 | 959.6682034 | -1.164333778 | stratum II | 7 | 1 | 15 | 18 | 11 | 71 |
| XLOC_022626 | 29.84767964 | -0.811643128 | stratum II | 0 | 0 | 0 | 0 | 0 | 0 |
| XLOC_022627 | 24.07116818 | -0.940288265 | stratum II | 0 | 0 | 0 | 0 | 5 | 0 |
| XLOC_022628 | 8.55499075 | -0.484743188 | stratum II | 0 | 0 | 0 | 0 | 6 | 0 |
| XLOC_022629 | 4713.823328 | -0.936972076 | stratum II | 7 | 0 | 0 | 31 | 15 | 0 |
| XLOC_022110 | 16.66314284 | -0.870181523 | stratum II | 0 | 0 | 0 | 0 | 0 | 0 |
| XLOC_022630 | 23.61407259 | -1.220735747 | stratum II | 0 | 0 | 0 | 0 | 0 | 0 |
| XLOC_021573 | 1042.319616 | -0.945022761 | stratum II | 0 | 0 | 0 | 0 | 0 | 0 |
| XLOC_022111 | 246.8046337 | -0.998533978 | stratum II | 0 | 2 | 0 | 0 | 0 | 0 |
| XLOC_021574 | 1427.750922 | -0.183076987 | stratum II | 4 | 4 | 0 | 10 | 10 | 0 |
| XLOC_021575 | 692.4469937 | -1.020581006 | stratum II | 0 | 0 | 0 | 0 | 0 | 0 |
| XLOC_022112 | 15.75662439 | -0.753730273 | stratum II | 0 | 0 | 0 | 0 | 0 | 0 |
| XLOC_021576 | 5.112237757 | -0.636462454 | stratum II | 0 | 0 | 0 | 0 | 0 | 0 |
| XLOC_021578 | 420.1720827 | -0.893644957 | stratum II | 0 | 0 | 0 | 0 | 0 | 0 |
| XLOC_022113 | 423.5413381 | -1.031426138 | stratum II | 0 | 5 | 0 | 0 | 15 | 0 |
| XLOC_021579 | 708.5090055 | -1.297136348 | stratum II | 0 | 0 | 30 | 0 | 0 | 33 |
| XLOC_022114 | 1047.542312 | -0.996798385 | stratum II | 0 | 7 | 0 | 4 | 16 | 0 |
| XLOC_021580 | 40.37511092 | -0.619959751 | stratum II | 0 | 0 | 0 | 0 | 0 | 0 |
| XLOC_022115 | 500.2254209 | -0.673266199 | stratum II | 0 | 1 | 0 | 0 | 0 | 0 |
| XLOC_022116 | 382.6855236 | -0.839789861 | stratum II | 0 | 0 | 0 | 0 | 1 | 0 |
| XLOC_022117 | 588.3346519 | -0.828565124 | stratum II | 6 | 1 | 11 | 24 | 5 | 18 |
| XLOC_022118 | 90.17185595 | -0.757358424 | stratum II | 0 | 0 | 0 | 0 | 0 | 0 |
| XLOC_022631 | 18.68537188 | -0.756121722 | stratum II | 0 | 0 | 0 | 0 | 2 | 0 |
| XLOC_022119 | 26.73859444 | -0.674668887 | stratum II | 0 | 0 | 0 | 0 | 0 | 0 |
| XLOC_022632 | 6.075401129 | -0.069848408 | stratum II | 0 | 0 | 0 | 0 | 0 | 0 |
| XLOC_022120 | 128.8044877 | -0.943555055 | stratum II | 0 | 0 | 0 | 0 | 0 | 0 |
| XLOC_022121 | 94.76052475 | -1.032461706 | stratum II | 0 | 0 | 0 | 0 | 2 | 0 |
| XLOC_021581 | 510.0245885 | -0.955133921 | stratum II | 0 | 0 | 0 | 0 | 0 | 0 |
| XLOC_022122 | 357.6672514 | -0.994152155 | stratum II | 0 | 0 | 0 | 0 | 0 | 0 |
| XLOC_022123 | 205.855222 | -0.579001318 | stratum II | 1 | 2 | 3 | 19 | 8 | 11 |
| XLOC_021583 | 2.063685559 | -0.215121077 | stratum II | 0 | 0 | 0 | 1 | 4 | 0 |
| XLOC_022124 | 147.7402289 | -0.875897515 | stratum II | 2 | 15 | 4 | 14 | 51 | 11 |
| XLOC_021584 | 15006.51158 | -0.31499753 | stratum II | 6 | 6 | 13 | 10 | 25 | 48 |
| XLOC_021585 | 4.423627369 | -1.135643285 | stratum II | 0 | 1 | 0 | 0 | 5 | 0 |
| XLOC_021586 | 2.136247979 | -0.610517724 | stratum II | 0 | 0 | 0 | 0 | 6 | 0 |
| XLOC_021587 | 5.286486845 | -0.768540641 | stratum II | 0 | 0 | 0 | 0 | 3 | 0 |
| XLOC_021588 | 5.434372195 | -0.792790583 | stratum II | 0 | 0 | 0 | 0 | 3 | 0 |
| XLOC_021589 | 20.79696077 | -1.242877802 | stratum II | 0 | 0 | 0 | 0 | 3 | 0 |
| XLOC_021590 | 13.99420503 | -0.655837207 | stratum II | 0 | 0 | 0 | 0 | 3 | 0 |
| XLOC_021591 | 26.62150554 | -0.443864105 | stratum II | 0 | 0 | 0 | 0 | 3 | 0 |
| XLOC_022125 | 3192.808878 | -0.990086619 | stratum II | 0 | 0 | 0 | 3 | 3 | 0 |
| XLOC_021592 | 47.27827367 | -0.41689556 | stratum II | 0 | 0 | 0 | 0 | 1 | 0 |
| XLOC_021593 | 1087.352435 | -0.886529494 | stratum II | 0 | 0 | 0 | 0 | 0 | 0 |
| XLOC_021594 | 324.438899 | -0.962399783 | stratum II | 0 | 0 | 0 | 0 | 0 | 0 |
| XLOC_022126 | 4.42358124 | -0.499466015 | stratum II | 0 | 0 | 0 | 0 | 0 | 0 |
| XLOC_021595 | 1223.597168 | -0.99325874 | stratum II | 1 | 0 | 1 | 4 | 0 | 4 |
| XLOC_022127 | 182.410341 | -1.255748954 | stratum II | 0 | 0 | 0 | 0 | 0 | 0 |
| XLOC_021596 | 164.8507849 | -0.895770919 | stratum II | 0 | 1 | 0 | 0 | 0 | 0 |
| XLOC_021597 | 42.62239126 | -1.048094064 | stratum II | 1 | 0 | 0 | 3 | 4 | 0 |
| XLOC_022128 | 937.968595 | -0.721125038 | stratum II | 10 | 7 | 1 | 26 | 19 | 10 |
| XLOC_022633 | 26.61326964 | -0.698991317 | stratum II | 7 | 3 | 0 | 14 | 16 | 0 |
| XLOC_021598 | 677.3510776 | -0.959762947 | stratum II | 0 | 0 | 0 | 0 | 2 | 0 |
| XLOC_021599 | 10.82378059 | -0.521050209 | stratum II | 0 | 0 | 0 | 0 | 0 | 0 |
| XLOC_022635 | 6.665980506 | -0.115675437 | stratum II | 0 | 0 | 0 | 0 | 0 | 0 |
| XLOC_021600 | 409.2672229 | -0.93455974 | stratum II | 0 | 5 | 0 | 0 | 10 | 0 |
| XLOC_021601 | 21.02993719 | -0.36697918 | stratum II | 0 | 1 | 0 | 0 | 0 | 0 |
| XLOC_021602 | 1731.082377 | -0.924592464 | stratum II | 0 | 0 | 1 | 0 | 11 | 21 |
| XLOC_022129 | 3053.634523 | -0.929144662 | stratum II | 0 | 1 | 0 | 2 | 3 | 0 |
| XLOC_021603 | 36.98370789 | -0.801223567 | stratum II | 1 | 1 | 0 | 3 | 1 | 1 |
| XLOC_021604 | 490.5773724 | -0.932009276 | stratum II | 0 | 0 | 3 | 0 | 0 | 3 |
| XLOC_022130 | 495.9507318 | -1.073031641 | stratum II | 0 | 0 | 0 | 0 | 0 | 0 |
| XLOC_022131 | 16.84451965 | -0.711620189 | stratum II | 0 | 0 | 0 | 0 | 0 | 0 |
| XLOC_022132 | 26.35678242 | -0.585253397 | stratum II | 0 | 0 | 0 | 0 | 0 | 0 |
| XLOC_021606 | 303.4413383 | -0.916898634 | stratum II | 0 | 0 | 0 | 0 | 0 | 0 |
| XLOC_021607 | 98.88376909 | -0.8239762 | stratum II | 0 | 0 | 0 | 0 | 0 | 0 |
| XLOC_022133 | 28.25806296 | -1.040587508 | stratum II | 0 | 0 | 0 | 0 | 0 | 0 |
| XLOC_022134 | 16.58340192 | -1.058821431 | stratum II | 0 | 0 | 0 | 0 | 0 | 0 |
| XLOC_022636 | 7.992053178 | -0.794202556 | stratum II | 0 | 0 | 0 | 0 | 0 | 0 |
| XLOC_021608 | 47.8916609 | -0.773488962 | stratum II | 0 | 0 | 0 | 0 | 0 | 0 |
| XLOC_022637 | 29.14300627 | -0.689996789 | stratum II | 0 | 0 | 1 | 0 | 1 | 0 |
| XLOC_022135 | 477.0591776 | -0.884183709 | stratum II | 0 | 0 | 0 | 0 | 0 | 0 |
| XLOC_021609 | 6.749516363 | -0.321251095 | stratum II | 0 | 0 | 0 | 0 | 0 | 0 |
| XLOC_022638 | 14.69720986 | -0.712902153 | stratum II | 0 | 0 | 0 | 0 | 0 | 0 |
| XLOC_022136 | 266.0865922 | -0.954538259 | stratum II | 0 | 0 | 0 | 0 | 0 | 0 |
| XLOC_022639 | 6.021833369 | -0.368911639 | stratum II | 0 | 0 | 0 | 0 | 0 | 0 |
| XLOC_022137 | 5553.09481 | -0.766136338 | stratum II | 20 | 4 | 43 | 62 | 26 | 121 |
| XLOC_022138 | 2238.113694 | -0.247079009 | stratum II | 25 | 5 | 19 | 51 | 3 | 40 |
| XLOC_022139 | 576.9220236 | -0.722066409 | stratum II | 2 | 16 | 1 | 3 | 8 | 1 |
| XLOC_021610 | 36.060357 | -0.925573346 | stratum II | 0 | 0 | 0 | 0 | 0 | 0 |
| XLOC_022140 | 234.5479161 | -0.793753064 | stratum II | 0 | 0 | 0 | 0 | 0 | 0 |
| XLOC_022640 | 34.13674021 | -0.479583618 | stratum II | 0 | 0 | 0 | 0 | 0 | 0 |
| XLOC_022141 | 888.2482067 | -1.089401414 | stratum II | 4 | 7 | 0 | 1 | 6 | 0 |
| XLOC_022142 | 929.2239041 | -0.836087944 | stratum II | 5 | 20 | 3 | 11 | 35 | 5 |
| XLOC_021612 | 239.708668 | 0.347050241 | stratum II | 14 | 11 | 13 | 18 | 63 | 31 |
| XLOC_021613 | 32.76481916 | -0.774983293 | stratum II | 8 | 24 | 0 | 28 | 99 | 7 |
| XLOC_022143 | 1622.489263 | -0.890129673 | stratum II | 8 | 26 | 8 | 34 | 96 | 41 |
| XLOC_022144 | 1238.658301 | -0.825066958 | stratum II | 5 | 9 | 3 | 15 | 27 | 11 |
| XLOC_021617 | 1050.467676 | -0.798748105 | stratum II | 19 | 0 | 7 | 62 | 4 | 10 |
| XLOC_022145 | 530.4083403 | -0.038092895 | stratum II | 10 | 8 | 14 | 32 | 32 | 53 |
| XLOC_022146 | 68.12509703 | -1.076956179 | stratum II | 0 | 0 | 0 | 0 | 0 | 0 |
| XLOC_022641 | 7.236414074 | -0.656636311 | stratum II | 0 | 0 | 0 | 0 | 0 | 0 |
| XLOC_021618 | 3.369589515 | -0.348120793 | stratum II | 0 | 0 | 0 | 0 | 0 | 0 |
| XLOC_021619 | 10.72054898 | -0.294660059 | stratum II | 0 | 0 | 0 | 0 | 0 | 0 |
| XLOC_021621 | 148.6989705 | -0.698968876 | stratum II | 0 | 0 | 0 | 0 | 0 | 0 |
| XLOC_021622 | 3.253293984 | -0.581136194 | stratum II | 0 | 0 | 0 | 0 | 1 | 0 |
| XLOC_022147 | 1061.61795 | -0.86921159 | stratum II | 0 | 0 | 0 | 0 | 0 | 0 |
| XLOC_022148 | 420.7584888 | -0.989137365 | stratum II | 0 | 0 | 0 | 0 | 0 | 0 |
| XLOC_021623 | 932.6016543 | -1.097155857 | stratum II | 0 | 0 | 0 | 0 | 0 | 0 |
| XLOC_021624 | 628.9079634 | -0.75416341 | stratum II | 0 | 0 | 0 | 0 | 0 | 0 |
| XLOC_022149 | 191.0166512 | -0.689558631 | stratum II | 0 | 0 | 0 | 0 | 0 | 0 |
| XLOC_021625 | 10421.4583 | -0.879294506 | stratum II | 0 | 0 | 0 | 0 | 0 | 0 |
| XLOC_022642 | 6.02483662 | -0.108545086 | stratum II | 0 | 0 | 0 | 0 | 0 | 0 |
| XLOC_021626 | 370.6417915 | -0.911264729 | stratum II | 0 | 0 | 0 | 0 | 0 | 0 |
| XLOC_022643 | 14.5669936 | -0.829440501 | stratum II | 0 | 0 | 0 | 0 | 0 | 0 |
| XLOC_022150 | 517.6425471 | -0.879803867 | stratum II | 0 | 3 | 0 | 0 | 14 | 0 |
| XLOC_021627 | 301.7669627 | -0.928866409 | stratum II | 0 | 0 | 3 | 1 | 0 | 18 |
| XLOC_021628 | 168.2460524 | -1.080302687 | stratum II | 0 | 0 | 0 | 0 | 0 | 0 |
| XLOC_022151 | 81.80367552 | -1.051729707 | stratum II | 0 | 0 | 0 | 0 | 0 | 5 |
| XLOC_022644 | 36.85682595 | -0.667526419 | stratum II | 0 | 0 | 0 | 0 | 0 | 0 |
| XLOC_022152 | 224.8692268 | -1.018005536 | stratum II | 0 | 0 | 0 | 0 | 2 | 0 |
| XLOC_021629 | 23.01084112 | -0.833625444 | stratum II | 0 | 0 | 0 | 0 | 0 | 0 |
| XLOC_022645 | 15.4942106 | -0.670673392 | stratum II | 0 | 0 | 0 | 0 | 0 | 0 |
| XLOC_022646 | 20.07341138 | 0.31210802 | stratum II | 0 | 0 | 0 | 0 | 0 | 0 |
| XLOC_021630 | 103.620608 | -0.875008986 | stratum II | 0 | 2 | 0 | 0 | 5 | 0 |
| XLOC_022153 | 243.1628272 | -0.789171455 | stratum II | 2 | 0 | 0 | 2 | 0 | 4 |
| XLOC_021631 | 309.5426224 | -0.916306513 | stratum II | 0 | 0 | 0 | 0 | 0 | 0 |
| XLOC_022647 | 3.90661858 | -0.517168618 | stratum II | 0 | 0 | 0 | 0 | 0 | 0 |
| XLOC_022648 | 8.746385522 | -0.418177849 | stratum II | 0 | 0 | 0 | 0 | 0 | 0 |
| XLOC_022154 | 6267.856459 | -0.831235207 | stratum II | 0 | 0 | 0 | 0 | 1 | 0 |
| XLOC_021632 | 13.37336566 | -0.452873805 | stratum II | 0 | 0 | 0 | 0 | 0 | 0 |
| XLOC_022156 | 9.380493754 | -0.397979949 | stratum II | 0 | 0 | 0 | 0 | 13 | 0 |
| XLOC_021633 | 686.7333238 | -0.893515918 | stratum II | 0 | 0 | 0 | 0 | 0 | 0 |
| XLOC_022157 | 897.5625109 | -0.884200158 | stratum II | 0 | 0 | 0 | 1 | 0 | 0 |
| XLOC_022649 | 14.70948279 | -0.709114382 | stratum II | 0 | 0 | 0 | 0 | 0 | 0 |
| XLOC_021634 | 642.3193517 | -0.928785379 | stratum II | 0 | 0 | 0 | 0 | 0 | 0 |
| XLOC_022158 | 445.0985426 | -0.872412001 | stratum II | 0 | 1 | 0 | 0 | 0 | 0 |
| XLOC_022159 | 2678.110616 | -1.004052898 | stratum II | 0 | 0 | 0 | 0 | 0 | 0 |
| XLOC_022650 | 41.16579372 | -0.183943395 | stratum II | 0 | 0 | 0 | 0 | 0 | 0 |
| XLOC_022651 | 4.478453878 | -0.57010991 | stratum II | 0 | 0 | 0 | 0 | 0 | 0 |
| XLOC_022652 | 16.50351657 | -0.258673662 | stratum II | 0 | 0 | 0 | 0 | 0 | 0 |
| XLOC_022653 | 12.90319435 | -0.382172246 | stratum II | 0 | 0 | 0 | 0 | 0 | 0 |
| XLOC_022654 | 61.85498423 | -0.721691479 | stratum II | 0 | 0 | 0 | 0 | 0 | 0 |
| XLOC_022655 | 127.4514846 | -0.681942699 | stratum II | 0 | 0 | 0 | 0 | 0 | 0 |
| XLOC_022656 | 3.952567894 | -0.463227679 | stratum II | 0 | 0 | 0 | 0 | 0 | 0 |
| XLOC_021638 | 582.4523893 | -0.910398232 | stratum II | 0 | 0 | 0 | 0 | 0 | 0 |
| XLOC_022657 | 6.910089706 | -1.027493831 | stratum II | 0 | 0 | 0 | 0 | 0 | 0 |
| XLOC_022160 | 146.2585776 | -0.837728132 | stratum II | 0 | 0 | 0 | 0 | 0 | 0 |
| XLOC_022161 | 1611.352674 | -0.954322361 | stratum II | 0 | 0 | 0 | 0 | 0 | 0 |
| XLOC_022162 | 12.32713776 | -1.006568842 | stratum II | 0 | 0 | 0 | 0 | 0 | 0 |
| XLOC_022658 | 7.899967677 | -0.401484693 | stratum II | 0 | 0 | 0 | 0 | 0 | 0 |
| XLOC_022163 | 943.2473478 | -1.016858203 | stratum II | 0 | 0 | 0 | 0 | 0 | 0 |
| XLOC_022659 | 28.9615608 | -1.000831769 | stratum II | 0 | 0 | 0 | 0 | 0 | 0 |
| XLOC_021640 | 1129.886005 | -0.612540351 | stratum II | 7 | 2 | 2 | 13 | 6 | 11 |
| XLOC_022660 | 15.47692076 | -0.800575125 | stratum II | 0 | 2 | 0 | 0 | 6 | 0 |
| XLOC_022661 | 54.19768105 | -1.034765251 | stratum II | 0 | 0 | 0 | 0 | 0 | 0 |
| XLOC_022164 | 1385.133177 | -0.9316247 | stratum II | 0 | 0 | 0 | 1 | 0 | 0 |
| XLOC_021641 | 834.8895796 | -0.989658362 | stratum II | 0 | 0 | 0 | 0 | 0 | 0 |
| XLOC_021642 | 43.2943808 | -0.640135523 | stratum II | 0 | 0 | 0 | 0 | 0 | 0 |
| XLOC_022662 | 8.45236986 | -0.724634283 | stratum II | 0 | 0 | 0 | 0 | 0 | 0 |
| XLOC_022166 | 662.1266753 | -0.870958536 | stratum II | 2 | 0 | 0 | 2 | 0 | 0 |
| XLOC_022167 | 252.4485593 | -1.056867867 | stratum II | 0 | 1 | 0 | 0 | 4 | 0 |
| XLOC_022663 | 13.96293581 | -0.914255243 | stratum II | 0 | 0 | 0 | 0 | 0 | 0 |
| XLOC_022664 | 7.21234656 | -1.263254381 | stratum II | 0 | 0 | 0 | 0 | 0 | 0 |
| XLOC_022168 | 325.2905373 | -0.763391778 | stratum II | 0 | 0 | 0 | 0 | 0 | 0 |
| XLOC_022665 | 12.93652276 | -1.029623378 | stratum II | 0 | 0 | 0 | 0 | 0 | 0 |
| XLOC_022666 | 22.53824654 | -0.519502543 | stratum II | 0 | 0 | 2 | 0 | 0 | 0 |
| XLOC_021644 | 50.27641137 | -0.507262993 | stratum II | 0 | 0 | 0 | 0 | 0 | 0 |
| XLOC_022667 | 232.3034264 | -1.04122159 | stratum II | 0 | 1 | 0 | 0 | 1 | 0 |
| XLOC_022169 | 124.9233071 | -0.964918807 | stratum II | 0 | 0 | 0 | 0 | 0 | 0 |
| XLOC_022170 | 984.9236837 | -0.925631832 | stratum II | 0 | 0 | 0 | 0 | 0 | 0 |
| XLOC_021645 | 59.85214169 | -0.991369279 | stratum II | 0 | 0 | 0 | 0 | 0 | 0 |
| XLOC_021646 | 1725.25157 | -0.997641817 | stratum II | 0 | 0 | 0 | 0 | 0 | 0 |
| XLOC_021647 | 1866.047459 | -0.911925335 | stratum II | 0 | 0 | 0 | 0 | 0 | 0 |
| XLOC_021648 | 28.21888729 | -0.992583626 | stratum II | 0 | 0 | 0 | 0 | 0 | 0 |
| XLOC_022171 | 1579.885229 | -1.035759028 | stratum II | 0 | 0 | 0 | 0 | 0 | 0 |
| XLOC_022668 | 84.47222617 | -0.840214005 | stratum II | 0 | 0 | 0 | 0 | 0 | 0 |
| XLOC_022669 | 43.59918848 | -0.793567185 | stratum II | 0 | 0 | 0 | 0 | 0 | 0 |
| XLOC_022172 | 142.5563039 | -1.117067567 | stratum II | 0 | 0 | 0 | 0 | 0 | 0 |
| XLOC_021649 | 978.0739303 | -0.961813305 | stratum II | 0 | 0 | 0 | 0 | 0 | 0 |
| XLOC_022173 | 7.614931533 | -0.643621776 | stratum II | 0 | 0 | 0 | 0 | 0 | 0 |
| XLOC_022670 | 7.165586768 | -0.777123654 | stratum II | 0 | 0 | 0 | 0 | 0 | 0 |
| XLOC_021650 | 577.9731415 | -0.814869204 | stratum II | 0 | 0 | 0 | 0 | 0 | 0 |
| XLOC_022671 | 23.34638448 | -0.692733689 | stratum II | 0 | 1 | 0 | 0 | 0 | 0 |
| XLOC_022672 | 132.5391101 | -0.935837866 | stratum II | 0 | 0 | 0 | 0 | 0 | 0 |
| XLOC_022673 | 127.5738514 | -0.979508163 | stratum II | 0 | 0 | 0 | 0 | 0 | 0 |
| XLOC_022175 | 1050.930103 | -0.691662074 | stratum II | 0 | 0 | 0 | 0 | 5 | 0 |
| XLOC_022176 | 257.1324265 | -1.014581677 | stratum II | 0 | 0 | 0 | 2 | 3 | 0 |
| XLOC_022177 | 1946.258809 | -0.937269475 | stratum II | 0 | 0 | 0 | 0 | 0 | 0 |
| XLOC_022178 | 3735.788993 | -0.974322579 | stratum II | 0 | 0 | 0 | 0 | 0 | 0 |
| XLOC_022179 | 31.2396338 | -0.66773304 | stratum II | 0 | 0 | 0 | 0 | 0 | 0 |
| XLOC_022180 | 89.44576046 | -0.938064144 | stratum II | 0 | 0 | 1 | 0 | 5 | 6 |
| XLOC_022675 | 3.104561513 | -0.365237175 | stratum II | 0 | 1 | 0 | 0 | 0 | 0 |
| XLOC_022676 | 62.58439601 | -1.25618281 | stratum II | 1 | 1 | 0 | 0 | 0 | 0 |
| XLOC_022181 | 985.2569193 | -1.070097648 | stratum II | 0 | 0 | 0 | 0 | 0 | 0 |
| XLOC_021651 | 12.73326404 | -0.752671056 | stratum II | 0 | 0 | 11 | 0 | 0 | 30 |
| XLOC_022677 | 4.092696913 | -0.302079701 | stratum II | 0 | 0 | 0 | 0 | 0 | 0 |
| XLOC_022678 | 2.913610019 | -0.729069597 | stratum II | 0 | 0 | 0 | 0 | 0 | 0 |
| XLOC_022182 | 8.912329013 | -0.933491331 | stratum II | 0 | 0 | 0 | 0 | 0 | 0 |
| XLOC_022679 | 5.796235331 | -0.820082477 | stratum II | 0 | 0 | 0 | 0 | 0 | 0 |
| XLOC_022680 | 4.259485654 | -0.42541588 | stratum II | 0 | 0 | 0 | 1 | 0 | 0 |
| XLOC_022681 | 44.21722225 | -0.940147598 | stratum II | 0 | 0 | 0 | 0 | 0 | 0 |
| XLOC_022682 | 2.807489391 | 0.143127276 | stratum II | 0 | 0 | 0 | 1 | 1 | 0 |
| XLOC_022683 | 7.539994359 | -0.629799373 | stratum II | 0 | 0 | 0 | 0 | 0 | 0 |
| XLOC_022183 | 2734.732123 | -0.93358892 | stratum II | 0 | 9 | 5 | 0 | 18 | 15 |
| XLOC_022684 | 17.52458336 | -0.132031145 | stratum II | 0 | 0 | 0 | 0 | 4 | 0 |
| XLOC_022685 | 9.82842018 | -0.630647741 | stratum II | 0 | 11 | 0 | 0 | 24 | 0 |
| XLOC_022686 | 8.155458149 | -0.531616274 | stratum II | 0 | 0 | 0 | 0 | 0 | 0 |
| XLOC_022687 | 43.87019562 | -0.481008766 | stratum II | 0 | 0 | 0 | 0 | 0 | 0 |
| XLOC_022688 | 21.73976221 | -0.627374059 | stratum II | 1 | 0 | 0 | 0 | 0 | 0 |
| XLOC_022689 | 3.65242154 | -0.59790641 | stratum II | 0 | 2 | 0 | 0 | 4 | 0 |
| XLOC_021652 | 15.05996547 | -0.462705118 | stratum II | 2 | 0 | 0 | 2 | 2 | 0 |
| XLOC_022690 | 37.85943802 | -0.79605793 | stratum II | 0 | 2 | 0 | 0 | 2 | 0 |
| XLOC_021653 | 55.11998969 | 0.680456988 | stratum II | 3 | 5 | 0 | 8 | 14 | 7 |
| XLOC_022691 | 6.439409866 | -0.4647827 | stratum II | 0 | 0 | 0 | 0 | 0 | 0 |
| XLOC_021654 | 40.10168621 | -0.330301485 | stratum II | 5 | 0 | 0 | 5 | 0 | 1 |
| XLOC_022692 | 4.131821588 | -0.494706552 | stratum II | 0 | 0 | 0 | 0 | 6 | 0 |
