# Supplementary material for: Spatial Dynamics of Evolving Dosage Compensation in a Young Sex Chromosome System
Source: Genome Biol Evol. 2015 Jan 23;7(2):581–90. doi: 10.1093/gbe/evv013 (PMC4350182; doi:10.1093/gbe/evv013)
Supplement: Supplementary Data [file supp_evv013_schultheiss_et_al_SUPPLEMENT_2.docx]

# SUPPLEMENT 2: Y gene calling

### Schultheiß et al.: Spatial dynamics of evolving dosage compensation in a young sex chromosome system

As described in the ‘Material and Methods’ section, we defined a SNP as being Y-specific when all four male sample pools where heterozygous at a locus whereas all four female sample pools were homozygous (the 4-sample case). We relaxed this criterion requiring only 3 males to be heterozygous at the locus under consideration (3-sample case) and eventually only 2 males to be heterozygous (2-sample case). For each of these cases we investigated a 1-SNP condition (i.e. 1 Y-specific SNP in a gene is sufficient to identify a gene as maintaining a Y copy) and a 4-SNP condition (4 Y-specific SNPs are required), respectively. Results of the 4-sample case (1 SNP condition) are discussed in the paper (see tables 1 and 2 there). Results for all other cases are provided below. Our analyses show that the 3-sample case does not change the number of Y genes and hence neither the differential expression analysis in comparison to the 4-sample case. The 2-sample case however increases substantially the number of “Y genes” in the recombining pseudoautosomal region and in the autosomes. Although the results of the expression analysis do still not change substantially, we interpret this strong increase of pseudo-“Y genes” as an indication of an increase in the false positive rate of detecting Y-genes. The following abbreviations are used in the subsequent tables:

- PAR: pseudoautosomal region
- SEX: sex chromosome (XIX)
- AUT: autosomes
- X-linked: set genes with no transcribed Y copy

### 4-sample case

#### 1-SNP condition (See tables 1 and 2 in the paper)

#### 4-SNP condition

Number and distribution of genes in chromosome XIX.

|  | SEX | PAR | stratum I | stratum II |
| --- | --- | --- | --- | --- |
| male-biased | 55 | 2 | 50 | 3 |
| female-biased | 488 | 1 | 158 | 329 |
| total | 1464 | 178 | 658 | 628 |
| density | 73.2 | 71.2 | 69.3 | 78.5 |
| Y genes | 143 | 0 | 125 | 18 |

Observed median ratios of gene expression between sex chromosomal and autosomal genes.

| region | male | female |
| --- | --- | --- |
| PAR:AUT | 1.047418 | 1.050097 |
| stratum I:AUT | 0.8628936 | 0.8874968 |
| stratum I(X-linked):AUT | 0.6814763 | 0.7429886 |
| stratum II:AUT | 0.8318896 | 1.2080256 |
| stratum II(X-linked):AUT | 0.8156749 | 1.1755229 |
| SEX:AUT | 0.8666826 | 1.0276989 |

### 3-sample case

#### 1-SNP condition

Number and distribution of genes in chromosome XIX.

|  | SEX | PAR | stratum I | stratum II |
| --- | --- | --- | --- | --- |
| male-biased | 55 | 2 | 50 | 3 |
| female-biased | 488 | 1 | 158 | 329 |
| total | 1464 | 178 | 658 | 628 |
| density | 73.2 | 71.2 | 69.3 | 78.5 |
| Y genes | 233 | 1 | 194 | 38 |

Observed median ratios of gene expression between sex chromosomal and autosomal genes.

| region | male | female |
| --- | --- | --- |
| PAR:AUT | 1.047418 | 1.050097 |
| stratum I:AUT | 0.8628936 | 0.8874968 |
| stratum I(X-linked):AUT | 0.5280584 | 0.5904835 |
| stratum II:AUT | 0.8318896 | 1.2080256 |
| stratum II(X-linked):AUT | 0.7921123 | 1.1419715 |
| SEX:AUT | 0.8666826 | 1.0276989 |

#### 4-SNP condition

Number and distribution of genes in chromosome XIX.

|  | SEX | PAR | stratum I | stratum II |
| --- | --- | --- | --- | --- |
| male-biased | 55 | 2 | 50 | 3 |
| female-biased | 488 | 1 | 158 | 329 |
| total | 1464 | 178 | 658 | 628 |
| density | 73.2 | 71.2 | 69.3 | 78.5 |
| Y genes | 143 | 0 | 125 | 18 |

Observed median ratios of gene expression between sex chromosomal and autosomal genes.

| region | male | female |
| --- | --- | --- |
| PAR:AUT | 1.047418 | 1.050097 |
| stratum I:AUT | 0.8628936 | 0.8874968 |
| stratum I(X-linked):AUT | 0.6814763 | 0.7429886 |
| stratum II:AUT | 0.8318896 | 1.2080256 |
| stratum II(X-linked):AUT | 0.8156749 | 1.1755229 |
| SEX:AUT | 0.8666826 | 1.0276989 |

### 2-sample case

#### 1-SNP condition

Number and distribution of genes in chromosome XIX.

|  | SEX | PAR | stratum I | stratum II |
| --- | --- | --- | --- | --- |
| male-biased | 55 | 2 | 50 | 3 |
| female-biased | 488 | 1 | 158 | 329 |
| total | 1464 | 178 | 658 | 628 |
| density | 73.2 | 71.2 | 69.3 | 78.5 |
| Y genes | 271 | 12 | 206 | 53 |

Observed median ratios of gene expression between sex chromosomal and autosomal genes.

| region | male | female |
| --- | --- | --- |
| PAR:AUT | 1.047418 | 1.050097 |
| stratum I:AUT | 0.8628936 | 0.8874968 |
| stratum I(X-linked):AUT | 0.5160538 | 0.5684242 |
| stratum II:AUT | 0.8318896 | 1.2080256 |
| stratum II(X-linked):AUT | 0.7664569 | 1.078334 |
| SEX:AUT | 0.8666826 | 1.0276989 |

#### 4-SNP condition

Number and distribution of genes in chromosome XIX.

|  | SEX | PAR | stratum I | stratum II |
| --- | --- | --- | --- | --- |
| male-biased | 55 | 2 | 50 | 3 |
| female-biased | 488 | 1 | 158 | 329 |
| total | 1464 | 178 | 658 | 628 |
| density | 73.2 | 71.2 | 69.3 | 78.5 |
| Y genes | 149 | 0 | 131 | 18 |

Observed median ratios of gene expression between sex chromosomal and autosomal genes.

| region | male | female |
| --- | --- | --- |
| PAR:AUT | 1.047418 | 1.050097 |
| stratum I:AUT | 0.8628936 | 0.8874968 |
| stratum I(X-linked):AUT | 0.6570307 | 0.7340983 |
| stratum II:AUT | 0.8318896 | 1.2080256 |
| stratum II(X-linked):AUT | 0.8156749 | 1.1755229 |
| SEX:AUT | 0.8666826 | 1.0276989 |
