# Supplementary material for: Spatial Dynamics of Evolving Dosage Compensation in a Young Sex Chromosome System
Source: Genome Biol Evol. 2015 Jan 23;7(2):581–90. doi: 10.1093/gbe/evv013 (PMC4350182; doi:10.1093/gbe/evv013)
Supplement: Supplementary Data [file supp_evv013_schultheiss_et_al_SUPPLEMENT_3.docx]

# SUPPLEMENT 3: dS Calculation

### Schultheiß et al.: Spatial dynamics of evolving dosage compensation in a young sex chromosome system

For investigating the synonymous substitution rate of X and Y genes, we compared them to an outgroup species (ninespine stickleback). The ninespine transcriptome was de novo assembled using Velvet v1.2.08 (Zerbino & Birney 2008) and Oases (Schulz et al. 2012) after which the transcripts were clustered using CD-HIT v 4.6.1 (Fu et al. 2012). Open reading frames were predicted for these clustered transcripts with TransDecoder (Haas et al. 2013).

We used the Basic Local Alignment Search Tool (BLAST, NCBI, (Altschul et al. 1990)) to perform reciprocal (blastx and tblastn) searches of peptide sequence of threespine stickleback transcripts against the predicted open reading frames of the ninespine transcripts. All ninespine transcripts that met the following citeria were kept for further analysis: (a) E-scores were less than 0.00001, (b) percentage of identity was greater than 50, (c) alignment length was at least 100 bases, and (d) the ninespine open reading frame, which has passed the previous steps, was matching to one threespine gene in both of the reciprocal searches. To obtain the alternative sequences, namely alternative ninespine and the threespine Y, we used the identified mutations from the ninespine and threespine transcriptomes with the FastaAlternateReferenceMaker tool in GATK (McKenna et al. 2010). Subsequently, the coding sequence (CDS) for the X and Y were extracted using our custom build GTF file and the program ‘gffread’ (provided with the TopHat package (Trapnell et al. 2012)). For the ninespine stickleback TransDecoder was run to obtain open reading frames with alternative sequences. CDS sequences from threespine X and Y, and open reading frames from ninespine were translated to peptides and aligned with MAFFT v.7 (Katoh et al. 2002). The multiple alignment was then back-translated and saved in phylip format prior to running ‘codeml’ in PAML (Yang 2007). We performed pairwise comparisons for all combinations among ninespine and ninespine alternative alleles, threespine X, and threespine Y.


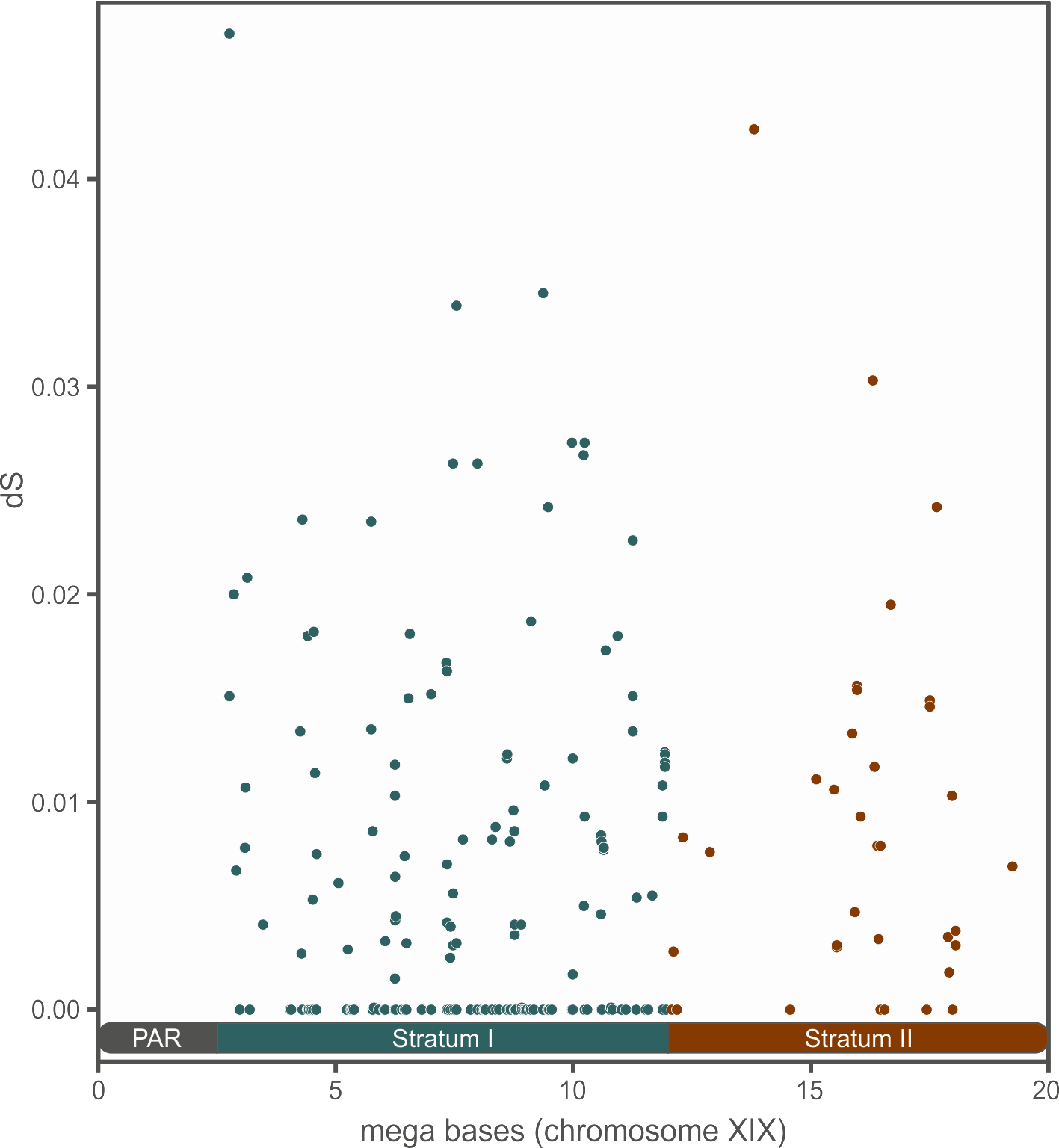


Figure S3.1

Spatial distribution and magnitude of dS values for the pairwise threespine X and threespine Y comparison. The following two data points are omitted from the figure for clarity: dS 4.1 at position 19,244,159 and dS 2.7 at position 18,047,766.

### References

Altschul SF, Gish W, Miller W, Myers EW, Lipman DJ. 1990. Basic local alignment search tool. Journal of Molecular Biology. 215:403–410. doi: [10.1016/S0022-2836(05)80360-2](http://dx.doi.org/10.1016/S0022-2836(05)80360-2).

Fu L, Niu B, Zhu Z, Wu S, Li W. 2012. CD-HIT: accelerated for clustering the next-generation sequencing data. Bioinformatics. 28:3150–3152. doi: [10.1093/bioinformatics/bts565](http://dx.doi.org/10.1093/bioinformatics/bts565).

Haas BJ et al. 2013. De novo transcript sequence reconstruction from RNA-seq using the Trinity platform for reference generation and analysis. Nat. Protocols. 8:1494–1512. doi: [10.1038/nprot.2013.084](http://dx.doi.org/10.1038/nprot.2013.084).

Katoh K, Misawa K, Kuma K-i, Miyata T. 2002. MAFFT: a novel method for rapid multiple sequence alignment based on fast Fourier transform. Nucl. Acids Res. 30:3059–3066. doi: [10.1093/nar/gkf436](http://dx.doi.org/10.1093/nar/gkf436).

McKenna A et al. 2010. The Genome Analysis Toolkit: A MapReduce framework for analyzing next-generation DNA sequencing data. Genome Res. 20:1297–1303. doi: [10.1101/gr.107524.110](http://dx.doi.org/10.1101/gr.107524.110).

Schulz MH, Zerbino DR, Vingron M, Birney E. 2012. Oases: robust de novo RNA-seq assembly across the dynamic range of expression levels. Bioinformatics. 28:1086–1092. doi: [10.1093/bioinformatics/bts094](http://dx.doi.org/10.1093/bioinformatics/bts094).

Trapnell C et al. 2012. Differential gene and transcript expression analysis of RNA-seq experiments with TopHat and Cufflinks. Nat. Protoc. 7:562–578. doi: [10.1038/nprot.2012.016](http://dx.doi.org/10.1038/nprot.2012.016).

Yang Z. 2007. PAML 4: Phylogenetic Analysis by Maximum Likelihood. Mol Biol Evol. 24:1586–1591. doi: [10.1093/molbev/msm088](http://dx.doi.org/10.1093/molbev/msm088).

Zerbino DR, Birney E. 2008. Velvet: Algorithms for de novo short read assembly using de Bruijn graphs. Genome Res. 18:821–829. doi: [10.1101/gr.074492.107](http://dx.doi.org/10.1101/gr.074492.107).
